# Estimates of the Direct Effect of Seawater pH on the Survival Rate of Species Groups in the California Current Ecosystem

**DOI:** 10.1371/journal.pone.0160669

**Published:** 2016-08-11

**Authors:** D. Shallin Busch, Paul McElhany

**Affiliations:** 1 Ocean Acidification Program, Office of Oceanic and Atmospheric Research and Northwest Fisheries Science Center, National Marine Fisheries Service, National Oceanic and Atmospheric Administration, Seattle, Washington, United States of America; 2 Conservation Biology Division, Northwest Fisheries Science Center, National Marine Fisheries Service, National Oceanic and Atmospheric Administration, Seattle, Washington, United States of America; Stony Brook University, UNITED STATES

## Abstract

Ocean acidification (OA) has the potential to restructure ecosystems due to variation in species sensitivity to the projected changes in ocean carbon chemistry. Ecological models can be forced with scenarios of OA to help scientists, managers, and other stakeholders understand how ecosystems might change. We present a novel methodology for developing estimates of species sensitivity to OA that are regionally specific, and applied the method to the California Current ecosystem. To do so, we built a database of all published literature on the sensitivity of temperate species to decreased pH. This database contains 393 papers on 285 species and 89 multi-species groups from temperate waters around the world. Research on urchins and oysters and on adult life stages dominates the literature. Almost a third of the temperate species studied to date occur in the California Current. However, most laboratory experiments use control pH conditions that are too high to represent average current chemistry conditions in the portion of the California Current water column where the majority of the species live. We developed estimates of sensitivity to OA for functional groups in the ecosystem, which can represent single species or taxonomically diverse groups of hundreds of species. We based these estimates on the amount of available evidence derived from published studies on species sensitivity, how well this evidence could inform species sensitivity in the California Current ecosystem, and the agreement of the available evidence for a species/species group. This approach is similar to that taken by the Intergovernmental Panel on Climate Change to characterize certainty when summarizing scientific findings. Most functional groups (26 of 34) responded negatively to OA conditions, but when uncertainty in sensitivity was considered, only 11 groups had relationships that were consistently negative. Thus, incorporating certainty about the sensitivity of species and functional groups to OA is an important part of developing robust scenarios for ecosystem projections.

## Introduction

Global oceans are changing due to the anthropogenically driven increase in atmospheric carbon dioxide concentrations [1,2,3]. Ocean acidification, the decline in pH and calcium carbonate saturation state due to increased dissolved carbon dioxide, has the potential to alter marine ecosystems, as indicated by the fossil record [4], modeling exercises [5,6], and contemporary laboratory and field observations. Modern day evidence from carbon dioxide (CO_2_) vent sites documents community changes with increased CO_2_, such as a decline in calcifying species and increase in non-calcifying, photosynthetic species [7,8,9]. In areas with upwelling of high CO_2_/low pH waters, research has shown the sensitivity of the pteropod *Limacina helicina* and the Pacific oyster *Crassostrea gigas* to current carbon chemistry conditions [10,11,12]. Given the sensitivity of many modern species to carbonate chemistry conditions in the laboratory, especially bivalves and corals [13,14,15], we expect the effects of ocean acidification on marine species and ecosystems to increase.

The majority of biological research related to ocean acidification focuses on direct effects on species, mostly in laboratory experiments. However, societal questions related to ocean acidification are much larger, scaling to concerns about food webs, ecosystems, and provisioning from marine resources [16]. Projections of ocean acidification impacts generated with ecological models can help develop understanding of the indirect effects of ocean acidification at large scales [5,17,18]. The utility of the output from such projection exercises is, among other things, dependent on how well the scenarios imposed on the model capture the direct effects of ocean acidification. Our ability to scale from results of laboratory studies to trajectories of populations in nature is currently limited for a variety of reasons [18,19,20]. For example, laboratory experiments are typically conducted on species in isolation with altered food and other environmental conditions, and usually limited to a few weeks. Thus, all but a handful of studies (e.g., [21,22,23]) do not capture multi-generation processes, such as adaptation and evolution. Furthermore, the published literature on species response to carbonate chemistry is small enough that one cannot develop comprehensive scenarios of acidification for a region based only on its native species and locally relevant current and projected future carbon chemistry treatments [24,25], thus forcing those developing scenarios to relate studies from around the globe to their region of interest. The limited literature on species response to carbonate chemistry also means that modelers will not have access to OA effects on the parameters most relevant to ecological models (e.g., survival or growth rate over an entire life stage), which requires that scenario developers relate results reported in the literature (e.g., calcification or respiration rate, change in gene expression) to the parameters that they can alter in the ecological model. Finally, most ecological models simulate the dynamics of groups of aggregated species, typically referred to as functional groups, instead of modeling each species in the ecosystem independently. Functional groups are often heterogeneous taxonomically, especially lower trophic level functional groups. The sensitivity of species in a given functional group to carbonate chemistry may vary widely in both direction and magnitude, which highlights the challenge of characterizing the response of a functional group as a whole.

Our goal is to calculate the direction and relative magnitude of carbonate chemistry sensitivity of marine functional groups. Here we present an approach for developing estimates of the carbonate chemistry sensitivity of species or functional groups for use in scenarios of ocean acidification in ecological modeling exercises and apply this approach to functional groups in the California Current ecosystem. The aim of our approach is to summarize the weight and consistency of evidence for pH sensitivity of biota in the California Current, doing so by borrowing heavily from research in other regions on taxonomically distant species. We use this information to create a relative survival scalar, which is a value relating a functional group’s survival rate parameter to the pH condition of its environment. The relative survival scalar allows us to qualitatively rank groups in terms of their sensitivity to acidification. We focus on the qualitative nature of pH sensitivity rather than a formal meta-analysis of quantitative response. Although experiments show that some species are sensitive to calcium carbonate saturation state rather than pH *per se* [26], we conducted our analysis on pH sensitivity because pH is the parameter most commonly manipulated in published experimental studies and it tends to correlate tightly with other biologically relevant carbonate chemistry conditions in seawater (e.g., carbonate saturation state and pCO_2_). Our approach develops a technique for sound incorporation of information of varied relevance to the California Current ecosystem and the ecosystem models used to study it. Furthermore, our approach explicitly addresses uncertainty in understanding of species response to acidification.

In brief, we developed a database of all of the studies that document the response of temperate species to carbonate chemistry conditions that is current to January 1, 2015. This database includes information about the species studied, treatment conditions, the type of responses measured (e.g., survival, respiration, etc.), the direction of response related to increased acidification, and the relationship between directional response and population persistence. Here, we consider population persistence as the ability to, at least, maintain abundance in succeeding generations, through survival and reproductive success. Our approach is qualitative in nature, but allows translation of reported results from all studies into a common quantitative currency (population persistence) required for ecosystem modeling.

We used the database to develop estimates of species sensitivity to carbonate chemistry for use in scenarios in an end-to-end ecosystem model of the California Current [27,28,29]. Output from scenario-based, ecosystem modeling exercises can provide valuable input into ecosystem and fisheries management and decision making by providing science-based estimates of alternative futures as part of management strategy evaluations [30,31]. Due to its position at the end of the ocean conveyer belt and in an upwelling region, the California Current is a region particularly prone to acidification [32,33]. It is also rich in marine resources, supporting valuable fisheries, recreation, and tourism industries [34]. Many of the region’s commercial fisheries are potentially directly susceptible to the impacts of ocean acidification (e.g., Dungeness crab (*Cancer magister*) [35], pink shrimp (*Pandalus jordani*) [36], bivalve aquaculture [10]) and others to the indirect impacts that may ripple through the food web.

Our approach for developing estimates of carbonate chemistry sensitivity for the California Current ecosystem model emphasizes the relevance of studies to local species and ecology. As discussed by McElhany and Busch [24] and elsewhere, robust predictions of the effects of acidification on species require that control and treatment conditions well represent local current and projected future conditions, ideally with fluctuations in conditions that resemble local patterns in chemistry caused by tidal, diel, and other cycles. Observational studies, for instance from CO_2_ vent sites with naturally acidified conditions, capture important aspects of ecological and population-level processes that are difficult to replicate in the laboratory, yielding more valuable information on species response in nature. Distinct from other global syntheses and meta-analyses [13,14,15,37], for this synthesis, we use criteria related to experimental design to weight how strongly a study informs the estimates of acidification in a particular region. For example, a 3-month study conducted on early life stages of a species distributed in and collected from the California Current that measured survival and growth in response to ecologically relevant control conditions would have more weight in the estimate than a week-long study that measures proteomics in adults of a species found only in the Mediterranean Sea.

The methodology used to develop estimates acknowledges that the study of ocean acidification is a young field and, thus, we are still rapidly generating basic information to describe how and why species respond to carbonate chemistry conditions in the manner observed in the laboratory and in the wild [18]. Currently, the literature suggests that the response to changing carbonate chemistry can vary among experiments conducted on a single species, sometimes by population [38], or species that are closely related [39,40]. Until we understand what drives species response to carbonate chemistry conditions, characterizing variation and confidence in response is an important component of summarizing sensitivity for species or groups of species. This challenge of accurately portraying the state of our knowledge is also faced by those working on climate change, where it has received much attention by the Intergovernmental Panel on Climate Change (IPCC). The IPCC has developed a formal approach for characterizing certainty in scientific findings related to evidence and agreement [41,42,43,44], and we build off of this accepted and standardized approach in the synthesis methodology presented here.

## Materials and Methods

### Database

We built a database of laboratory, mesocosm, and field studies on the response of temperate species to carbonate chemistry conditions (S1 and S2 Databases). To search the literature, we used ISI Web of Science and relevant keywords such as ocean acidification, pH, and different names for various marine taxa. We used the European Project on Ocean Acidification (EPOCA) OA blog (http://oceanacidification.wordpress.com/) and citations in published studies to find primary literature not captured in our queries of Web of Science. We included studies published or pre-released before January 1, 2015. Studies conducted in non-temperate locations on species that exist in temperate regions were included in the database, except for tropical, reef-forming coral species, as only cold-water corals occur in the California Current ecosystem. Studies using HCl or NaOH only to manipulate seawater chemistry were not included in the database because of the differences in carbon chemistry between this type of chemistry manipulation and manipulations using carbon dioxide [45]. The database includes two main sets of information:

- For each species included in the database, we recorded taxonomic classification, distribution, presence/absence of calcium carbonate structures, and functional group in the California Current Atlantis ecosystem model [27] (functional groups described below).
- For each manuscript included in the database, we recorded the 1) citation, 2) location where study subjects were collected, 3) methodology(ies) used to create and verify carbon chemistry treatments, 4) carbon chemistry of treatments, 5) species response to treatments (direction of response [e.g., growth decreases as pH decreases], not raw data), and 6) assessment of how the direction of response relates to population persistence.

Each **paper** in the database could have multiple studies, where a **study** was a particular set of treatments applied to particular species and life stage. Each study in turn could have multiple **response metrics**, where a response metric is a measure of the species performance under treatment (e.g., survival, metabolic rate, proteomic expression). A **response** refers to a result from evaluation of a particular response metric in a particular study. The **response** is the basic unit for our analysis.

### Summarizing sensitivity to carbonate chemistry

For this study, we considered the California Current ecosystem, and so adopted functional groups defined by the California Current Atlantis ecosystem model ([27]; S1 Table). The Atlantis model domain extends from Triangle Island, off the north coast of Vancouver Island, British Columbia Canada, to Punt Eugenia, Baja California, Mexico; the shoreline to 200 nautical miles offshore; and the surface to the ocean floor. The Atlantis model excludes inland areas such as the Salish Sea and San Francisco Bay, but species composition in these regions overlaps with adjacent areas of the Atlantis domain. The Atlantis model does not capture the dynamics of exclusively inter-tidal species and communities. The functional groups used in the California Current Atlantis model reflect the model’s initial focus on fisheries management and the limited data available for model parameterization (i.e., abundance, prey consumption). These practical considerations led to somewhat eclectic groupings from a purely ecological or taxonomic perspective (e.g., the “Humboldt squid” functional group is a single species, while the “Large phytoplankton” functional group contains an ecologically diverse multitude of species). We excluded birds and marine mammals from this analysis as we did not find any studies documenting direct sensitivity to carbonate chemistry (OA effects on sound propagation are unlikely to harm marine mammals [46]). For our analysis, we created a “fish” group, which is a combination of all 31 bony fish functional groups in the ecosystem model. We did so because fish functional groups in this ecosystem model are highly resolved, more so than other functional groups in the model, and because literature on fish’s sensitivity to carbonate chemistry does not match the model’s level of specificity. Because multiple studies suggest that coralline algae are sensitive to carbonate chemistry, we included them as a functional group in our analysis, even though coralline algae are not a functional group in the Atlantis model.

Our basic approach was to develop a **survival scalar** for each functional group that characterizes the relationship between survival and pH. Each survival scalar was based on a **directional score** and a **confidence score** (S1 Fig). The confidence score was composed of two components: **evidence** and **agreement scores**. All of these terms are defined below. The evidence/agreement approach to confidence was founded on the IPCC’s guidelines for characterizing certainty in scientific findings [41,42,43,44].

The foundation for the directional, evidence, and agreement scores was the **response score** (shorted to **rScore**). **rScores** are a weighted measure on a 0–1 scale of how well each response relates to survival of a functional group in the California Current Atlantis ecosystem model. An “ideal” response (e.g., well-conducted experiment on organisms collected in the California Current that directly measures long-term survival, etc.) would receive an rScore of 1. Most responses require some level of extrapolation to apply them to survival of a functional group in the California Current model (e.g., the experiment was conducted on a related species that does not occur in the California Current; the response metric was not immediately translatable into survival; etc.). The rScore for each response was calculated as the average of the component relevance values of 8 different categories of information in the database, each on a 0–1 scale: study environment, exposure duration, collection location, ability to measure population persistence, control pH treatment, minimum experimental pH treatment, relatedness to species in the California Current, distribution relative to the California Current, and response type. The scoring system for converting database entries into 0–1 component relevance values is in Table 1. Each rScore was assigned a direction to define whether exposure to high CO_2_ conditions resulted in an increase or decrease in population persistence, had no effect on population persistence, or if there was a more complex relationship. This complex relationship category is a combination of several subcategories, such as a category where the sign of response to CO_2_ depends on a covariable. rScores in the complex relationship category were relatively rare and were not used in the analysis. For some studies, the relationship to population persistence could not be determined because there was no clear relationship between the response metric and population persistence.

**Table 1:** Scoring system used to define the relevance of a response to informing sensitivity of California Current species to changes in survival with decreased ocean pH.

Rule type	Rule set	Score
**Study environment**		
	If study took place at a natural CO_2_ vent site or takes advantage of a natural gradient in CO_2_ conditions	1
	If study took place in a laboratory on multiple species living in the same experimental chamber	0.75
	All else	0.5
**Ability to measure population persistence**		
	If the response has a known relationship to population persistence	1.0
	All else	0.5
**Control pH treatment**		
	If pH treatment ≥ 8.1	0.5
	If pH treatment ≥ 7.8, < 8.1	1.0
	If pH treatment < 7.8	0.1
**Minimum experimental pH treatment**		
	If pH treatment > 7.8	0.5
	If pH treatment ≤ 7.8	1.0
**Relatedness to species in the California Current**^a^		
	If species lives in the California Current	1.0
	If the genus but not species lives in the California Current	0.75
	If the family but not genus lives in the California Current	0.5
	If the order but not family lives in the California Current	0.25
	All else	0.1
**Distribution in the California Current**		
	If the species occurs in the California Current	1.0
	If the species does not occur in the California Current	0.25
**Collection location**		
	If study subjects were collected in the California Current	1.0
	If study subjects were collected close to the California Current	0.75
	If study subjects were not collected in the California Current or if collection location is unspecified	0.25
**Response type**		
	If study evaluates decomposition, genomic response, harmful algal bloom variables, nitrogen fixation, proteomic response, viral shedding	0.1
	If study evaluates behavior, body composition, immune function, morphology	0.2
	If study evaluates acid-base balance, metabolism	0.5
	If study evaluates calcification	0.8
	If study evaluates development, growth, photosynthesis, reproductive rate, survival	1.0
**Exposure duration**		
	If gamete, fertilized egg, spore	1.0
	If early life stage of phytoplankton	
	≥ 6 hours	1.0
	< 6 hours	0.5
	If early life stage of macroalgae, seagrass	
	≥ 5 days	1.0
	< 5 days	0.5
	If larvae/early life stage of abalone, krill, copepod, polychaete	
	≥ 6 days	1.0
	< 6 days	0.5
	If larvae/early life stage of crab	
	≥ 30 days	1.0
	< 30 days	0.5
	If larvae/early life stage of other invertebrates or vertebrates	
	≥ 14 days	1.0
	< 14 days, ≥ 7 days	0.66
	< 7 days	0.33
	If juvenile stage of macroalgae or seagrass	
	≥ 30 days	1.0
	< 30 days, ≥ 7 days	0.66
	< 7 days	0.33
	If juvenile stage of invertebrate	
	≥ 30 days	1.0
	< 30 days, ≥ 7 days	0.66
	< 7 days	0.33
	If juvenile stage of vertebrate	
	≥ 90 days	1.0
	< 90 days	0.5
	If adult stage of phytoplankton, bacteria, copepod	
	≥ 5 days	1.0
	< 5 days	0.5
	If adult stage of macroalgae, seagrass, invertebrate	
	≥ 50 days	1.0
	< 50 days, ≥ 10 days	0.66
	< 10 days	0.33
	If adult stage of vertebrate	
	≥ 100 days	1.0
	< 100 days	0.5
	If duration undefined	0.33

^a^. To assess relatedness of species in the database that do not occur in the California Current to species in the California Current, we used a database of species in Puget Sound (Busch and McElhany, in preparation) to define which genera, families, and orders exist in the California Current. Puget Sound does not contain all of the species in the California Current domain, but was the most comprehensive regional species list available to us. The lack of a complete domain-wide species list may have resulted in an underestimate of the level of relatedness for some study species, particularly those from southern parts of the domain.

#### Directional score

The **directional score** for function group *i* is the net directional effect of exposure to high CO_2_ as the fraction of total rScores:

$$directional\;score_{i}=PrScore_{increase}-PrScore_{decrease}$$

where,

$$PrScore_{x}=\;\frac{\sum rScore_{x}}{\sum rScore_{increase}+\sum rScore_{decrease}+\sum rScore_{no\;effect}}$$

*x* is either the category “increase” or “decrease”, and summations are over all of the rScores for a given functional group *i*.

By using rScores, the directional score gives the overall level of support for a directional effect when responses are weighted by the information they contain relative to survival of species in the California Current. The directional score is a continuous metric from -1 to +1, with –1 indicating all studies supporting a decrease in population persistence with increased CO_2_ and +l indicating all studies supporting higher population persistence with increased CO_2_. A directional score of zero indicates an equal fraction of rScores in the increasing and decreasing population persistence categories. An increasing fraction of rScores in the no effect population persistence category or opposing population persistence responses will cause the directional score to tend toward zero.

#### Evidence score

The **evidence score** for functional group *i* is the sum of all rScores for the functional group regardless of population persistence response category:

$$evidence\;score_{i}=\sum rScore_{increase}+\sum rScore_{decrease}+\sum rScore_{no\;effect}$$

It is a measure of the overall amount of information available relative to the effect of CO_2_ on survival of the functional group. The evidence scores can be considered a count of the total number of responses for each functional group weighted by the relevance of the response to survival of species in the California Current. Thus, the evidence score contains information on both quantity and quality of available data.

#### Agreement score

The **agreement score** measures similarity within a functional group in its response to increased CO_2_. It is a normalized, weighted metric based on the probability of drawing a pair of responses with the same direction from the set of functional group responses, and is similar to diversity indices. The raw agreement score is:

$$\begin{array}{l} raw\;agreement\;\;score_{i}=a\left(PrScore_{increase}^{2}+PrScore_{decrease}^{2}+PrScore_{no\;effect}^{2}\right)\\ +b\left(2*PrScore_{increase}*PrScore_{no\;effect}+2*PrScore_{decrease}*PrScore_{no\;effect}\right)\\ +c\left(2*PrScore_{increase}*PrScore_{decrease}\right) \end{array}$$

where the first term of the function is the weighted probability that two random rScores are the same, the second term is the weighted probability that one random rScore is “no response” and the other is either “increasing” or “decreasing”, and the third term is the weighted probability that one random rScore is “increasing” and the other is “decreasing”. We weighted the terms because the population persistence categories are ordered, with the “no effect” category being between the “increase” and “decrease” categories. To capture this weighing, *a* is given a value of 1 indicating high consistency, *b* a value of 0.5 to reflect that a “no effect” response falls between an increase and a decrease and is of intermediate agreement, and *c* a value of 0 to indicate the opposite responses do not agree. Values for the raw agreement score can range between 0.5–1. To scale these from 0–1, we normalized their values:

$$agreement\;score_{i}=\left(raw\;agreement\;score_{i}*2\right)-1$$

With this normalization, if the agreement score for the function group is 1, then all rScores are the same direction; if the agreement score for the functional group is 0.5, then the summed rScore value for no effect is the same as for either increase or decrease responses; and if the agreement score for the function group is 0, then the summed rScore values for increase and decrease responses are identical.

#### Survival scalar

**Survival scalars** describe the direction and degree to which survival is affected by increased CO_2_, where the survival scalar for functional group *i* is:

$$survival\;scalar_{i}=directional\;score_{i}*confidence\;score_{i}$$

and the **confidence score** for the functional group is:

$$confidence\;score_{i}=\text{ln}\left(evidence\;score_{i}\right)*agreement\;score_{i}$$

We ln-transform the evidence score in the confidence score equation to prevent a few functional groups with very large evidence scores from dominating the normalization process. Using the confidence score to calculate the survival scalar for a functional group directly incorporates measurements of certainty into the scalar.

The **relative survival scalar** is a normalized version of the survival scalar: the smallest survival scalar of all the functional groups was given a value of -1, and other relative survival scalars were assigned as:

$$relative\;survival\;scalar_{i}=\;\frac{survival\;scalar_{i}}{\left|survival\;scalar_{min}\right|}$$

### Estimates of CO_2_ sensitivity for the California Current ecosystem model

The **relative survival scalars** give relative sensitivity and direction of functional groups to carbonate chemistry conditions. However, they cannot be used directly as part of scenarios of ocean acidification in the California Current Atlantis model [31], as they are not in the proper form to input into the model. As a case study to show how the relative survival scalars could be used to inform scenarios of ocean acidification that drive response to pH in an ecosystem model, we developed **pH survival sensitivity curves**, which have pH on the x-axis and **functional group pH sensitivity factor** on the y-axis. The pH survival sensitivity curves can be used to modify functional group survival under scenarios of acidification, potentially by multiplying baseline survival in the model for a functional group by the value on the curve that corresponds to local pH conditions.

**Fig 1:**
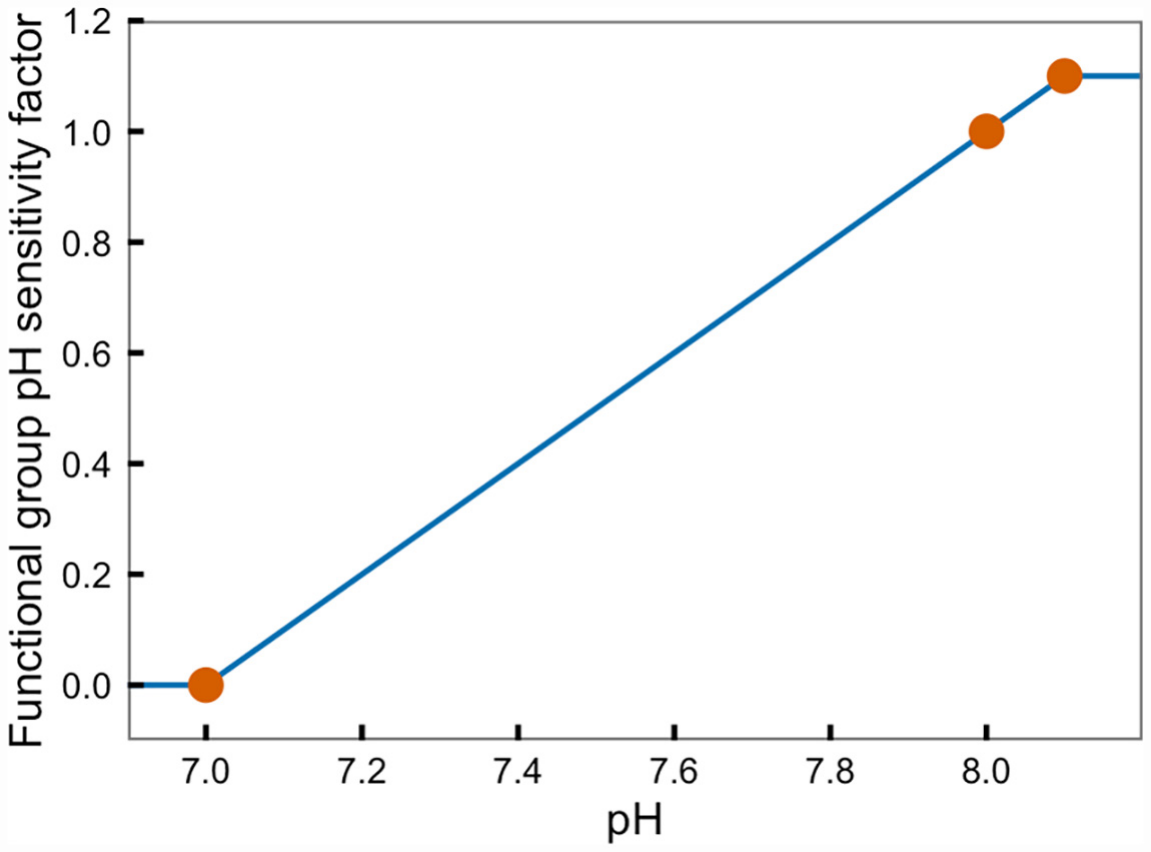
Reference curve. Reference curve for the survival response of a generic functional group to pH conditions.

To generate **pH survival sensitivity curves**, we defined a “reference relationship” between pH and species survival sensitivity and used the relative survival scalars to modify this reference relationship to account for the differential effect of pH among the various functional groups (Fig 1). We based the reference relationship on the estimated sensitivity of the most negatively responding functional group in the model–in this case, benthic herbivorous grazers, which is comprised of snails, abalone, sand dollars, limpets, spot prawn, and non-fishery urchins. We chose a “hockey stick” shape for the reference relationship, which is one of a limited number of relationship shapes the Atlantis ecosystem model can accept for pH sensitivity relationships. To define the reference relationship, we assumed that at current average pH conditions in the California Current over the decade from 2011–2020 (pH 8.0, Marshall et al., in review), survival would be identical to the current baseline model parameterization (i.e., OA survival sensitivity factor = 1). We held this constant for all functional groups. After reviewing the published data on response to CO_2_ in the database we built, we set pH 7.0 as the point at which survival sensitivity factor of benthic herbivorous grazers reaches zero (i.e., survival of benthic herbivorous grazers would drop to zero below pH 7). These two points (survival sensitivity factor of 1 at pH 8.0 and survival sensitivity factor of 0 at pH 7.0) define the **reference slope** of the hockey stick function for the reference relationship (Fig 1). We assumed that survival would be slightly higher at pH conditions higher than average because calcification and other processes can be favored at higher pH, which corresponds to a higher calcium carbonate saturation state. We assumed that the maximal survival sensitivity factor would be 1.1, reflecting a maximum 10% increase in survival under high pH conditions for all functional groups. Here, survival is a unitless scalar which can be translated to the time step of different ecological models, as appropriate. To generate the survival response relationships for all other functional groups in the California Current Atlantis ecosystem model, we multiplied this reference slope by the functional groups’ survival scalars, allowing the curve to pivot around the average conditions point (at pH 8.0, pH sensitivity factor = 1) with a minimum survival sensitivity factor value of zero and a maximum survival sensitivity factor of 1.1 when pH > 8.0. Functional groups that increase survival with increased CO_2_ were allowed to have sensitivity factors greater than 1.1 at low pH to reflect potential increased productivity for these groups.

We used the confidence score to generate “high” and “low” pH sensitivity estimates to provide some uncertainty bounds on the ecosystem model output. These alternate estimates were based on changing the pH survival sensitivity curves using the confidence score for each functional group to manipulate the slope of the hockey-stick relationship. We call this **slope error** and calculate it by first normalizing the confidence score of each functional group *i* and reversing the scale so that the functional group with the greatest confidence score has a **slope adjustment coefficient** of zero:

$$slope\;adjustment\;coefficient_{i}=1-\frac{confidence\;score_{i}}{confidence\;score_{max}}$$

The slope error is then calculated as:

$$slope\;error_{i}=\pm \left((s_{1}*slope\;adjustement\;coefficient_{i}\right)+s_{2})$$

The *s*_*2*_ parameter describes the baseline slope error for the “best” functional group that has the least error (i.e., when the slope adjustment coefficient = 0). The sum of *s*_*1*_ and *s*_*2*_ is the maximum error if a functional group had a confidence score of zero (i.e., slope adjustment coefficient = 1). For initial estimates, we tested cases where *s*_*1*_ and *s*_*2*_ were set at 0.2 and 0.3, respectively. Slope error can be added and subtracted from the pH survival sensitivity curves to generate high and low sensitivity estimates, respectively. This approach should be interpreted as providing a relative assessment of curve confidence among functional groups that is suitable for gaming scenarios with the ecosystem model. However, the approach ignores many other potential sources of uncertainty to the pH survival sensitivity curves.

## Results

### Literature on carbonate chemistry sensitivity studies

**Fig 2:**
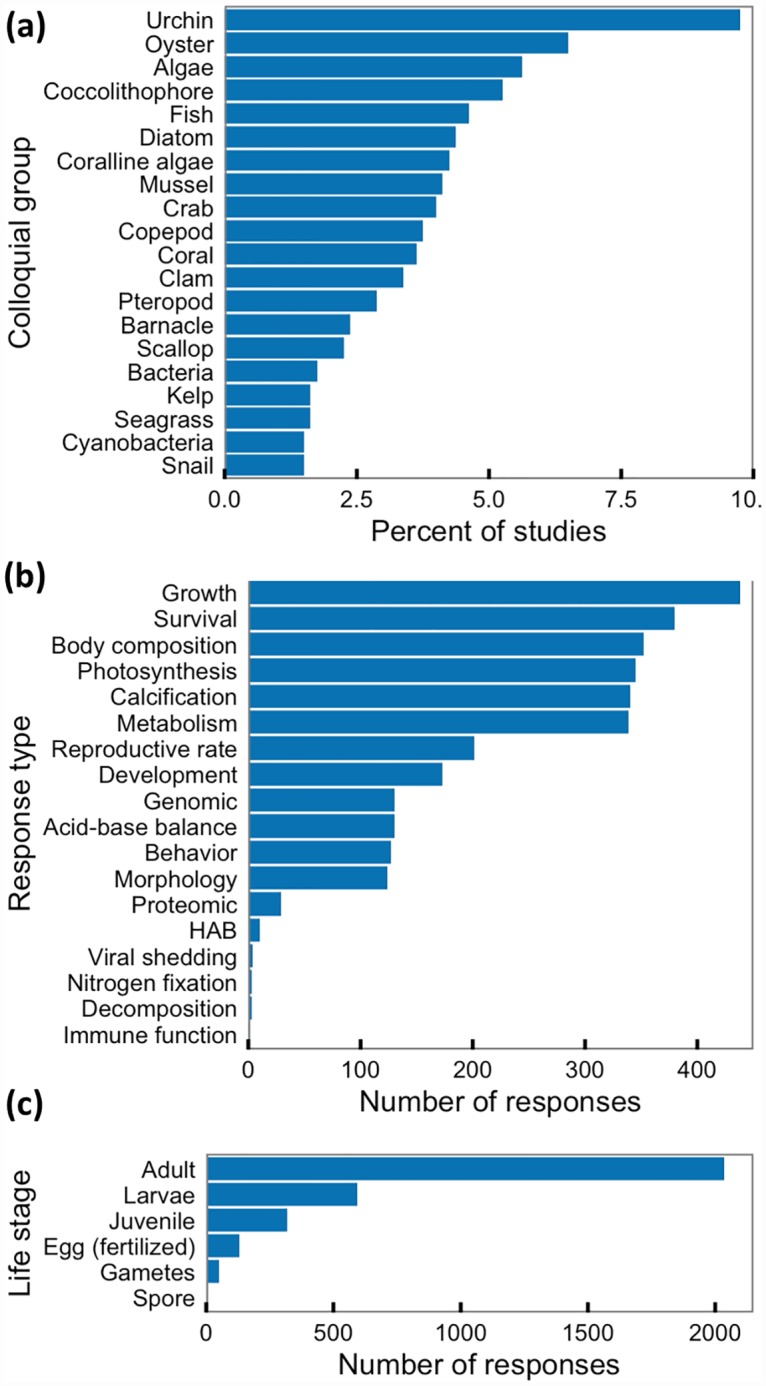
Description of database content. (a) Percent of studies in the database of literature on species sensitivity to carbonate chemistry conditions by colloquial species group. We use colloquial groups (e.g., urchin, fish, coral), instead of taxonomy or Atlantis functional groups, to guide groupings in Fig 2a because of management and research interest in these groups. The top twenty groups shown here comprise 75% of the studies in the database. Number of responses for (b) each response type and (c) life stage in the database of literature on species response sensitivity to carbonate chemistry conditions.

The database contains 393 papers, 847 studies, and 3,158 responses on the sensitivity of temperate species to carbonate chemistry conditions. Response to carbonate chemistry conditions was characterized for 285 species and 89 multi-species groups in 220 genera, 185 families, 100 orders, and 36 classes. Seventy-five percent of these studies were done on twenty species groups, with the greatest number of studies done on urchins and oysters (Fig 2a). The most frequently measured response parameters were related to growth (14%) and survival (13%), though many responses were related to body composition, metabolism, photosynthesis, and calcification (each 11%; Fig 2b). The majority of studies used adults as study subjects (64%, Fig 2c). Juvenile and larval life stages were also well represented as study subjects (19% and 11%, respectively).

**Fig 3:**
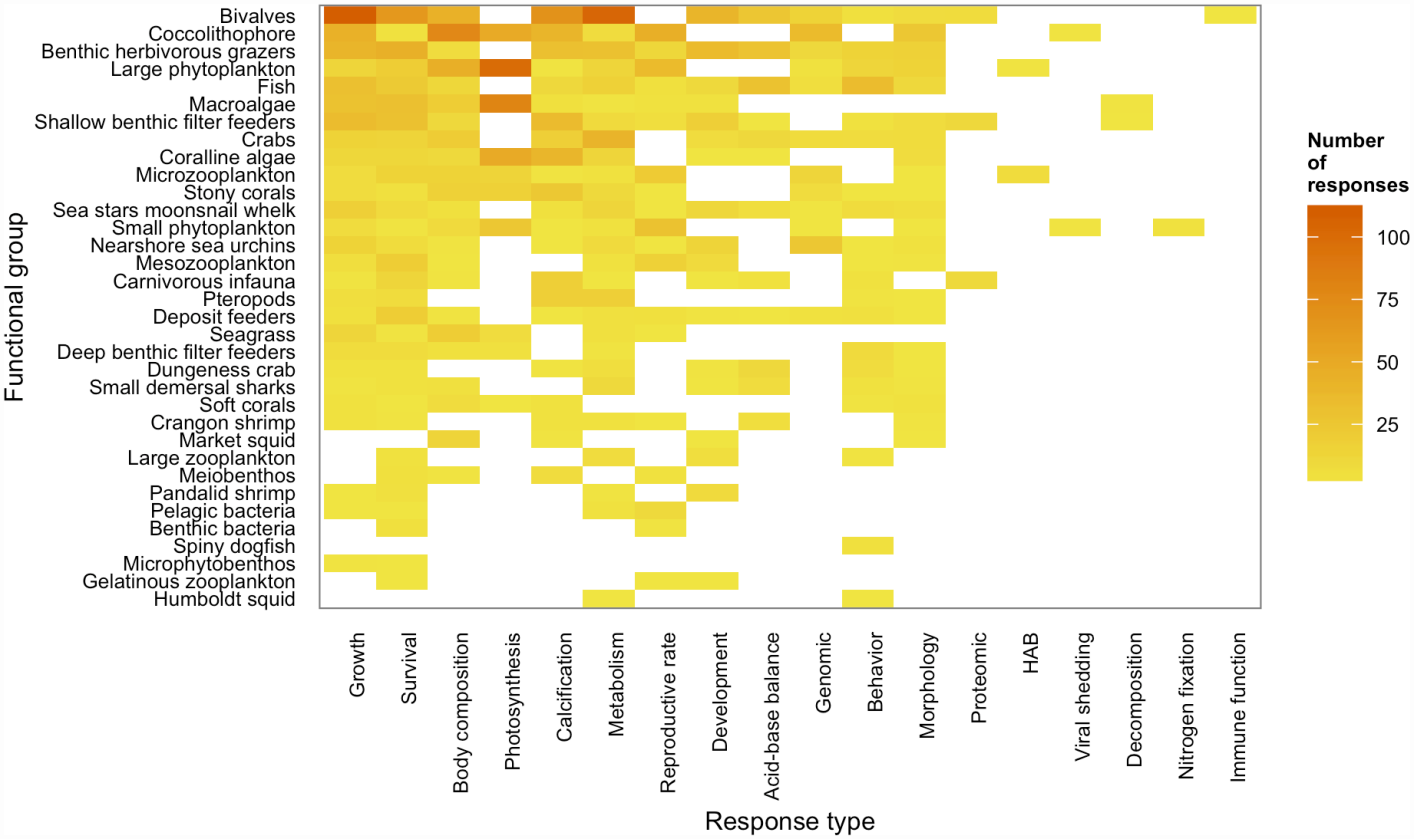
Response types for each functional group. The number of responses for each response type for each functional group. The darker the cell, the more responses for the response type, with dark orange being 100 and bright yellow being 0.

Almost a third of the species included in the database occur in the California Current ecosystem (29% native species, 2% introduced species; S2 Fig). However, a large portion of the database of study species was comprised of multispecies groups and poorly studied phytoplankton or bacteria (26%); we were unable to determine whether these species occur in the California Current. While California Current species were well represented in the database, study subjects collected from the California Current comprised a smaller portion of the responses (13%; S2 Fig). This result suggests that published papers on species from the California Current include fewer response metrics (e.g., calcification, respiration rate, gene expression) than published papers on species from other parts of the world. The number of responses for each functional group in the Atlantis ecosystem is highly uneven, with the most numerous responses for the following functional groups: bivalves (17%, 12 response types), coccolithophores (11%, 10 response types), benthic herbivorous grazers (9%, 11 response types), and large phytoplankton (9%, 11 response types; Fig 3). A major gap in the literature is the lack of any published studies characterizing the sensitivity of 6 functional groups to carbonate chemistry (octopus, cephalopod (which excludes Humboldt squid (*Dosidicus gigas*) and market squid (*Doryteuthis opalescens*)), large demersal sharks, miscellaneous pelagic sharks, skates and rays, black coral).

**Fig 4:**
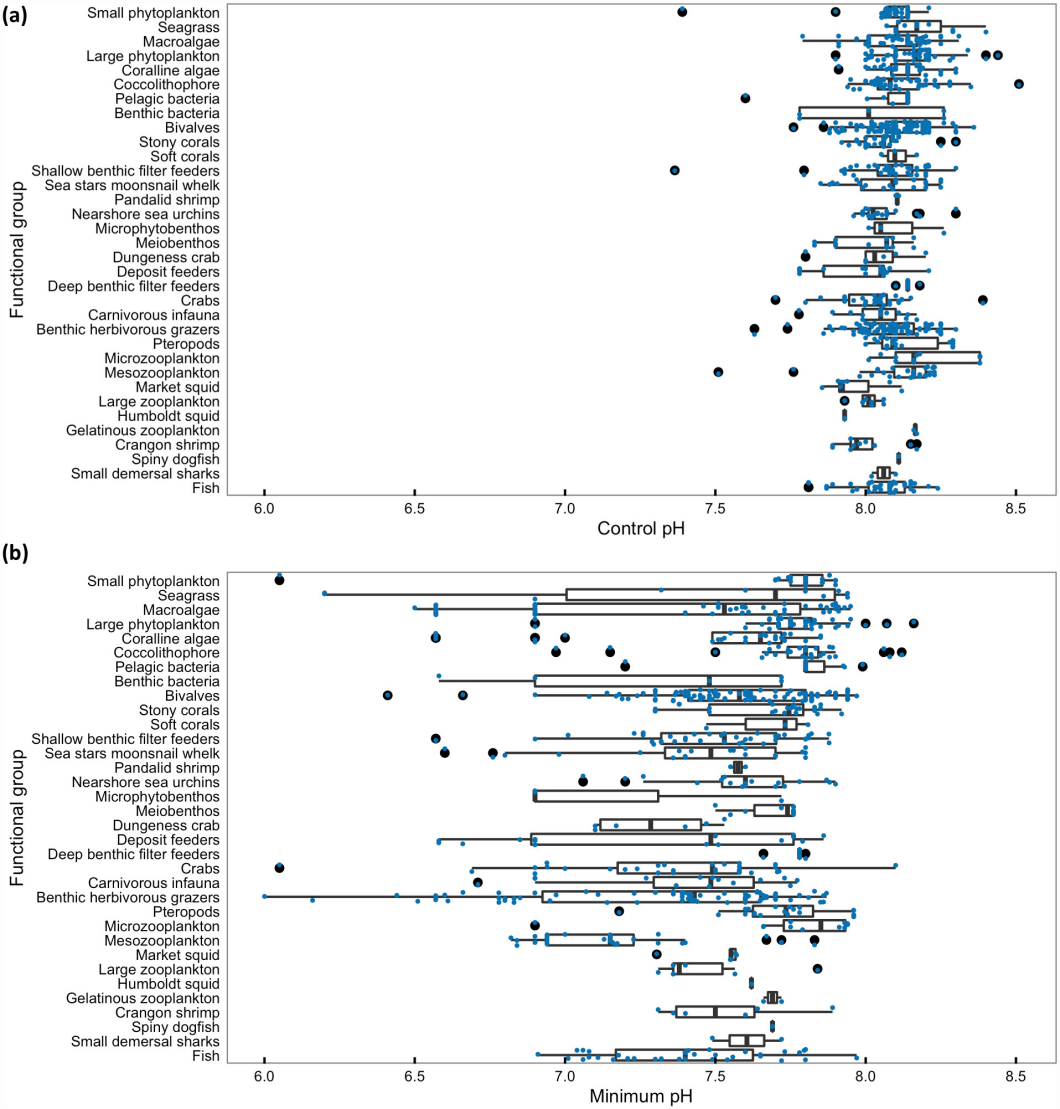
pH conditions used in studies in database. The distribution of (a) control pH and (b) minimum experimental pH for studies included in the database of literature on species response to ocean acidification, by functional group. Each study is indicated by a small point, with the line being the median of the distribution, the box the 25–75% quartiles, the whiskers 1.5 times the interquartile range [48], and the large points the outliers.

Control conditions for studies in the database reflect the global average surface pH condition of ~8.1 (mean ± SD = 8.09±0.12, median = 8.10; Fig 4a). Given that current average pH conditions where most species live in the California Current (<100m) is lower than the global average (pH ~8.0 from Marshall et al., in review; see also [32,33,47]), most experiments in the database have control conditions that are higher than appropriate for the California Current. While some acidification treatment conditions used in experiments are too extreme to be relevant to future carbon chemistry conditions in the California Current, most are in a range relevant to informing species response to predicted future pH conditions in the region [33]; mean ± SD = 7.70±0.38, median = 7.76; Fig 4b).

**Fig 5:**
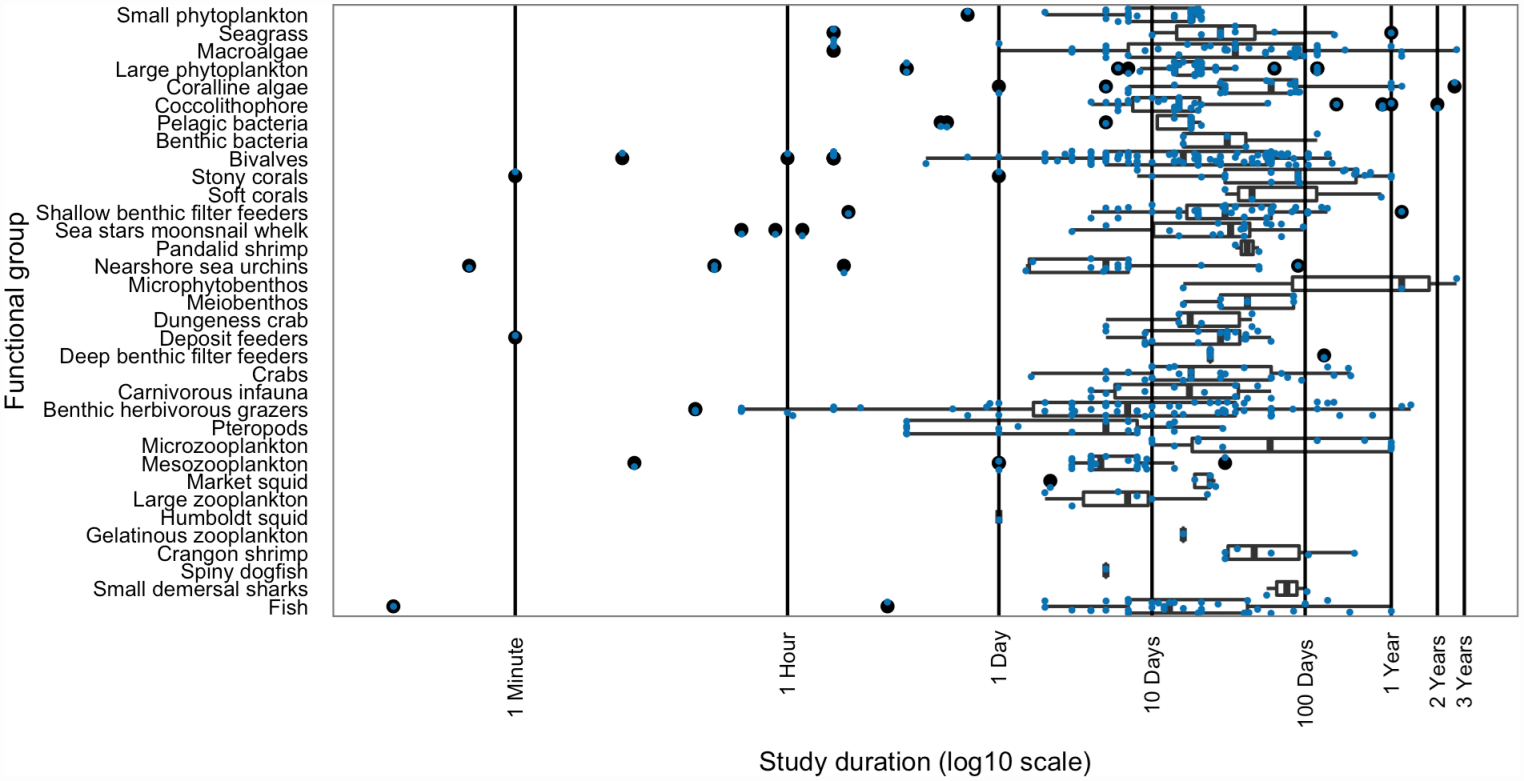
Duration of studies in database. The distribution of study duration for studies included in the database of literature on species response to ocean acidification, by functional group. Each study is indicated by a small point, with the line being the median of the distribution, the box the 25–75% quartiles, the whiskers 1.5 times the interquartile range [48], and the large points the outliers.

Most studies in the database were run for longer than two weeks (median duration = 17 days), with an average study duration of over six weeks (average study duration ± standard deviation = 44 ± 91 days; Fig 5). A handful of studies ran for a year or more.

### Summarizing response to increased CO_2_

**Fig 6:**
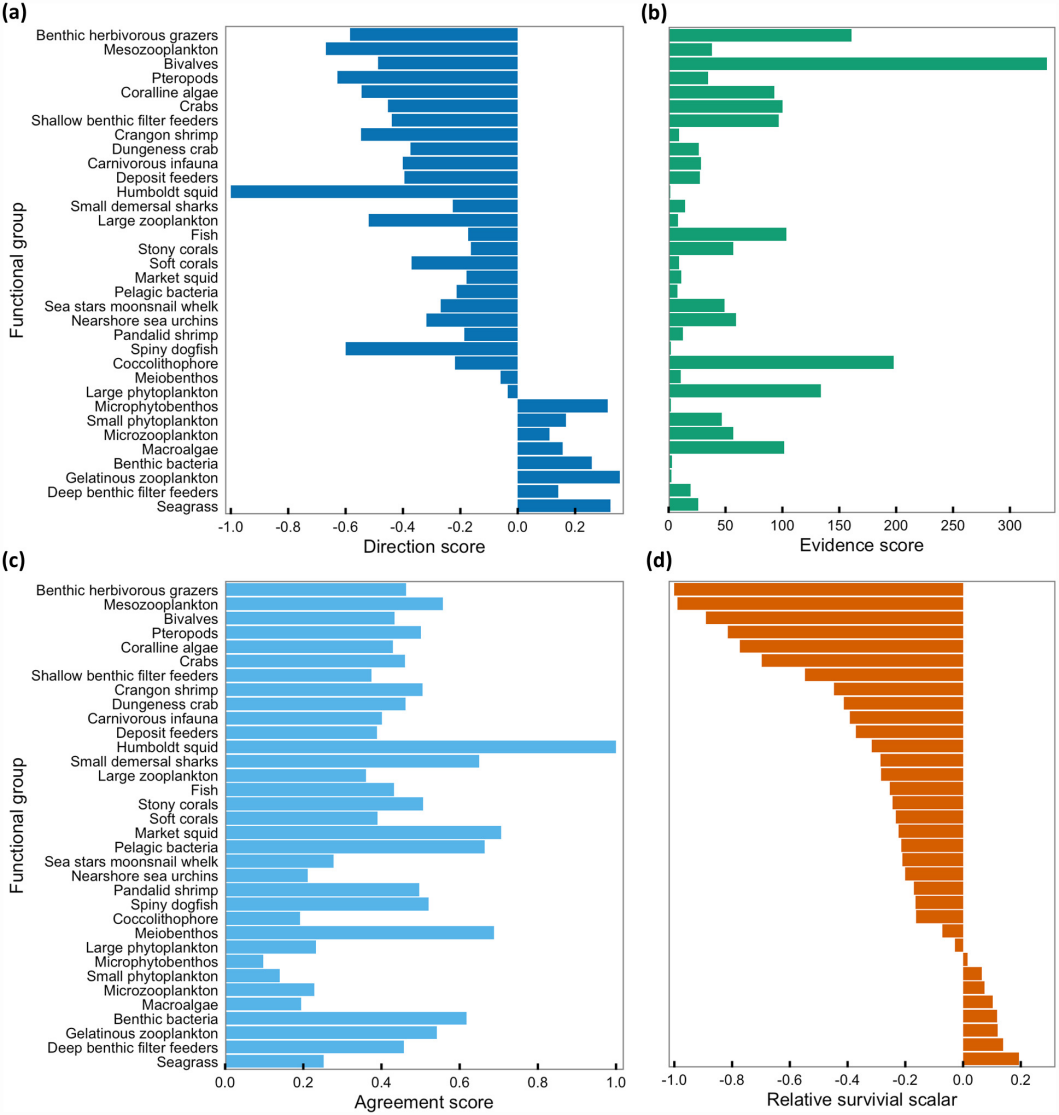
Scores summarizing response to increased CO_2_. (a) Directional, (b) evidence, and (c) agreement scores and (d) relative survival scalar for each functional group, ordered by relative survival scalar values.

Directional scores for the functional groups in the California Current ecosystem model ranged from -1.0 to 0.36 (Fig 6a, S2 Table). Most functional groups had a negative directional score (n = 26), indicating that studies in the database find that increased CO_2_ conditions have generally negative population persistence consequences for these groups. Some functional groups had positive directional scores (microphytobenthos, small phytoplankton, microzooplankton, macroalgae, benthic bacteria, gelatinous zooplankton, deep benthic filter feeders and seagrass), suggesting that studies in the database find that increased CO_2_ conditions have generally positive population persistence consequences for far fewer groups. Those groups with positive scores were a mix of autotrophs and heterotrophs. Three autotroph groups had negative scores (coralline algae, coccolithophores, large phytoplankton). The Humboldt squid functional group had a directional score of -1.0, indicating that all responses in the one study on the species agreed that increased CO_2_ conditions have negative population persistence consequences for the species in laboratory settings.

Evidence scores for the functional groups ranged from 1.55 to 332.67, reflecting a large amount of variation in the number of responses for each functional group and how relevant the responses are to informing survival of California Current species (Fig 6b, S2 Table). For comparison, response number for the functional groups ranged from 2 to 487 (S2 Table). The distribution of evidence scores was not even over its range: nine functional groups had scores less than 10, 13 between 10–50, five between 50–100, six between 100–200, and one with a score over 200. Bivalves and coccolithophores had the highest evidence scores and Humboldt squid and microphytobenthos the lowest evidence scores.

Normalized agreement scores ranged between 0.1 and 1, with four functional groups falling in the upper third of scores (0.66–1) suggesting good agreement among responses, 21 groups in the middle third of scores (0.33–0.65) suggesting little differentiation between no effect responses and directional responses, and nine groups in the lower third of scores (0–0.32) suggesting a relative balance between increase and decrease responses (Fig 6c, S2 Table). Six of the nine responses in the bottom third of the scores (poor agreement in direction of response) were autotrophs. Of the four groups in the upper third of scores (good agreement in direction of response), two were single species squid groups and the others were pelagic bacteria and meiobenthos.

Relative survival scalars ranged from -1.0 to 0.20. The benthic herbivorous grazers functional group had the lowest raw survival scalar and was used as the reference for calculating the relative scalar. By design, the sign of scores mirrored that seen in the directional scores: 26 functional groups had negative relative survival scalars and eight had positive scalars (Fig 6d, S2 Table). The four functional groups with the most negative scalars were: benthic herbivorous grazers, mesozooplankton, bivalves, and pteropods. Those with the most positive scalars were: benthic bacteria, gelatinous zooplankton, deep benthic filter feeders, and seagrass. Confidence scores, a component of the relative survival scalars, ranged between 0.07–2.52, with 12 functional groups below a score of 1, 16 between scores of 1–2, and six between scores of 2–3 (S2 Table). Higher confidence scores indicate greater evidence on species sensitivity to carbonate chemistry and/or agreement among exiting evidence.

### Estimates of pH sensitivity for the California Current ecosystem model

**Fig 7:**
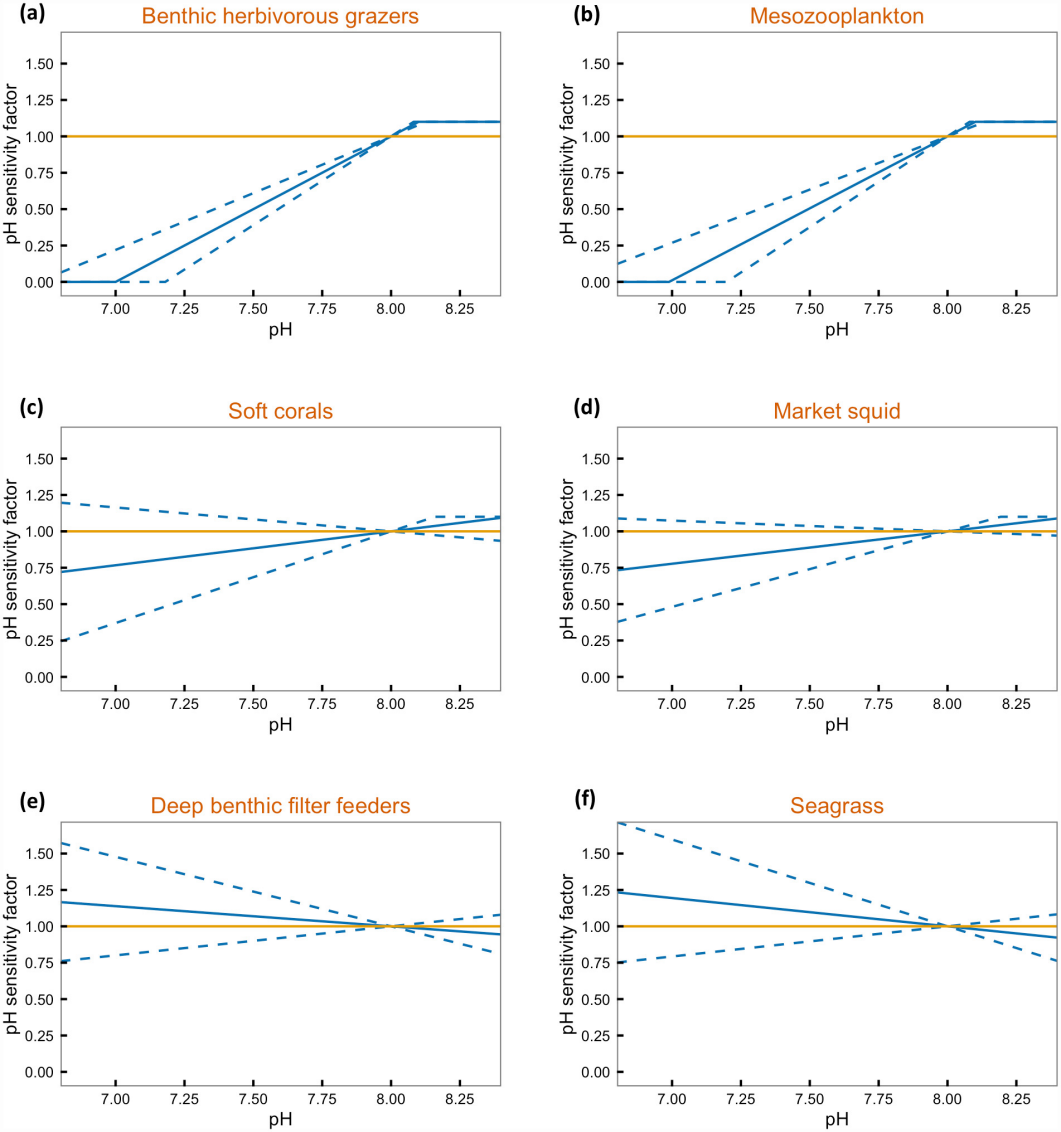
Examples of pH survival sensitivity curves. pH survival sensitivity curves for select functional groups in the California Current ecosystem model, (a,b) functional groups with the most negative response to increased CO_2_, (c,d) functional groups closest to the median sensitivity, and (e,f) functional groups with the most positive response to increased CO_2_.

**Fig 8:**
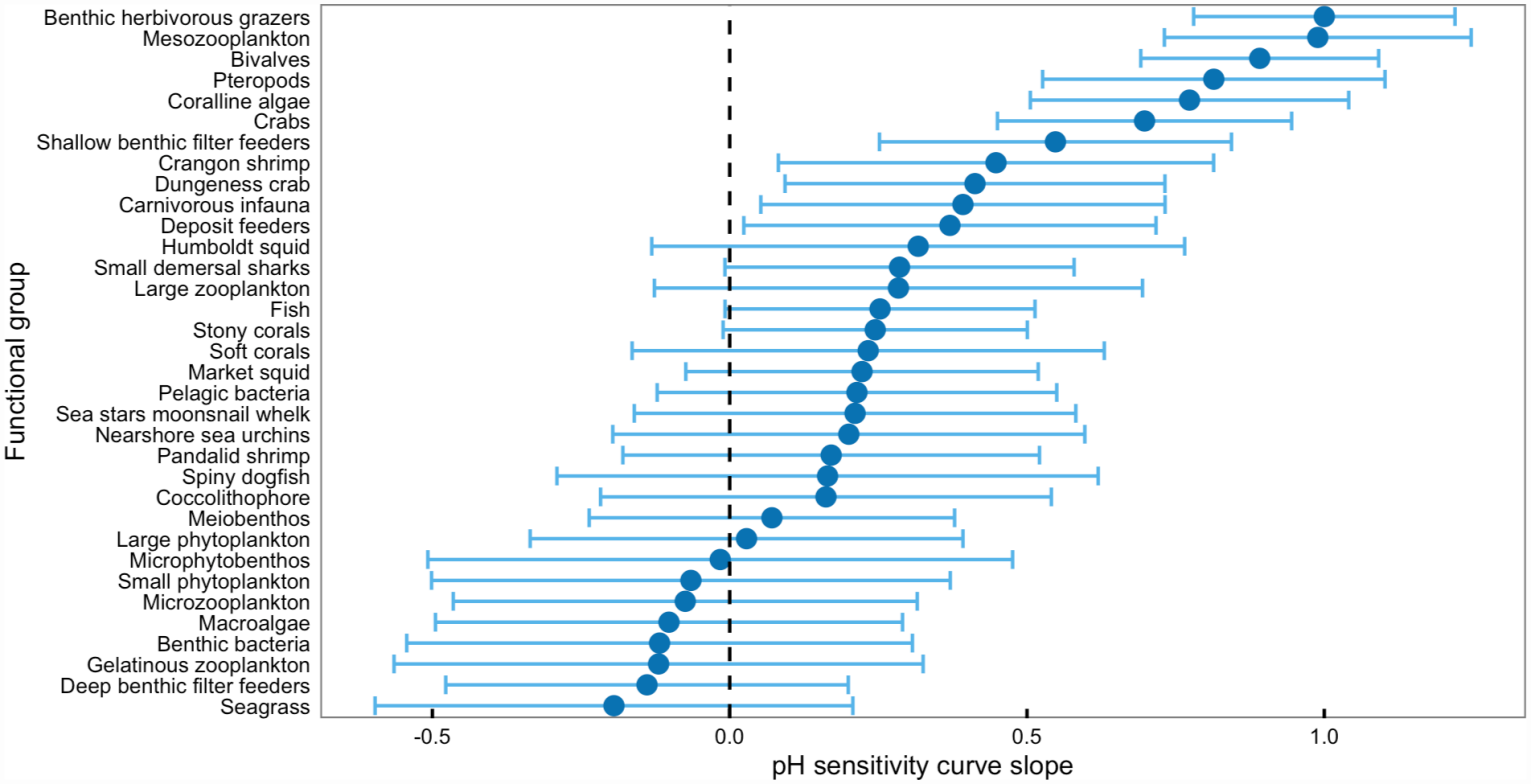
pH survival sensitivity curve slope and error. The slope and slope error for the pH survival sensitivity curves for functional groups in the California Current ecosystem model for which published literature exists on species response to carbonate chemistry conditions.

For 26 functional groups in the California Current ecosystem model, survival declined as pH declined, and, for 8 functional groups, the opposite was true (Figs 7 and 8, S3 Fig). When slope error was applied to and error bands drawn around the pH survival sensitivity curves, the possible slopes for 23 functional groups included a slope of zero (no effect of pH) and the possible slopes for 11 functional groups were entirely above zero (negative effect of pH; Fig 8, S3 Fig).

## Discussion

This study demonstrates a novel method for translating literature on species response to carbonate chemistry conditions into relationships that can be used in ecological modeling scenarios. The method fully embraces uncertainty in species response due to both a lack of knowledge and conflicting results from laboratory studies. By incorporating all available information on temperate species sensitivity to carbonate chemistry conditions and weighting it by its relevance to informing survival of functional groups in the California Current ecosystem, we were able to develop estimates of pH sensitivity tailored to this region, even under data limitation. Our focus on the relevance of studies to the estimates is a distinguishing factor between this work and more traditional syntheses and meta-analyses. The amount of data available to inform the sensitivity of each functional group was highly variable as was the consistency in whether low pH conditions had a positive or negative affect on population persistence. These evidence and agreement scores could be used to inform research priorities for laboratory and field investigations of species sensitivity to carbonate chemistry conditions. As expected given the prevailing tone of literature about ocean acidification, most functional groups (26 of 34) responded negatively to increased CO_2_ conditions (e.g., negative relative survival scalar), including two autotrophic functional groups. However, when uncertainty in sensitivity is considered, only 11 groups had curve distributions that were always negative, suggesting that variation in sensitivity is an important consideration to our overall understanding of the impacts of ocean acidification. We encourage others to use the database and methods presented in this paper in other regional modeling efforts.

### Literature on species sensitivity to carbonate chemistry

The literature related to ocean acidification may be biased against studies finding no response to pH [49]. We have no way to quantify such a potential bias, but the consequence for our analysis would an overestimate of the sensitivity of functional groups to pH. From this study, we were able to identify trends in the literature, some of which may reflect specific biases. The literature on the response of temperate species suggests strong bias towards work on cultivated shellfish species (e.g., oysters, mussels, clams) and on urchins and coccolithophores, groups that have been the subject of extensive laboratory research for other disciplines. This bias is constructive for efforts to help the shellfish industry adapt to ocean acidification [50] and to understand the basic biochemical and physiological processes that define sensitivity to carbonate chemistry conditions (e.g., [26,39,51]). However, the current taxonomic distribution of studies makes it challenging to characterize potential ecosystem response to ocean acidification, for little is known about how ecologically important species in marine food webs, like krill, copepods, squid, forage fish, and the dominant phytoplankton groups, respond. For example, the mesozooplankton group, which is largely copepods, had 59 responses in the published literature, while bivalves had 493 responses. That said, studying the carbonate chemistry sensitivity of many similar species (e.g., urchins) or many populations in a single species can help define the range of responses in a species or species group, providing valuable information about how to generalize results on response to acidification. The literature also shows a bias towards research on adults, rather than early life stages. While adults are often easier to work with in experimental settings, early life stages are likely more sensitive to ocean acidification [52]. For this reason, the literature on species sensitivity to carbonate chemistry conditions may not well represent the response of the most sensitive life stages, which could result in inaccurate characterization of species sensitivity.

Studies in the literature database had varied relevance to survival of California Current species to ocean acidification. We were surprised that almost a third of the species in the database occur in the California Current, and expect that this result reflects the large amount of attention that the issue of ocean acidification has gotten in the region in the scientific and environmental communities (e.g., [53]). However, the treatments considered representative of the present day (e.g., “controls”) use pH conditions that are too high to well represent California Current waters, given that pH in this region is lower than in many other areas of the ocean [24,32,33]. This mismatch between conditions in the California Current and control treatments used in most experiments of species response makes relating the results of these studies to California Current species and ecosystems complex. It is also one of the reasons why we did not apply standard meta-analysis techniques for summarizing species response to treatment (i.e., calculating the effect size of treatment versus control conditions) to develop results for our region of interest. We used a pH range of 7.8–8.1 to represent “average”, control pH levels in the area containing the majority of species (Table 1). Given the high variability of pH in the California Current, future analysis could be improved by defining control conditions at finer spatial and temporal scales and relating pH conditions to habitats used by particular species or functional groups.

The duration of experiments included in the database was longer than we expected. Experiments that run for two to three weeks can capture all or a significant portion of the early life stages of the many marine species included in the database, but represent just a fraction of the adult life stages of the same species, the life stage on which most experiments in the database were conducted. Thus, while experiments that last between two to six weeks (the median and mean of studies in the database) may be able to capture processes of shock and acclimation to increased CO_2_ conditions, especially for physiological processes like acid-base balance and possibly calcification, they likely will not capture the growth and survival response of adults well, except for those species with very short life cycles. That said, results from studies that expose species to increased CO_2_ conditions for a few hours to a couple of weeks do have special relevance to upwelling areas, like the California Current, where upwelling winds can cause low pH waters to invade shallow, near shore areas for time frames of similar duration (e.g., [10,54]). Studies in the database conducted in less than a minute or an hour typically focus on very early processes in the life cycle, such as sperm motility or egg fertilization and cleavage, where such short time frames are likely appropriate for understanding of short-duration physiological processes. Few studies, mostly on phytoplankton, were long enough to capture multigenerational effects.

### Synthesis of species response to carbonate chemistry conditions

**Fig 9:**
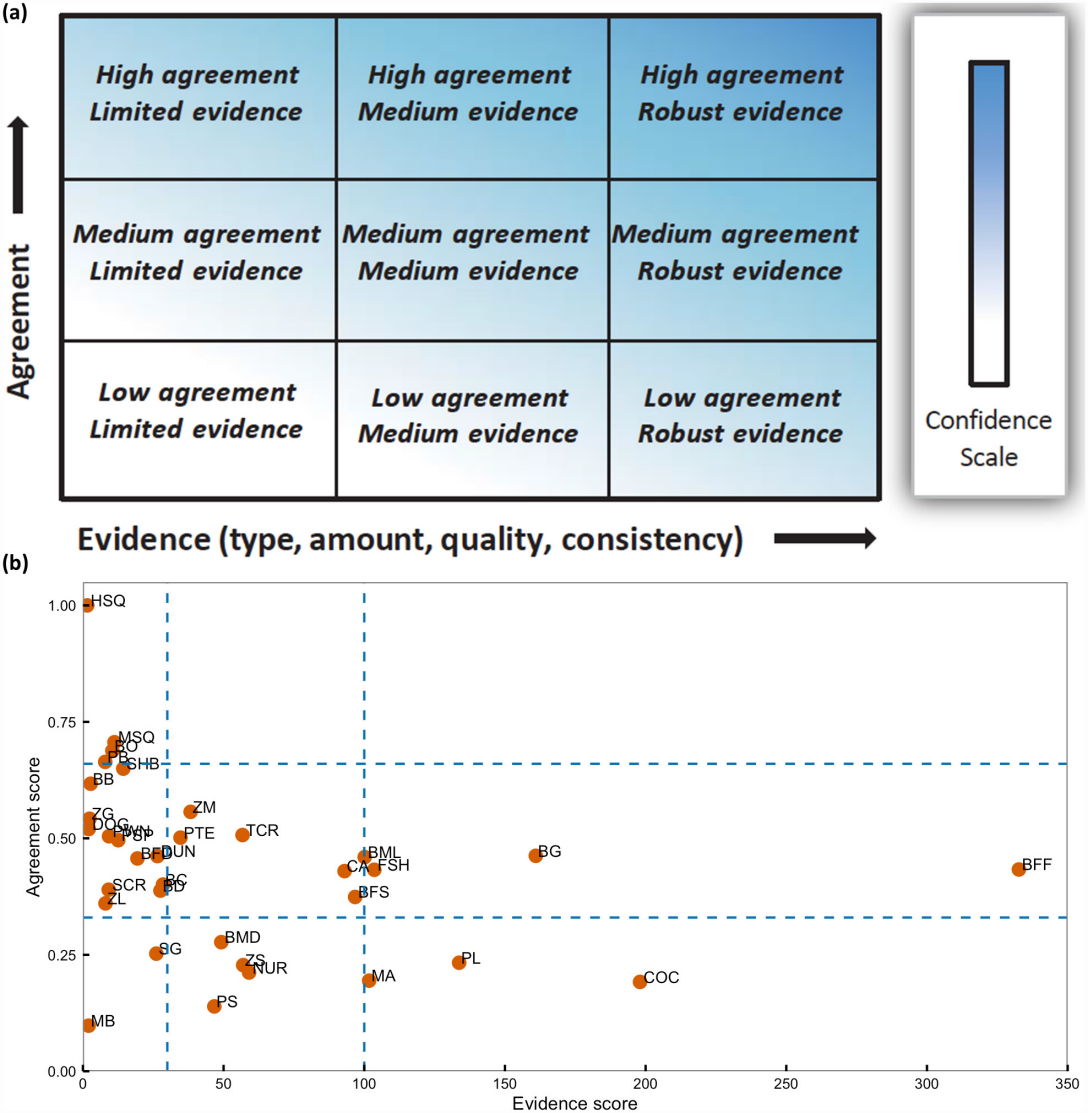
Charactering confidence in scientific findings. (a) The degree of confidence in scientific findings is defined by the Intergovernmental Panel on Climate Change as depending on levels of evidence and agreement and their relationships to increasing confidence. Reprinted from Field et al. [58] under a CC BY license, with permission from Cambridge University Press, original copyright 2014. (b) The evidence and agreement score for each functional group is plotted in a manner similar to (a) with hypothetical bins for levels of evidence and agreement indicated by dotted lines. Each point is labeled with a functional group code: BB = Benthic bacteria, BC = Carnivorous infauna, BD = Deposit feeders, BFD = Deep benthic filter feeders, BFF = Bivalves, BFS = Shallow benthic filter feeders, BG = Benthic herbivorous grazers, BMD = Sea stars moonsnail whelk, BML = Crabs, BO = Meiobenthos, CA = Coralline algae, COC = Coccolithophore, DOG = Spiny dogfish, DUN = Dungeness crab, FSH = Fish, HSQ = Humboldt squid, MA = Macroalgae, MB = Microphytobenthos, MSQ = Market squid, NUR = Nearshore sea urchins, PB = Pelagic bacteria, PL = Large phytoplankton, PS = Small phytoplankton, PSP = Pandalid shrimp, PTE = Pteropods, PWN = Crangon shrimp, SCR = Soft corals, SG = Seagrass, SHB = Small demersal sharks, TCR = Stony corals, ZG = Gelatinous zooplankton, ZL = Large zooplankton, ZM = Mesozooplankton, ZS = Microzooplankton.

Because of the relatively limited amount of information to characterize the response of all functional groups in the California Current to carbonate chemistry conditions, a quantitative approach to assigning sensitivity and response was unrealistic. The qualitative approach used here instead emphasizes the amount of information available on each species group and the direction and consistency of this response. We expect that the information on sensitivity to carbonate chemistry conditions generated from this exercise, when combined with estimates of exposure (i.e., spatial overlap with low pH/high CO_2_ conditions), could be translated into risk assessment exercises [55,56]. A related effort has applied a similar approach to four California Current taxa to better understand their risk to ocean acidification [57]. Because our approach to determining certainty in our understanding of species response to carbonate chemistry conditions mirrors that taken by the IPCC, it should be approachable by non-scientific audiences interested in the impacts of ocean acidification on marine ecosystems. For example, the evidence and agreement scores we assigned to functional groups for this project could be mapped onto the IPCC grid diagram used to display scientific certainty, though the boundaries used to define the limited, moderate, and robust evidence and low, medium, and high agreement would need input from social scientists (Fig 9).

The relative sensitivity ranking of functional groups in this study was somewhat consistent with previous meta-analyses, but differed in important ways. For example, of the 11 functional groups that responded most negativity to decreasing pH (curve distributions that were always negative), nine produce calcium carbonate structures, indicating the potentially high sensitivity of calcifiers. The high sensitivity of calcifiers has been noted in other meta-analyses [13,14], and there is an identifiable mechanism for their response to increased CO_2_ (e.g., [59]). Unlike Wittman and Pörtner [15], we found that four of six crustacean-dominated functional groups (mesozooplankton, crabs, Crangon shrimp, Dungeness crab) were also in this most sensitive group. Most surprising was that the crustacean mesozooplankton group (largely copepods) had the second highest absolute value sensitivity value. The high sensitivity was driven by the second lowest directional score (-0.67) and the fifth highest confidence score (2.03) (S2 Table). The directional score was based on there being twice as many negative rScores as no-effect rScores for the group, and, unlike most functional groups, there were no responses that indicated a positive effect of increased CO_2_. Although we do not have high confidence in the exact rankings in the study (the slope errors overlap for the eight most sensitive species), this result suggests the need for more study on the sensitivity of mesozooplankton. We found fish to have only a moderate sensitivity, counter to the results in Wittman and Pörtner [15], which found fish to have high sensitivity. Most primary producer functional groups were at the bottom of the relative sensitivity ranking, and all but two primary producer groups (coralline algae, coccolithophores) had pH sensitivity curve slopes close to zero or that increased with decreasing pH. This result provides limited support for the potential stimulatory effect of ocean acidification on primary producers ([13,14], but see [60]). The phytoplankton groups all had pH sensitivity values near zero, but with a slight indication of higher survival at low pH for small phytoplankton, a finding generally consistent with Dutkiewicz et al. [61]. Four heterotrophic groups had pH sensitivity curve slopes that increased with decreasing pH (microzooplankton, benthic bacteria, gelatinous zooplankton, and deep benthic filter feeders), suggesting a potential positive impact of ocean acidification, but each had little evidence on which to base this characterization and the mechanisms of this potential enhancement are uncertain. Our analysis did not explicitly consider the severity of measured responses to increased CO_2_, only the direction, which may account for some of the differences between our results and those of other meta-analyses.

We chose to incorporate the confidence score when calculating survival scalars to directly incorporate measurements of certainty into the scalar. An alternative approach would have been to use the directional score as a point estimate and the confidence score to develop bounds around that estimate. We directly incorporated the confidence score so that functional groups with very little data would have a lower survival scalar, tending towards no effect, and groups with ample data could achieve larger absolute survival scalars. For example, Humboldt squid comprise a single-species functional group in the California Current ecosystem model, and there was one study on this species in the database, which has two responses (both negative). The Humboldt squid functional group had the largest absolute directional score (-1.0) of all functional groups, but also the lowest evidence score (1.5). Because we accounted for the low evidence score of this functional group when calculating its survival scalar, about a third of the other functional groups in the model had larger absolute survival scalars than it did. In contrast, the bivalve functional group had a much higher evidence score than any other functional group, but a lower directional score than many other groups because of the many “no effect” results for studies on the species in this group. For this reason, it did not have the highest absolute survival scalar. The group with the highest scalar, benthic herbivorous grazers, had a lower evidence score and a similar agreement score, but a more negative directional score than the bivalve functional group.

The study of species response to carbonate chemistry conditions is a young scientific effort, and scientists are developing the bounds of potential responses [18], some of which are likely to be unexpected (e.g., [62,63]). The exploratory nature of the field leads some scientists to measure a variety of responses during their experiments, which may bias our agreement score, resulting in the large number of functional groups that have agreement scores indicating a balance between no effect responses and directional responses. The balance of “no effect” responses to directional responses might change in the future, but until then, syntheses that characterize species response to ocean acidification should well capture the large number of “no effect” results in the literature.

This project elucidates some of the current difficulties in developing scenarios of species sensitivity to carbonate chemistry conditions for use in ecosystem modeling. Developers of this California Current ecosystem model modified the model to make it more amenable for running scenarios of ocean acidification. For example, the model was altered to accept simple curves of the impacts of pH on functional groups and to split out functional groups for some groups known to be sensitive to carbonate chemistry (e.g., pteropods, coccolithophores; [27]). Even with these changes, challenges remain with the functional groups in the model because most lower-trophic level groups are highly aggregated and taxonomically diverse and there is poor understanding of the proportional biomass of individual species in each group. Thus, it is difficult to know which taxonomic group in the functional group the ocean acidification estimate should resemble most. Even if we did have perfect knowledge on the composition of each functional group in the model, we lack knowledge on the sensitivity to carbonate chemistry conditions of many ecologically important groups, such as gelatinous species and other non-calcifying zoo- and phytoplankton. For many lower-level trophic groups it is challenging to know the direction of response to increased CO_2_ conditions, let alone the magnitude, though this is the target for which scientists are shooting. We encourage experimentalists to choose study subjects for carbonate chemistry sensitivity experiments based, in part, on their role and biomass in the ecosystem, for little is currently known about many functional groups that dominate biomass (e.g., large zooplankton, benthic bacteria; S4 Fig). Conversely, like most food web models, the California Current ecosystem model has high resolution in upper trophic level fish groups. Literature on the response of fish species to carbonate chemistry conditions is limited, and only a handful of fish species from the California Current have been evaluated. Thus, the fish functional groups in the model are more refined than our knowledge on pH response. To deal with this mismatch of information, in this exercise, we developed a pH survival sensitivity curve for all bony fish species together. Finally, most ecological models, including the Atlantis ecosystem model, focus more on adults and post-larval juvenile stage than very early life stages. Explicitly incorporating the dynamics of early life stages in ecosystem models would improve characterizations of the impacts of ocean acidification on ecosystems by allowing the sensitivity of these stages to changing carbonate chemistry to be directly captured in scenarios.

We developed a methodology that could generate species sensitivity relationships for a number of different model parameters, but here created estimates only for the sensitivity of species survival due to limitations in the Atlantis ecosystem model code. For example, scoring systems could be developed to assess the relevance of existing literature to modeling the sensitivity of growth, body composition, or reproductive success to ocean acidification, and we encourage other modelers to do so with the information provided in this paper. In addition, as more data on species sensitivity to carbonate chemistry becomes available, it would be appropriate to modify the method for assigning relevance to each of the responses. For example, future analyses could consider whether the study was pseudo-replicated [64] or could apply a different response score to various response data (e.g., calcification, gene expression) once its relationship to the parameter of interest (e.g., survival, growth) is clarified (Table 1). The approach we used to translate the relative survival scalars into pH survival sensitivity curves with errors is one of many possible options. For example, the reference relationship could be developed using a carbonate chemistry parameter other than pH (e.g., aragonite saturation state) or drawn with shapes other than the hockey stick, and uncertainty in the curve could be captured with techniques other than the slope error estimation. We caution that although the sensitivity curves may appear precise, we do not consider them so, and place more weight in the relative sensitivity of functional groups than the sensitivity curve of a single functional group.

### Translating results

Translating results from short-term laboratory studies to projections of future population processes or dynamics is a difficult exercise, and the results of such studies are rarely correct [20]. While this disconnect is a reality for ocean acidification research, there is a demand for whole ecosystem understanding of the impacts of ocean acidification to inform management and policy options. Output from ecological modeling exercises, while lacking precision and accuracy, is still more robust than output from qualitative, conceptual models that are made without the assistance of vast databases of ecological and environmental information.

Our analysis focused exclusively on sensitivity to carbonate chemistry. To develop realistic predictions of ecosystem response to future conditions, one must consider the entire suite of changing environmental parameters, particularly climate-change driven shifts in temperature and dissolved oxygen. These parameters often have greater effects on survival than pH [49] and strongly co-vary with pH [65]. A critical question not addressed by our analysis (and one for which there are only limited data to answer) is whether pH interacts in non-additive ways with these other potential environmental stressors to affect survival.

Considerable care is needed when designing scenarios of species response to changing environmental conditions, for output from projection efforts is highly dependent on them. The methods for developing estimates of species sensitivity to carbonate chemistry presented in this paper incorporate all of the currently available information on species sensitivity from laboratory and field studies, weight the information by relevance to a single ecosystem, and incorporate information about the amount of and agreement in available evidence. As such, the estimates developed inherently account for certainty in our current understanding of species sensitivity.

## Supporting Information

S1 Database Microsoft Access database of literature on species response to ocean acidification. (ACCDB)

S2 Database References for the manuscripts included in the database of literature on species response to ocean acidification. (PDF)

S1 Fig Diagram of relation among component scores used to calculate pH survival scalars. (TIF)

S2 Fig Number of studies for each functional group with information on species distribution. Number of studies for each functional group with information on whether the species studied is distributed in the California Current (CC) and study subjects are collected from the California Current.
(TIF)

S3 Fig pH survival sensitivity curves. pH survival sensitivity curves for all functional groups in the California Current ecosystem model and some functional groups not in the model (coralline algae, fish) for which published literature exists on species response to carbonate chemistry conditions.
(PDF)

S4 Fig Biomass and evidence score for each functional group. The (a) biomass and (b) evidence score for each functional group in the California Current ecosystem model. Biomass is on a log10 scale.
(TIF)

S1 File PRISMA checklist. (DOC)

S2 File PRISMA flowchart. (DOC)

S1 Table Functional groups in California Current ecosystem model. Invertebrate and vertebrate groups in the California Current ecosystem model that could be directly sensitive to ocean acidification. From Kaplan et al. [26].
(XLSX)

S2 Table Data and scores summarizing functional group response to increased CO_2_ and survival response curve data. Data for each functional group used to develop all scores, the scores themselves, and the OA survival response curve with slope error.
(XLSX)

## References

[1] Doney SC, Fabry VJ, Feely RA, Kleypas JA (2009) Ocean acidification: the other CO_2_ problem. Annual Review of Marine Science 2009.1: 169–192. 10.1146/annurev.marine.010908.163834 21141034

[2] Feely RA, Sabine CL, Lee K, Berelson W, Kleypas J, Fabry VJ, et al (2004) Impact of anthropogenic CO_2_ on the CaCO_3_ system in the oceans. Science 305: 362–366. 15256664 10.1126/science.1097329

[3] Sabine CL, Feely RA, Gruber N, Key RM, Lee K, Bullister JL, et al (2004) The oceanic sink for anthropogenic CO_2_. Science 305: 367–371. 15256665 10.1126/science.1097403

[4] Clarkson MO, Kasemann SA, Wood RA, Lenton TM, Daines SJ, Richoz S, et al (2015) Ocean acidification and the Permo-Triassic mass extinction. Science 348: 229–232. 10.1126/science.aaa0193 25859043

[5] Busch DS, Harvey CJ, McElhany P (2013) Potential impacts of ocean acidifcation on the Puget Sound food web. ICES Journal of Marine Science 70: 823–833.

[6] Griffith GP, Fulton EA, Gorton R, Richardson AJ (2012) Predicting interactions among fishing, ocean warming, and ocean acidification in a marine system with whole-ecosystem models. Conservation Biology 26: 1145–1152. 10.1111/j.1523-1739.2012.01937.x 23009091

[7] Hall-Spencer JM, Rodolfo-Metalpa R, Martin S, Ransome E, Fine M, Turner SM, et al (2008) Volcanic carbon dioxide vents show ecosystem effects of ocean acidification. Nature 454: 96–99. 10.1038/nature07051 18536730

[8] Fabricius KE, De'ath G, Noonan S, Uthicke S (2014) Ecological effects of ocean acidification and habitat complexity on reef-associated macroinvertebrate communities. Proceedings of the Royal Society B: Biological Sciences 281. 10.1098/rspb.2013.2479 PMC3866403 24307670

[9] Enochs IC, Manzello DP, Donham EM, Kolodziej G, Okano R, Johnston L, et al (2015) Shift from coral to macroalgae dominance on a volcanically acidified reef. Nature Climate Change 5: 1083–1088.

[10] Barton A, Hales B, Waldbusser GG, Langdon C, Feely RA (2012) The Pacific oyster, *Crassostrea gigas*, shows negative correlation to naturally elevated carbon dioxide levels: Implications for near-term ocean acidification effects. Limnology and Oceanography 57: 698–710.

[11] Bednaršek N, Feely RA, Reum JCP, Peterson B, Menkel J, Alin S, et al (2014) *Limacina helicina* shell dissolution as an indicator of declining habitat suitability due to ocean acidification in the California Current Ecosystem. Proceedings of the Royal Society B: Biological Sciences 281: 20140123 10.1098/rspb.2014.0123 24789895 PMC4024287

[12] Bednaršek N, Tarling GA, Bakker DCE, Fielding S, Jones EM, Venables HJ, et al (2012) Extensive dissolution of live pteropods in the Southern Ocean. Nature Geosciences 5: 881–885.

[13] Kroeker KJ, Kordas RL, Crim R, Hendriks IE, Ramajo L, Singh GS, et al (2013) Impacts of ocean acidification on marine organisms: quantifying sensitivities and interaction with warming. Global Change Biology 19: 1884–1896. 10.1111/gcb.12179 23505245 PMC3664023

[14] Kroeker KJ, Kordas RL, Crim RN, Singh GG (2010) Meta-analysis reveals negative yet variable effects of ocean acidification on marine organisms. Ecology Letters 13: 1419–1434. 10.1111/j.1461-0248.2010.01518.x 20958904

[15] Wittmann AC, Pörtner H-O (2013) Sensitivities of extant animal taxa to ocean acidification. Nature Climate Change 3: 995–1001.

[16] Cooley SR, Jewett EB, Reichert J, Robbins L, Shrestha G, Wieczorek D, et al (2015) Getting ocean acidification on decision makers’ to-do lists: dissecting the process through case studies. Oceanography 28: 198–211.

[17] Ainsworth CH, Samhouri JF, Busch DS, Chueng WWL, Dunne J, Okey TA (2011) Potential impacts of climate change on Northeast Pacific marine fisheries and food webs. ICES Journal of Marine Science 68: 1217–1229.

[18] Busch DS, O’Donnell MJ, Hauri C, Mach KJ, Poach M, Doney SC, et al (2015) Understanding, characterizing, and communicating responses to ocean acidification: challenges and uncertainties. Oceanography 28: 30–39.

[19] Andersson AJ, Kline DI, Edmunds PJ, Archer SD, Bednarsek N, Carpenter RC, et al (2015) Understanding ocean acidification impacts on organismal to ecological scales. Oceanography 28: 16–27.

[20] Schindler DW, Mills KH, Malley DF, Findlay DL, Shearer JA, Davies IJ, et al (1985) Long-term ecosystem stress: the effects of years of experimental acidification on a small lake. Science 228: 1395–1401. 17814473 10.1126/science.228.4706.1395

[21] Lohbeck KT, Riebesell U, Reusch TBH (2012) Adaptive evolution of a key phytoplankton species to ocean acidification. Nature Geosciences 5: 346–351.

[22] Tatters AO, Schnetzer A, Fu F, Lie AYA, Caron DA, Hutchins DA (2013) Short- versus long-term responses to changing CO_2_ in a coastal dinoflagellate bloom: implications for interspecific competitive interactions and community structure. Evolution 67: 1879–1891. 10.1111/evo.12029 23815646

[23] Thor P, Dupont S (2015) Transgenerational effects alleviate severe fecundity loss during ocean acidification in a ubiquitous planktonic copepod. Global Change Biology 21: 2261–2271. 10.1111/gcb.12815 25430823

[24] McElhany P, Busch DS (2013) Appropriate pCO_2_ treatments in ocean acidification experiments. Marine Biology 160: 1807–1812.

[25] Haigh R, Ianson D, Holt CA, Neate HE, Edwards AM (2015) Effects of ocean acidification on temperate coastal marine ecosystems and fisheries in the Northeast Pacific. PLOS ONE 10: e0117533 10.1371/journal.pone.0117533 25671596 PMC4324998

[26] Waldbusser GG, Hales B, Langdon CJ, Haley BA, Schrader P, Brunner EL, et al (2015) Ocean acidification has multiple modes of action on bivalve larvae. PLOS ONE 10: e0128376 10.1371/journal.pone.0128376 26061095 PMC4465621

[27] Kaplan IC, Marshall KN, Hodgson E, Koehn L (2014) Update for 2014 methodology review: ongoing revisions to the spatially explicit Atlantis ecosystem model of the California Current. NOAA Northwest Fisheries Science Center and University of Washington.

[28] Brand EJ, Kaplan IC, Harvey CJ, Levin PS, Fulton EA, Hermann AJ, et al (2007) A spatially explicit ecosystem model of the California Current’s food web and oceanography. NOAA Technical Memorandum NMFS-NWFSC-84 145 p.

[29] Horne P, Kaplan IC, Marshall K, Levin PS, Harvey CJ, Hermann AJ, et al (2010) Design and parameterization of a spatially explicity ecosystem model of the central California Current. NOAA Technical Memorandum NMFS-NWFSC-1 04 140 p.

[30] Kaplan IC, Horne PJ, Levin PS (2012) Screening California Current fishery management scenarios using the Atlantis end-to-end ecosystem model. Progress in Oceanography 102: 5–18.

[31] Kaplan IC, Levin PS, Burden M, Fulton EA (2010) Fishing catch shares in the face of global change: a framework for integrating cumulative impacts and single species management. Canadian Journal of Fisheries Science 67: 1968–1982.

[32] Feely RA, Sabine CL, Hernandez-Ayon JM, Ianson D, Hales B (2008) Evidence for upwelling of corrosive "acidified" water onto the continental shelf. Science 320: 1490–1492. 10.1126/science.1155676 18497259

[33] Gruber N, Hauri C, Lachkar Z, Loher D, Frölicher TL, Plattner G-K (2012) Rapid progression of ocean acidification in the California Current System. Science 337: 220–223. 10.1126/science.1216773 22700658

[34] National Marine Fisheries Service (2015) Fisheries of the United States, 2014. U.S. Department of Commerce, NOAA Current Fishery Statistics No.2014. Available: https://www.st.nmfs.noaa.gov/commercial-fisheries/fus/fus14/index

[35] Miller JJ, Maher M, Bohaboy E, Friedman CS, McElhany P (2016). Exposure to low pH reduces survival and delays development in early life stages of Dungeness crab (*Cancer magister*). Marine Biology 163: 1–11.

[36] Dupont S, Hall E, Calosi P, Lundve B (2014) First evidence of altered sensory quality in a shellfish exposed to decreased pH relevant to ocean acidification. Journal of Shellfish Research 33:857–861.

[37] Nagelkerken I, Connell SD (2015) Global alteration of ocean ecosystem functioning due to increasing human CO_2_ emissions. Proceedings of the National Academy of Sciences 112: 13272–13277. 10.1073/pnas.1510856112 PMC4629388 26460052

[38] Pespeni MH, Chan F, Menge BA, Palumbi SR (2013) Signs of adaptation to local pH conditions across an environmental mosaic in the California Current Ecosystem. Integrative and Comparative Biology 53: 857–870. 10.1093/icb/ict094 23980118

[39] Meyer J, Riebesell U (2015) Responses of coccolithophores to ocean acidification: a meta-analysis. Biogeosciences 12: 1671–1682.

[40] Miller AW, Reynolds AC, Sobrino C, Riedel GF (2009) Shellfish face uncertain future in high CO_2_ world: influence of acidification on oyster larvae calcification and growth in estuaries. PLOS ONE 4: e5661 10.1371/journal.pone.0005661 19478855 PMC2682561

[41] IPCC (2014) Summary for Policymakers In: *Climate Change 2014*: *Impacts*, *Adaptation*, *and Vulnerability*. *Part A*: *Global and Sectoral Aspects*. *Contribution of Working Group II to the Fifth Assessment Report of the Intergovernmental Panel on Climate Change*. Field CB, Barros VR, Dokken DJ, Mach KJ, Mastrandrea MD, Bilir TE, et al., editors. Cambridge University Press, Cambridge, United Kingdom and New York, NY, USA.

[42] Gattuso J-P, Brewer P, Hoegh-Guldberg O, Kleypas JA, Pörtner H-O, Schmidt D (2014) Ocean acidification: cross-chapter box In: *Climate Change 2014*: *Impacts*, *Adaptation*, *and Vulnerability*. *Part A*: *Global and Sectoral Aspects*. *Contribution of Working Group II to the Fifth Assessment Report of the Intergovernmental Panel on Climate Change*. Field CB, Barros VR, Dokken DJ, Mach KJ, Mastrandrea MD, Bilir TE, et al., editors. Cambridge University Press, Cambridge, United Kingdom and New York, NY, USA.

[43] Hoegh-Guldberg O, Cai R, Brewer PG, Fabry VJ, Hilmi K, Jung S, et al (2014) The Ocean In: *Climate Change 2014*: *Impacts*, *Adaptation*, *and Vulnerability*. *Part A*: *Global and Sectoral Aspects*. *Contribution of Working Group II to the Fifth Assessment Report of the Intergovernmental Panel on Climate Change*. Field CB, Barros VR, Dokken DJ, Mach KJ, Mastrandrea MD, Bilir TE, et al., editors. Cambridge University Press, Cambridge, United Kingdom and New York, NY, USA.

[44] Pörtner H-O, Karl D, Boyd PW, Cheung W, Lluch-Cota SE, Nojiri Y, et al (2014) Ocean systems In: *Climate Change 2014*: *Impacts*, *Adaptation*, *and Vulnerability*. *Part A*: *Global and Sectoral Aspects*. *Contribution of Working Group II to the Fifth Assessment Report of the Intergovernmental Panel on Climate Change*. Field CB, Barros VR, Dokken DJ, Mach KJ, Mastrandrea MD, Bilir TE, et al., editors. Cambridge University Press, Cambridge, United Kingdom and New York, NY, USA.

[45] Riebesell U, Fabry VJ, Hansson L, Gattuso J-P, editors (2010) Guide to best practices for ocean acidification research and data reporting. Luxembourg: Publications Office of the European Union 260 p.

[46] Udovydchenkov IA, Duda TF, Doney SC, Lima ID (2010) Modeling deep ocean shipping noise in varying acidity conditions. The Journal of the Acoustical Society of America 128: EL130–EL136. 10.1121/1.3402284 20815429

[47] Hofmann GE, Smith JE, Johnson KS, Send U, Levin LA, Micheli F, et al (2011) High-frequency dynamics of ocean pH: a multi-ecosystem comparison. PLOS ONE 6: e28983 10.1371/journal.pone.0028983 22205986 PMC3242773

[48] Tukey JW (1977) Exploratory data analysis: Pearson.

[49] Browman HI (2016) Applying organized scepticisim to ocean acidification research. ICES Journal of Marine Science 73: 529–536.

[50] Barton A, Waldbusser GG, Feely RA, Weisberg SB, Newton JA, Hales B, et al (2015) Impacts of coastal acidification on the Pacific Northwest shellfish industry and adaptation strategies implemented in response. Oceanography 28: 146–159.

[51] Padilla-Gamiño JL, Kelly MW, Evans TG, Hofmann GE (2013) Temperature and CO_2_ additively regulate physiology, morphology and genomic responses of larval sea urchins, *Strongylocentrotus purpuratus*. Proceedings of the Royal Society B: Biological Sciences 280: 20130155 10.1098/rspb.2013.0155 23536595 PMC3619508

[52] Ross PM, Parker L, O’Connor WA, Bailey EA (2011) The impact of ocean acidification on reproduction, early development and settlement of marine organisms. Water 3: 1005.

[53] Feely RA, Klinger T, Newton J, Chadsey M (2013) Washington Blue Ribbon Panel on Ocean Acidification: Science white paper. NOAA Oceans and Atmospheric Research Special Report.

[54] Evans W, Hales B, Strutton PG, Shearman RK, Barth JA (2015) Failure to bloom: Intense upwelling results in negligible phytoplankton response and prolonged CO_2_ outgassing over the Oregon shelf. Journal of Geophysical Research: Oceans 120: 1446–1461.

[55] Samhouri JF, Levin PS (2012) Linking land- and sea-based activities to risk in coastal ecosystems. Biological Conservation 145: 118–129.

[56] Hare JA, Morrison WE, Nelson MW, Stachura MM, Teeters EJ, Griffis RB, et al (2016) A vulnerability assessment of fish and invertebrates to climate change on the Northeast U.S. continental shelf. PLOS ONE 11: e0146756 10.1371/journal.pone.0146756 26839967 PMC4739546

[57] Hodgson E, Essington T, Kaplan IC (2014) Appendix management strategy (MS) 2013–05. Assessing the risk of ocean acidification in the California Current to two key fishery species, Dungeness crab (Cancer magister) and pink shrimp (Pandalus jordani) California Current Integrated Ecosystem Assessment Update 2013, U.S. Dept. Commerce, NOAA, Northwest and Southwest Fisheries Science Centers.

[58] Field CB, Barros VR, Mach KJ, Mastrandrea MD, van Aalst M, Adger WN, et al (2014) Technical summary. Ocean acidification: cross-chapter box In: *Climate Change 2014*: *Impacts*, *Adaptation*, *and Vulnerability*. *Summaries*, *Frequently Asked Questions*, *and Cross-Chapter Boxes*. *Contribution of Working Group II to the Fifth Assessment Report of the Intergovernmental Panel on Climate Change*. Field CB, Barros VR, Dokken DJ, Mach KJ, Mastrandrea MD, Bilir TE, et al., editors. Cambridge University Press, Cambridge, United Kingdom and New York, NY, USA.

[59] Pörtner H (2008) Ecosystem effects of ocean acidification in times of ocean warming: a physiologist's view. Marine Ecology Progress Series 373: 203–217.

[60] Gao K, Helbling EW, Häder DP, Hutchins DA (2012) Responses of marine primary producers to interactions between ocean acidification, solar radiation, and warming. Marine Ecology Progress Series 470: 167–189.

[61] Dutkiewicz S, Morris JJ, Follows MJ, Scott J, Levitan O, Dyhrman ST, et al (2015) Impact of ocean acidification on the structure of future phytoplankton communities. Nature Climate Change 5:1002–1006.

[62] Nilsson GE, Dixson DL, Domenici P, McCormick MI, Sorensen C, Watson S-A, et al (2012) Near-future carbon dioxide levels alter fish behaviour by interfering with neurotransmitter function. Nature Climate Change 2: 201–204.

[63] Fu FX, Place AR, Garcia NS, Hutchins DA (2010) CO_2_ and phosphate availability control the toxicity of the harmful bloom dinoflagellate *Karlodinium veneficum*. Aquatic Microbial Ecology 59: 55–65.

[64] Cornwall CE, Hurd CL (In press) Experimental design in ocean acidification research: problems and solutions. ICES Journal of Marine Science.

[65] Reum JCP, Alin SR, Harvey CJ, Bednarsek N, Evans W, Feely RA, et al (2016) Interpretation and design of ocean acidification experiments in upwelling systems in the context of carbonate chemistry co-variation with temperature and oxygen. ICES Journal of Marine Science 73: 582–595.

