# Supplementary material for: Estimates of the Direct Effect of Seawater pH on the Survival Rate of Species Groups in the California Current Ecosystem
Source: PLoS One. 2016 Aug 11;11(8):e0160669. doi: 10.1371/journal.pone.0160669 (PMC4981315; doi:10.1371/journal.pone.0160669)
Supplement: S2 Database — (PDF) [file pone.0160669.s002.pdf]

1. Alexandre A, Silva J, Buapet P, Björk M, Santos R (2012) Effects of CO_2 enrichment on photosynthesis, growth, and nitrogen metabolism of the seagrass *Zostera noltii*. Ecology and Evolution 2: 2625-2635.
2. Algaebase (2014) http://www.algaebase.org/
3. Alsterberg C, Eklöf JS, Gamfeldt L, Havenhand JN, Sundbäck K (2013) Consumers mediate the effects of experimental ocean acidification and warming on primary producers. Proceedings of the National Academy of Sciences 110: 8603-8608.
4. Appelhans YS, Thomsen J, Pansch C, Melzner F, Wahl M (2012) Sour times: seawater acidification effects on growth, feeding behaviour and acid-base status of *Asterias rubens* and *Carcinus maenas*. Marine Ecology Progress Series 459: 85-98.
5. Arnberg M, Calosi P, Spicer JI, Tandberg AHS, Nilsen M, et al. (2013) Elevated temperature elicits greater effects than decreased pH on the development, feeding and metabolism of northern shrimp (*Pandalus borealis*) larvae. Marine Biology 160: 2037-2048.
6. Arnold HE, Kerrison P, Steinke M (2013) Interacting effects of ocean acidification and warming on growth and DMS-production in the haptophyte coccolithophore *Emiliania huxleyi*. Global Change Biology 19: 1007-1016.
7. Arnold KE, Findlay HS, Spicer JI, Daniels CL, Boothroyd D (2009) Effect of CO_2-related acidification on aspects of the larval development of the European lobster, *Hamarus gammarus* (L.). Biogeosciences 6: 1747-1754.
8. Arnold T, Mealey C, Leahey H, Miller AW, Hall-Spencer JM, et al. (2012) Ocean acidification and the loss of phenolic substances in marine plants. PLoS ONE 7: e35107.
9. Asnaghi V, Chiantore M, Mangialajo L, Gazeau F, Francour P, et al. (2013) Cascading effects of ocean acidification in a rocky subtidal community. PLoS ONE 8: e61978.
10. Avgoustidi V, Nightingale PD, Joint I, Steinke M, Turner SM, et al. (2012) Decreased marine dimethyl sulfide production under elevated CO_2 levels in mesocosm and in vitro studies. Environmental Chemistry 9: 399-404.
11. Barros P, Sobral P, Range P, Chícharo L, Matias D (2013) Effects of sea-water acidification on fertilization and larval development of the oyster *Crassostrea gigas*. Journal of Experimental Marine Biology and Ecology 440: 200-206.
12. Barry JP, Buck KR, Lovera C, Brewer PG, Seibel BA, et al. (2013) The response of abyssal organisms to low pH conditions during a series of CO_2-release experiments simulating

deep-sea carbon. Deep-Sea Research Part Ii-Topical Studies in Oceanography 92: 249-260.

13. Barry JP, Lovera C, Buck KR, Peltzer ET, Taylor JR, et al. (2014) Use of a Free Ocean CO_2 Enrichment (FOCE) system to evaluate the effects of ocean acidification on the foraging behavior of a deep-sea urchin. Environmental Science & Technology 48: 9890-9897.
14. Barton A, Hales B, Waldbusser GG, Langdon C, Feely RA (2012) The Pacific oyster, *Crassostrea gigas*, shows negative correlation to naturally elevated carbon dioxide levels: Implications for near-term ocean acidification effects. Limnology and Oceanography 57: 698-710.
15. Baumann H, Talmage SC, Gobler CJ (2012) Reduced early life growth and survival in a fish in direct response to increased carbon dioxide. Nature Climate Change 2: 38-41.
16. Beaugrand G, McQuatters-Gollop A, Edwards M, Goberville E (2013) Long-term responses of North Atlantic calcifying plankton to climate change. Nature Climate Change 3: 263–267.
17. Bechmann RK, Taban IC, Westerlund S, Godal BF, Arnberg M, et al. (2011) Effects of ocean acidification on early life stages of shrimp (*Pandalus borealis*) and mussel (*Mytilus edulis*). Journal of Toxicology and Environmental Health, Part A: Current Issues 74: 424 - 438.
18. Bednaršek N, Feely RA, Reum JCP, Peterson B, Menkel J, et al. (2014) *Limacina helicina* shell dissolution as an indicator of declining habitat suitability owing to ocean acidification in the California Current Ecosystem. Proceedings of the Royal Society B-Biological Sciences 281.
19. Bednaršek N, Tarling GA, Bakker DCE, Fielding S, Cohen A, et al. (2012) Description and quantification of pteropod shell dissolution: a sensitive bioindicator of ocean acidification. Global Change Biology 18: 2378-2388.
20. Bednaršek N, Tarling GA, Bakker DCE, Fielding S, Feely RA (2014) Dissolution dominating calcification process in polar pteropods close to the point of aragonite undersaturation. PLoS ONE 9: e109183.
21. Bednaršek N, Tarling GA, Bakker DCE, Fielding S, Jones EM, et al. (2012) Extensive dissolution of live pteropods in the Southern Ocean. Nature Geosciences 5: 881-885.
22. Beesley A, Lowe DM, Pascoe CK, Widdicombe S (2008) Effects of CO_2-induced seawater

acidification on the health of *Mytilus edulis*. Climate Research 37: 215-225.

23. Beman JM, Chow C-E, King AL, Feng Y, Fuhrman JA, et al. (2011) Global declines in oceanic nitrification rates as a consequence of ocean acidification. Proceedings of the National Academy of Sciences 108: 208-213.
24. Beniash E, Ivanina A, Lieb NS, Kurochkin I, Sokolova IM (2010) Elevated level of carbon dioxide affects metabolism and shell formation in oysters *Crassostrea virginica*. Marine Ecology Progress Series 419: 95-108.
25. Benner I, Diner RE, Lefebvre SC, Li D, Komada T, et al. (2013) *Emiliania huxleyi* increases calcification but not expression of calcification-related genes in long-term exposure to elevated temperature and pCO(2). Philosophical Transactions of the Royal Society B-Biological Sciences 368.
26. Berge T, Daugbjerg N, Balling Andersen B, Hansen PJ (2010) Effect of lowered pH on marine phytoplankton growth rates. Marine Ecology Progress Series 416: 79-91.
27. Bignami S, Enochs IC, Manzello DP, Sponaugle S, Cowen RK (2013) Ocean acidification alters the otoliths of a pantropical fish species with implications for sensory function. Proceedings of the National Academy of Sciences 110: 7366-7370.
28. Bignami S, Sponaugle S, Cowen RK (2013) Response to ocean acidification in larvae of a large tropical marine fish, *Rachycentron canadum*. Global Change Biology 19: 996-1006.
29. Bignami S, Sponaugle S, Cowen RK (2014) Effects of ocean acidification on the larvae of a high-value pelagic fisheries species, mahi-mahi *Coryphaena hippurus*. Aquatic Biology 21: 249-260.
30. Boegner D, Bickmeyer U, Koehler A (2014) CO_2-induced fertilization impairment in *Strongylocentrotus droebachiensis* collected in the Arctic. Helgoland Marine Research 68: 341-356.
31. Bradassi F, Cumani F, Bressan G, Dupont S (2013) Early reproductive stages in the crustose coralline alga *Phymatolithon lenormandii* are strongly affected by mild ocean acidification. Marine Biology: 1-9.
32. Bradbury A, Sizemore B, Rothaus D, Ulrich M (2000) Stock assessment of subtidal geoduck clams (*Panopea abrupta*) in Washington. Olympia, WA: Washington Department of Fish and Wildlife. 68 p.
33. Brading P, Warner ME, Davey P, Smith DJ, Achterberg EP, et al. (2011) Differential effects

of ocean acidification on growth and photosynthesis among phylotypes of Symbiodinium (Dinophyceae). Limnology and Oceanography 56: 927-938.

34. Bramanti L, Movilla J, Guron M, Calvo E, Gori A, et al. (2013) Detrimental effects of ocean acidification on the economically important Mediterranean red coral (*Corallium rubrum*). Global Change Biology 19: 1897-1908.
35. Breitbarth E, Bellerby RJ, Neill CC, Ardelan MV, Meyerhoefer M, et al. (2010) Ocean acidification affects iron speciation during a coastal seawater mesocosm experiment. Biogeosciences 7: 1065-1073.
36. Brennand HS, Soars N, Dworjanyn SA, Davis AR, Byrne M (2010) Impact of ocean warming and ocean acidification on larval development and calcification in the sea urchin *Tripneustes gratilla*. PLoS ONE 5: e11372.
37. Brown MB, Edwards MS, Kim KY (2014) Effects of climate change on the physiology of giant kelp, *Macrocystis pyrifera*, and grazing by purple urchin, *Strongylocentrotus purpuratus*. Algae 29: 203-215.
38. Büdenbender J, Riebesell U, Form A (2011) Calcification of the Arctic coralline red algae *Lithothamnion glaciale* in response to elevated CO_2. Marine Ecology Progress Series 441: 79-87.
39. Burdett HL, Aloisio E, Calosi P, Findlay HS, Widdicombe S, et al. (2012) The effect of chronic and acute low pH on the intracellular DMSP production and epithelial cell morphology of red coralline algae. Marine Biology Research 8: 756-763.
40. Burdett HL, Carruthers M, Donohue PJC, Wicks LC, Hennige SJ, et al. (2014) Effects of high temperature and CO_2 on intracellular DMSP in the cold-water coral *Lophelia pertusa*. Marine Biology 161: 1499-1506.
41. Busch DS, Maher M, Thibodeau P, McElhany P (2014) Shell condition and survival of Puget Sound pteropods are impaired by ocean acidification conditions. PLoS ONE 9: e105884.
42. Byrne M, Gonzalez-Bernat M, Doo S, Foo S, Soars N, et al. (2013) Effects of ocean warming and acidification on embryos and non-calcifying larvae of the invasive sea star *Patiriella regularis*. Marine Ecology Progress Series 473: 235.
43. Byrne M, Ho M, Selvakumaraswamy P, Nguyen HD, Dworjanyn SA, et al. (2009) Temperature, but not pH, compromises sea urchin fertilization and early development under near-future climate change scenarios. Proceedings of the Royal Society

B-Biological Sciences 276: 1883-1888.

44. Byrne M, Ho M, Wong E, Soars NA, Selvakumaraswamy P, et al. (2011) Unshelled abalone and corrupted urchins: development of marine calcifiers in a changing ocean. Proceedings of the Royal Society B: Biological Sciences 278: 2376-2383.
45. Byrne M, Soars N, Ho M, Wong E, McElroy D, et al. (2010) Fertilization in a suite of coastal marine invertebrates from SE Australia is robust to near-future ocean warming and acidification. Marine Biology 157: 2061-2069.
46. Byrne M, Soars N, Selvakumaraswamy P, Dworjanyn SA, Davis AR (2010) Sea urchin fertilization in a warm, acidified and high pCO_2 ocean across a range of sperm densities. Marine Environmental Research 69: 234-239.
47. Caldwell GS, Fitzer S, Gillespie CS, Pickavance G, Turnbull E, et al. (2011) Ocean acidification takes sperm back in time. Invertebrate Reproduction & Development 55: 217-221.
48. Calosi P, Turner LM, Hawkins M, Bertolini C, Nightingale G, et al. (2013) Multiple physiological responses to multiple environmental challenges: an individual approach. Integrative and Comparative Biology 53: 660-670.
49. Carreira C, Heldal M, Bratbak G (2013) Effect of increased pCO_2 on phytoplankton-virus interactions. Biogeochemistry 114: 391-397.
50. Carreiro-Silva M, Cerqueira T, Godinho A, Caetano M, Santos RS, et al. (2014) Molecular mechanisms underlying the physiological responses of the cold-water coral *Desmophyllum dianthus* to ocean acidification. Coral Reefs 33: 465-476.
51. Carter HA, Ceballos-Osuna L, Miller NA, Stillman JH (2013) Impact of ocean acidification on metabolism and energetics during early life stages of the intertidal porcelain crab *Petrolisthes cinctipes*. The Journal of Experimental Biology 216: 1412-1422.
52. Catarino A, Bauwens M, Dubois P (2012) Acid–base balance and metabolic response of the sea urchin *Paracentrotus lividus* to different seawater pH and temperatures. Environmental Science and Pollution Research 19: 2344-2353.
53. Ceballos-Osuna L, Carter HA, Miller NA, Stillman JH (2013) Effects of ocean acidification on early life-history stages of the intertidal porcelain crab *Petrolisthes cinctipes*. The Journal of Experimental Biology 216: 1405-1411.
54. Cerrano C, Cardini U, Bianchelli S, Corinaldesi C, Pusceddu A, et al. (2013) Red coral

extinction risk enhanced by ocean acidification. Scientific Reports 3: 1457.
55. Challener RC, McClintock JB, Makowsky R (2013) Effects of reduced carbonate saturation state on early development in the common edible sea urchin *Lytechinus variegatus*: implications for land-based aquaculture. Journal of Applied Aquaculture 25: 154-175.
56. Chambers RC, Candelmo AC, Habeck EA, Poach ME, Wieczorek D, et al. (2014) Effects of elevated CO_2 in the early life stages of summer flounder, *Paralichthys dentatus*, and potential consequences of ocean acidification. Biogeosciences 11: 1613-1626.
57. Chan KYK, Gruenbaum D, Arnberg M, Thorndyke M, Dupont ST (2013) Ocean acidification induces budding in larval sea urchins. Marine Biology 160: 2129-2135.
58. Chan KYK, Grünbaum D, O'Donnell MJ (2011) Effects of ocean-acidification-induced morphological changes on larval swimming and feeding. The Journal of Experimental Biology 214: 3857-3867.
59. Chan VBS, Li CY, Lane AC, Wang YC, Lu XW, et al. (2012) CO_2-driven ocean acidification alters and weakens integrity of the calcareous tubes produced by the serpulid tubeworm, *Hydroides elegans*. PLoS ONE 7: e42718.
60. Chan VBS, Thiyagarajan V, Lu XW, Zhang T, Shih K (2013) Temperature dependent effects of elevated CO_2 on shell composition and mechanical properties of *Hydroides elegans*: insights from a multiple stressor experiment. PLoS ONE 8: e78945.
61. Chapman RW, Mancia A, Beal M, Veloso A, Rathburn C, et al. (2011) The transcriptomic responses of the eastern oyster, *Crassostrea virginica*, to environmental conditions. Molecular Ecology 20: 1431-1449.
62. Checkley Jr. DM, Dickson AG, Takahashi M, Radich JA, Eisenkolb N, et al. (2009) Elevated CO_2 enhances otolith growth in young fish. Science 324: 1683.
63. Chen S, Beardall J, Gao K (2014) A red tide alga grown under ocean acidification upregulates its tolerance to lower pH by increasing its photophysiological functions. Biogeosciences 11: 4829-4837.
64. Chen S, Gao K (2011) Solar ultraviolet radiation and CO_2-induced ocean acidification interacts to influence the photosynthetic performance of the red tide alga *Phaeocystis globosa* (Prymnesiophyceae). Hydrobiologia 675: 105-117.
65. Cheney DP, Mumford Jr TF (1986) Shellfish and seaweed harvests of Puget Sound. Seattle: Washington Sea Grant.

66. Christensen AB, Nguyen HD, Byrne M (2011) Thermotolerance and the effects of hypercapnia on the metabolic rate of the ophiuroid *Ophionereis schayeri*: Inferences for survivorship in a changing ocean. Journal of Experimental Marine Biology and Ecology 403: 31-38.
67. Christmas A-MF (2013) Effects of ocean acidification on dispersal behavior in the larval stage of the Dungeness crab and the Pacific green short crab. Bellingham, Washington: Western Washington University.
68. Clark D, Lamare M, Barker M (2009) Response of sea urchin pluteus larvae (Echinodermata: Echinoidea) to reduced seawater pH: a comparison among a tropical, temperate, and a polar species. Marine Biology 156: 1125-1137.
69. Collard M, Catarino AI, Bonnet S, Flammang P, Dubois P (2013) Effects of CO_2-induced ocean acidification on physiological and mechanical properties of the starfish *Asterias rubens*. Journal of Experimental Marine Biology and Ecology 446: 355-362.
70. Comeau S, Alliouane S, Gattuso JP (2012) Effects of ocean acidification on overwintering juvenile Arctic pteropods *Limacina helicina*. Marine Ecology Progress Series 456: 279-284.
71. Comeau S, Gorsky G, Alliouane S, Gattuso JP (2010) Larvae of the pteropod *Cavolina inflexa* exposed to aragonite undersaturation are viable but shell-less. Marine Biology Letters 157: 2341-2345.
72. Comeau S, Gorsky G, Jeffree R, Teyssié J-L, Gattuso JP (2009) Impact of ocean acidification on a key Arctic pelagic mollusc (*Limacina helicina*). Biogeosciences 6: 1877-1882.
73. Comeau S, Jeffree R, Teyssié J-L, Gattuso J-P (2010) Response of the arctic pteropod *Limacina helicina* to projected future environmental conditions. PLoS ONE 5: e11362.
74. Connell SD, Russell BD (2010) The direct effects of increasing CO_2 and temperature on non-calcifying organisms: increasing the potential for phase shifts in kelp forests. Proceedings of the Royal Society B: Biological Sciences 277: 1409-1415.
75. Cornwall CE, Hepburn CD, Pritchard D, Currie KI, McGraw CM, et al. (2012) Carbon-use strategies in macroalgae: differential responses to lowered pH and implications for ocean acidification. Journal of Phycology 48: 137-144.
76. Crawfurd KJ, Raven JA, Wheeler GL, Baxter EJ, Joint I (2011) The response of *Thalassiosira pseudonana* to long-term exposure to increased CO_2 and decreased pH.

PLoS ONE 6: e26695.

77. Crim RN, Sunday JM, Harley CDG (2011) Elevated seawater CO_2 concentrations impair larval development and reduce larval survival in endangered northern abalone (*Haliotis kamtschatkana*). Journal of Experimental Marine Biology and Ecology 400: 272-277.
78. Cripps G, Lindeque P, Flynn K (2014) Parental exposure to elevated pCO_2 influences the reproductive success of copepods. Journal of Plankton Research 36: 1165-1174.
79. Cripps G, Lindeque P, Flynn KJ (2014) Have we been underestimating the effects of ocean acidification in zooplankton? Global Change Biology 20: 3377-3385.
80. Dashfield SL, Somerfield PJ, Widdicombe S, Austen MC, Nimmo M (2008) Impacts of ocean acidification and burrowing urchins on within-sediment pH profiles and subtidal nematode communities. Journal of Experimental Marine Biology and Ecology 365: 46-52.
81. Dawson LE (1971) Distribution of benthic infaunal biomass in Puget Sound, Washington and its correlation with environmental parameters. Seattle, WA: University of Washington.
82. De Bodt C, Van Oostende N, Harlay J, Sabbe K, Chou L (2010) Individual and interacting effects of pCO_2 and temperature on *Emiliania huxleyi* calcification: study of the calcite production, the coccolith morphology and the coccosphere size Biogeosciences 7: 1401-1412.
83. Descoteaux R (2014) Effects of ocean acidification on development of Alaskan crab larvae. Fairbanks, AK: University of Alaska, Fairbanks.
84. Diaz-Pulido G, Anthony KRN, Kline DI, Dove S, Hoegh-Guldberg O (2012) Interactions between ocean acidification and warming on the mortality and dissolution of coralline algae. Journal of Phycology 48: 32-39.
85. Dickinson GH, Ivanina AV, Matoo OB, Pörtner HO, Lannig G, et al. (2012) Interactive effects of salinity and elevated CO_2 levels on juvenile eastern oysters, *Crassostrea virginica*. The Journal of Experimental Biology 215: 29-43.
86. Dickinson GH, Matoo OB, Tourek RT, Sokolova IM, Beniash E (2013) Environmental salinity modulates the effects of elevated CO_2 levels on juvenile hard-shell clams, *Mercenaria mercenaria*. Journal of Experimental Biology 216: 2607-2618.
87. Dineshram R, Wong KKW, Xiao S, Yu Z, Qian PY, et al. (2012) Analysis of Pacific oyster larval proteome and its response to high-CO_2. Marine Pollution Bulletin 64: 2160-2167.

88. Dissanayake A, Clough R, Spicer JI, Jones MB (2010) Effects of hypercapnia on acid-base balance and osmo-/iono-regulation in prawns (Decapoda: Palaemonidae). Aquatic Biology 11: 27-36.
89. Dixson DL, Jennings AR, Atema J, Munday PL (2015) Odor tracking in sharks is reduced under future ocean acidification conditions. Global Change Biology 21: 1454-1462.
90. Donohoe CJ (1997) Age, growth, distribution, and food habits of recently settled white seabass, *Atractoscion nobilis*, off San Diego County, California. Fishery Bulletin 95: 709-721.
91. Donohue P, Calosi P, Bates AH, Laverock B, Rastrick S, et al. (2012) Impact of exposure to elevated pCO_2 on the physiology and behaviour of an important ecosystem engineer, the burrowing shrimp *Upogebia deltaura*. Aquatic Biology 15: 73-86.
92. Doo SS, Dworjanyn SA, Foo SA, Soars NA, Byrne M (2012) Impacts of ocean acidification on development of the meroplanktonic larval stage of the sea urchin *Centrostephanus rodgersii*. ICES Journal of Marine Science 69: 460-464.
93. Dorey N, Lancon P, Thorndyke M, Dupont S (2013) Assessing physiological tipping point of sea urchin larvae exposed to a broad range of pH. Global Change Biology 19:3355-3367.
94. Duarte C, Navarro JM, Acuña K, Torres R, Manríquez PH, et al. (2014) Combined effects of temperature and ocean acidification on the juvenile individuals of the mussel *Mytilus chilensis*. Journal of Sea Research 85:308-314.
95. Duckworth AR, Peterson BJ (2013) Effects of seawater temperature and pH on the boring rates of the sponge *Cliona celata* in scallop shells. Marine Biology 160: 27-35.
96. Dupont S, Dorey N, Stumpp M, Melzner F, Thorndyke M (2013) Long-term and trans-life-cycle effects of exposure to ocean acidification in the green sea urchin *Strongylocentrotus droebachiensis*. Marine Biology 160: 1835-1843.
97. Dupont S, Havenhand J, Thorndyke W, Peck L, Thorndyke M (2008) Near-future level of CO_2-driven ocean acidification radically affects larval survival and development in the brittlestar *Ophiothrix fragilis*. Marine Ecology Progress Series 373: 285-294.
98. Dupont S, Lundve B, Thorndyke M (2010) Near future ocean acidification increases growth rate of the lecithotrophic larvae and juveniles of the sea star *Crossaster papposus*. Journal of Experimental Zoology Part B: Molecular and Developmental Evolution 314: 382-389.
99. Dupont S, Moya A, Bailly X (2012) Stable photosymbiotic relationship under CO_2-induced

acidification in the acoel worm *Symsagittifera roscoffensis*. PLoS ONE 7: e29568.

100. Egilsdottir H, Spicer JI, Rundle SD (2009) The effect of CO_2 acidified sea water and reduced salinity of aspects of the embryonic development of the amphipods *Echinogammarus marinus* (Leach). Marine Pollution Bulletin 58: 1187-1191.

101. Eklöf JS, Alsterberg C, Havenhand JN, Sundbäck K, Wood HL, et al. (2012) Experimental climate change weakens the insurance effect of biodiversity. Ecology Letters 15: 864-872.

102. Ellis RP, Bersey J, Rundle SD, Hall-Spencer JM, Spicer JI (2009) Subtle but significant effects of CO_2 acidified seawater on embyros on the intertidal snail, *Littorina obtusata*. Aquatic Biology 5: 41-48.

103. Emerald Diving (2011) http://emeralddiving.com/

104. Encyclopedia of Life (2014) http://eol.org/

105. Engel A, Schulz KG, Riebesell U, Bellerby R, Delille B, et al. (2008) Effects of CO_2 on particle size distribution and phytoplankton abundance during a mesocosm bloom experiment (PeECE II). Biogeosciences 5: 509.

106. Esbaugh AJ, Heuer R, Grosell M (2012) Impacts of ocean acidification on respiratory gas exchange and acid-base balance in a marine teleost, *Opsanus beta*. Journal of Comparative Physiology B-Biochemical Systemic and Environmental Physiology 182: 921-934.

107. Evans TG, Chan F, Menge BA, Hofmann GE (2013) Transcriptomic responses to ocean acidification in larval sea urchins from a naturally variable pH environment. Molecular Ecology 22: 1609-1625.

108. Falkenberg LJ, Connell SD, Russell BD (2013) Disrupting the effects of synergies between stressors: improved water quality dampens the effects of future CO_2 on a marine habitat. Journal of Applied Ecology 50: 51-58.

109. Falkenberg LJ, Russell BD, Connell SD (2012) Stability of strong species interactions resist the synergistic effects of local and global pollution in kelp forests. PLoS ONE 7: e33841.

110. Falkenberg LJ, Russell BD, Connell SD (2013) Contrasting resource limitations of marine primary producers: implications for competitive interactions under enriched CO_2 and nutrient regimes. Oecologia 172: 575-583.

111. Fehsenfeld S, Kiko R, Appelhans Y, Towle D, Zimmer M, et al. (2011) Effects of elevated

seawater pCO_2 on gene expression patterns in the gills of the green crab, *Carcinus maenas*. BMC Genomics 12: 1-17.

112. Fehsenfeld S, Weihrauch D (2013) Differential acid–base regulation in various gills of the green crab *Carcinus maenas*: effects of elevated environmental pCO_2. Comparative Biochemistry and Physiology Part A: Molecular & Integrative Physiology 164: 54-65.
113. Feng Y, Hare C, Leblanc K, Rose J, Zhang Y, et al. (2009) Effects of increased pCO_2 and temperature on the North Atlantic spring bloom. I. The phytoplankton community and biogeochemical response. Marine Ecology Progress Series 388: 13-25.
114. Fernández-Reiriz M, Range P, lvarez-Salgado X, Labarta U (2011) Physiological energetics of juvenile clams *Ruditapes decussatus* in a high CO_2 coastal ocean. Marine Ecology Progress Series 433: 97-105.
115. Fernández-Reiriz MJ, Range P, Álvarez-Salgado XA, Espinosa J, Labarta U (2012) Tolerance of juvenile *Mytilus galloprovincialis* to experimental seawater acidification. Marine Ecology Progress Series 454: 65-74.
116. Ferrari MCO, Dixson DL, Munday PL, McCormick MI, Meekan MG, et al. (2011) Intrageneric variation in antipredator responses of coral reef fishes affected by ocean acidification: implications for climate change projections on marine communities. Global Change Biology 17: 2980-2986.
117. Ferrari MCO, McCormick MI, Munday PL, Meekan MG, Dixson DL, et al. (2011) Putting prey and predator into the CO_2 equation – qualitative and quantitative effects of ocean acidification on predator–prey interactions. Ecology Letters 14: 1143-1148.
118. Findlay HS, Kendall MA, Spicer JI, Widdicombe S (2009) Future high CO_2 in the intertidal may compromise adult barnacle *Semibalanus balanoides* survival and embryonic development rate. Marine Ecology Progress Series 389: 193-202.
119. Findlay HS, Kendall MA, Spicer JI, Widdicombe S (2010) Post-larval development of two intertidal barnacles at elevated CO_2 and temperature Marine Biology 157: 725-735.
120. Findlay HS, Kendall MA, Spicer JI, Widdicombe S (2010) Relative influences of ocean acidification and temperature on intertidal barnacle post-larvae at the northern edge of their geographic distribution. Estuarine, Coastal and Shelf Science 86: 675-682.
121. Findlay HS, Wood HL, Kendall MA, Spicer JI, Twitchett RJ, et al. (2011) Comparing the impact of high CO_2 on calcium carbonate structures in different marine organisms.

Marine Biology Research 7: 565-575.
122. Fiorini S, Middelburg JJ, Gattuso J-P (2011) Testing the effects of elevated pCO_2 on coccolithophores (Prymnesiophycea): comparison between haploid and diploid life stages Journal of Phycology 47: 1281-1291.
123. Fiorini S, Middelburg JJ, Gattuso JP (2011) Effects of elevated CO_2 partial pressure and temperature on the coccolithophore *Syracosphaera pulchra*. Aquatic Microbial Ecology 64: 221-232.
124. Fischer R, Pernet B, Reitner J (2000) Organomineralization of cirratulid annelid tubes - fossil and recent examples. Facies 42: 35-50.
125. Fitzer SC, Caldwell GS, Close AJ, Clare AS, Upstill-Goddard RC, et al. (2012) Ocean acidification induces multi-generational decline in copepod naupliar production with possible conflict for reproductive resource allocation. Journal of Experimental Marine Biology and Ecology 418-419: 30-36.
126. Flimlin G, Beal BF (1993) Major predators of cultured shellfish. North Dartmouth, MA: Northeastern Regional Aquaculture Center, UMass Dartmouth. 6 p.
127. Foo SA, Dworjanyn SA, Poore AGB, Byrne M (2012) Adaptive capacity of the habitat modifying sea urchin *Centrostephanus rodgersii* to ocean warming and ocean acidification: performance of early embryos. PLoS ONE 7: e42497.
128. Form AU, Riebesell U (2012) Acclimation to ocean acidification during long-term CO_2 exposure in the cold-water coral *Lophelia pertusa*. Global Change Biology 18: 843-853.
129. Frieder CA (2014) Present-day nearshore pH differentially depresses fertilization in congeneric sea urchins. Biological Bulletin 226: 1-7.
130. Frieder CA, Gonzalez JP, Bockmon EE, Navarro MO, Levin LA (2014) Can variable pH and low oxygen moderate ocean acidification outcomes for mussel larvae? Global Change Biology 20: 754-764.
131. Frommel A, Schubert A, Piatkowski U, Clemmesen C (2013) Egg and early larval stages of Baltic cod, *Gadus morhua*, are robust to high levels of ocean acidification. Marine Biology 160: 1825-1834.
132. Frommel AY, Maneja R, Lowe D, Malzahn AM, Geffen AJ, et al. (2012) Severe tissue damage in Atlantic cod larvae under increasing ocean acidification. Nature Climate Change 2: 42-46.

133. Frommel AY, Stiebens V, Clemmesen C, Havenhand J (2010) Effect of ocean acidification on marine fish sperm (Baltic cod: *Gadus morhua*). Biogeosciences 7: 3915-3919.
134. Fu F, Tatters A, Hutchins D (2012) Global change and the future of harmful algal blooms in the ocean. Marine Ecology Progress Series 470: 207-233.
135. Fu FX, Place AR, Garcia NS, Hutchins DA (2010) CO_2 and phosphate availability control the toxicity of the harmful bloom dinoflagellate *Karlodinium veneficum*. Aquatic Microbial Ecology 59: 55-65.
136. Fu F-X, Zhang Y, Warner ME, Feng Y, Sun J, et al. (2008) A comparison of future increased CO_2 and temperature effects on sympatric *Heterosigma akashiwo* and *Prorocentrum minimum*. Harmful Algae 7: 76-90.
137. Fujita K, Hikami M, Suzuki A, Kuroyanagi A, Sakai K, et al. (2011) Effects of ocean acidification on calcification of symbiont-bearing reef foraminifers. Biogeosciences 8: 2089-2098.
138. Fukuda S-Y, Suzuki Y, Shiraiwa Y (2014) Difference in physiological responses of growth, photosynthesis and calcification of the coccolithophore *Emiliania huxleyi* to acidification by acid and CO_2 enrichment. Photosynthesis Research 121: 299-309.
139. Gaitan-Espitia JD, Hancock JR, Padilla-Gamino JL, Rivest EB, Blanchette CA, et al. (2014) Interactive effects of elevated temperature and pCO_2 on early-life-history stages of the giant kelp *Macrocystis pyrifera*. Journal of Experimental Marine Biology and Ecology 457: 51-58.
140. Gao K, Xu J, Gao G, Li Y, Hutchins DA, et al. (2012) Rising CO_2 and increased light exposure synergistically reduce marine primary productivity. Nature Climate Change 2: 519-523.
141. Gao K, Zheng Y (2010) Combined effects of ocean acidification and solar UV radiation on photosynthesis, growth, pigmentation and calcification of the coralline alga *Corallina sessilis* (Rhodophyta). Global Change Biology 16: 2388-2398.
142. Gaylord B, Hill TM, Sanford E, Lenz EA, Jacobs LA, et al. (2011) Functional impacts of ocean acidification in an ecologically critical foundation species. The Journal of Experimental Biology 214: 2586-2594.
143. Gazeau F, Gattuso JP, Dawber C, Pronker AE, Peene F, et al. (2010) Effect of ocean acidification on the early life stage of the blue mussel (*Mytilus edulis*). Biogeosciences

Discuss 7: 2927-2947.

144. Gazeau F, Gattuso J-P, Greaves M, Elderfield H, Peene J, et al. (2011) Effect of carbonate chemistry alteration on the early embryonic development of the Pacific oyster (*Crassostrea gigas*). PLoS ONE 6: e23010.

145. Gazeau F, Quiblier C, Jansen JM, Gattuso J-P, Middelberg JJ, et al. (2007) Impact of elevated CO_2 on shellfish calcification. Geophysical Research Letters 34: L07603.

146. Gobler CJ, DePasquale EL, Griffith AW, Baumann H (2014) Hypoxia and acidification have additive and synergistic negative effects on the growth, survival, and metamorphosis of early life stage bivalves. PLoS ONE 9: e83648.

147. Gobler CJ, Talmage SC (2013) Short- and long-term consequences of larval stage exposure to constantly and ephemerally elevated carbon dioxide for marine bivalve populations. Biogeosciences 10: 2241-2253.

148. Gooding RA, Harley CDG, Tang E (2009) Elevated water temperature and carbon dioxide concentration increase the growth of a keystone echinoderm. Proceedings of the Natural Academy of Sciences 106: 9316-9321.

149. Goodwin CL, Pease B (1989) Pacific geoduck clam. US Fish and Wildlife Service Biological Report. 23 p.

150. Gotshall DW (2005) Guide to marine invertebrates — Alaska to Baja California. Monterey, California, USA: Sea Challengers.

151. Gradoville MR, White AE, Letelier RM (2014) Physiological response of *Crocosphaera watsonii* to enhanced and fluctuating carbon dioxide conditions. PLoS ONE 9: e110660.

152. Green L, Jutfelt F (2014) Elevated carbon dioxide alters the plasma composition and behaviour of a shark. Biology Letters 10: 20140538

153. Gustafson RG, Lenarz WH, McCain BB, Schmitt CC, Grant WS, et al. (2000) Status review of Pacific hake, Pacific cod, and walleye pollock from Puget Sound, Washington.

154. Hale R, Calosi P, McNeill L, Mieszkowska N, Widdicombe S (2011) Predicted levels of future ocean acidification and temperature rise could alter community structure and biodiversity in marine benthic communities. Oikos 120: 661-674.

155. Hall-Spencer JM, Rodolfo-Metalpa R, Martin S, Ransome E, Fine M, et al. (2008) Volcanic carbon dioxide vents show ecosystem effects of ocean acidification. Nature 454: 96-99.

156. Hama T, Kawashima S, Shimotori K, Satoh Y, Omori Y, et al. (2012) Effect of ocean

acidification on coastal phytoplankton composition and accompanying organic nitrogen production. Journal of Oceanography 68: 183-194.

157. Hamilton TJ, Holcombe A, Tresguerres M (2014) CO_2-induced ocean acidification increases anxiety in Rockfish via alteration of GABA(A) receptor functioning. Proceedings of the Royal Society B-Biological Sciences 281.

158. Hammer KM, Pedersen SA (2013) Deep-water prawn *Pandalus borealis* displays a relatively high pH regulatory capacity in response to CO_2-induced acidosis. Marine Ecology Progress Series 492: 139-151.

159. Hammer KM, Pedersen SA, Størseth TR (2012) Elevated seawater levels of CO_2 change the metabolic fingerprint of tissues and hemolymph from the green shore crab *Carcinus maenas*. Comparative Biochemistry and Physiology Part D: Genomics and Proteomics 7: 292-302.

160. Hammond LM, Hofmann GE (2012) Early developmental gene regulation in *Strongylocentrotus purpuratus* embryos in response to elevated CO_2 seawater conditions. The Journal of Experimental Biology 215: 2445-2454.

161. Hans S, Fehsenfeld S, Treberg JR, Weihrauch D (2014) Acid-base regulation in the Dungeness crab (*Metacarcinus magister*). Marine Biology 161: 1179-1193.

162. Harbo RM (1999) Whelks to whales: Coastal marine life of Oregon, Washington, British Columbia and Alaska. Vancouver, B.C.: Harbour Publishing.

163. Harvey CJ, Bartz KK, Davies J, Francis TB, Good TP, et al. (2010) A mass-balance model for evaluating food web structure and community-scale indicators in the central basin of Puget Sound. U.S. Dept. Commerce, NOAA Tech. Memo. NMFS-NWFSC-106. 180 p.

164. Hauton C, Tyrrell T, Williams J (2009) The subtle effects of sea water acidification on the amphipod *Gammarus locusta*. Biogeosciences 6: 1479-1489.

165. Havenhand J, Buttler F-R, Thorndyke MC, Williamson JE (2008) Near-future levels of ocean acidification reduce fertilization success in a sea urchin. Current Biology 18: 651-652.

166. Havenhand J, Schlegel O (2009) Near-future levels of ocean acidification do not affect sperm motility and fertilization kinetics in the oyster *Crassostrea gigas* Biogeosciences 6: 3009-3015.

167. Hennige SJ, Wicks LC, Kamenos NA, Bakker DCE, Findlay HS, et al. (2014) Short-term

metabolic and growth responses of the cold-water coral *Lophelia pertusa* to ocean acidification. Deep Sea Research Part II: Topical Studies in Oceanography 99: 27-35.

168. Hettinger A, Sanford E, Hill TM, Hosfelt JD, Russell AD, et al. (2013) The influence of food supply on the response of Olympia oyster larvae to ocean acidification. Biogeosciences Discuss 10: 5781-5802.
169. Hettinger A, Sanford E, Hill TM, Lenz EA, Russell AD, et al. (2013) Larval carry-over effects from ocean acidification persist in the natural environment. Global Change Biology 19: 3317-3326.
170. Hettinger A, Sanford E, Hill TM, Russell AD, Sato KNS, et al. (2012) Persistent carry-over effects of planktonic exposure to ocean acidification in the Olympia oyster. Ecology 93: 2758-2768.
171. Heuer RM, Esbaugh AJ, Grosell M (2012) Ocean acidification leads to counterproductive intestinal base loss in the Gulf Toadfish (*Opsanus beta*). Physiological and Biochemical Zoology 85: 450-459.
172. Hikami M, Ushie H, Irie T, Fujita K, Kuroyanagi A, et al. (2011) Contrasting calcification responses to ocean acidification between two reef foraminifers harboring different algal symbionts. Geophysical Research Letters 38: L19601.
173. Hofmann LC, Straub S, Bischof K (2012) Competition between calcifying and noncalcifying temperate marine macroalgae under elevated CO_2 levels. Marine Ecology Progress Series 464: 89-105.
174. Hofmann LC, Straub S, Bischof K (2013) Elevated CO_2 levels affect the activity of nitrate reductase and carbonic anhydrase in the calcifying rhodophyte *Corallina officinalis*. Journal of Experimental Botany 64: 899-908.
175. Hofmann LC, Yildiz G, Hanelt D, Bischof K (2012) Physiological responses of the calcifying rhodophyte, *Corallina officinalis* (L.), to future CO_2 levels. Marine Biology 159: 783-792.
176. Holcomb M, Cohen A, McCorkle D (2012) An investigation of the calcification response of the scleractinian coral *Astrangia poculata* to elevated pCO_2 and the effects of nutrients, zooxanthellae and gender. Biogeosciences 9: 29-39.
177. Holcomb M, McCorkle DC, Cohen AL (2010) Long-term effects of nutrient and CO_2 enrichment on the temperate coral *Astrangia poculata* (Ellis and Solander, 1786). Journal

of Experimental Marine Biology and Ecology 386: 27-33.

178. Hoppe CJM, Langer G, Rost B (2011) *Emiliania huxleyi* shows identical responses to elevated *p*CO_2 in TA and DIC manipulations. Journal of Experimental Marine Biology and Ecology 406: 54-62.

179. Hurst TP, Fernandez ER, Mathis JT (2013) Effects of ocean acidification on hatch size and larval growth of walleye pollock (*Theragra chalcogramma*). ICES Journal of Marine Science 70: 812-822.

180. Hurst TP, Fernandez ER, Mathis JT, Miller JA, Stinson CM, et al. (2013) Resiliency of juvenile walleye pollock to projected levels of ocean acidification. Aquatic Biology 17: 247-259.

181. Hutchins DA, Fu F-X, Webb EA, Walworth N, Tagliabue A (2013) Taxon-specific response of marine nitrogen fixers to elevated carbon dioxide concentrations. Nature Geosciences 6: 790-795.

182. Iglesias-Rodriguez MD, Halloran PR, Rickaby REM, Hall IR, Colmenero-Hidalgo E, et al. (2008) Phytoplankton calcification in a high-CO_2 world. Science 320: 336-340.

183. Inoue S, Kayanne H, Yamamoto S, Kurihara H (2013) Spatial community shift from hard to soft corals in acidified water. Nature Climate Change 3: 683-687.

184. Intertidal Marine Invertebrates of the South Puget Sound (2010) http://www.nwmarinelife.com

185. Ivanina AV, Dickinson GH, Matoo OB, Bagwe R, Dickinson A, et al. (2013) Interactive effects of elevated temperature and CO_2 levels on energy metabolism and biomineralization of marine bivalves *Crassostrea virginica* and *Mercenaria mercenaria*. Comparative Biochemistry and Physiology a-Molecular & Integrative Physiology 166: 101-111.

186. Jansson A, Norkko J, Norkko A (2013) Effects of reduced pH on *Macoma balthica* larvae from a system with naturally fluctuating pH-dynamics. PLoS ONE 8: e68198.

187. Jensen GC (2014) Crabs and Shrimps of the Pacific Coast: A guide to shallow-water decapods from Southeaster Alaska to the Mexican border. Bremerton, WA: Mola Marine. 240 p.

188. Jiang ZJ, Huang XP, Zhang JP (2010) Effects of CO_2 Enrichment on photosynthesis, growth, and biochemical composition of seagrass *Thalassia hemprichii* (Ehrenb.)

Aschers. Journal of Integrative Plant Biology 52: 904-913.
189. Johnson VR, Brownlee C, Rickaby REM, Graziano M, Milazzo M, et al. (2011) Responses of marine benthic microalgae to elevated CO_2. Marine Biology: 1-12.
190. Johnson VR, Russell BD, Fabricius KE, Brownlee C, Hall-Spencer JM (2012) Temperate and tropical brown macroalgae thrive, despite decalcification, along natural CO_2 gradients. Global Change Biology 18: 2792-2803.
191. Jutfelt F, de Souza KB, Vuylsteke A, Sturve J (2013) Behavioural disturbances in a temperate fish exposed to sustained high-CO_2 levels. PLoS ONE 8: e65825.
192. Kamenos NA, Burdett HL, Aloisio E, Findlay HS, Martin S, et al. (2013) Coralline algal structure is more sensitive to rate, rather than the magnitude, of ocean acidification. Global Change Biology 19: 3621-3628.
193. Kaplan MB, Mooney TA, McCorkle DC, Cohen AL (2013) Adverse effects of ocean acidification on early development of squid (*Doryteuthis pealeii*). PLoS ONE 8: e63714.
194. Kawaguchi S, Ishida A, King R, Raymond B, Waller N, et al. (2013) Risk maps for Antarctic krill under projected Southern Ocean acidification. Nature Climate Change 3: 843-847.
195. Kawaguchi S, Kurihara H, King R, Hale L, Berli T, et al. (2010) Will krill fare well under Southern Ocean acidification? Biology Letters.
196. Kelly MW, Padilla-Gamiño JL, Hofmann GE (2013) Natural variation, and the capacity to adapt to ocean acidification in the keystone sea urchin *Strongylocentrotus purpuratus*. Global Change Biology 19: 2536-2546.
197. Keppel EA, Scrosati RA, Courtenay SC (2012) Ocean acidification decreases growth and development in American lobster (*Homarus americanus*) larvae. Journal of Northwest Atlantic Fishery Science 44: 61-66.
198. Kim H, Spivack AJ, Menden-Deuer S (2013) pH alters the swimming behaviors of the raphidophyte *Heterosigma akashiwo*: Implications for bloom formation in an acidified ocean. Harmful Algae 26: 1-11.
199. Kim J-M, Lee K, Yang EJ, Shin K, Noh JH, et al. (2010) Enhanced production of oceanic dimethylsulfide resulting from CO_2-induced grazing activity in a high CO_2 world. Environmental Science & Technology 44: 8140-8143.
200. Kimura RYO, Takami H, Ono T, Onitsuka T, Nojiri Y (2011) Effects of elevated pCO_2 on

the early development of the commercially important gastropod, Ezo abalone *Haliotis discus hannai*. Fisheries Oceanography 20: 357-366.

201. Ko GWK, Chan VBS, Dineshram R, Choi DKS, Li AJ, et al. (2013) Larval and post-larval stages of Pacific oyster (*Crassostrea gigas*) are resistant to elevated CO_2. PLoS ONE 8: e64147.

202. Ko GWK, Dineshram R, Campanati C, Chan VBS, Havenhand J, et al. (2014) Interactive effects of ocean acidification, elevated temperature, and reduced salinity on early-life stages of the Pacific oyster. Environmental Science & Technology 48: 10079-10088.

203. Kottmeier DM, Rokitta SD, Tortell PD, Rost B (2014) Strong shift from $HCO3^-$ to CO_2 uptake in *Emiliania* huxleyi with acidification: new approach unravels acclimation versus short-term pH effects. Photosynthesis Research 121: 265-275.

204. Kroeker KJ, Gambi MC, Micheli F (2013) Community dynamics and ecosystem simplification in a high-CO_2 ocean. Proceedings of the National Academy of Sciences 110: 12721–12726.

205. Kroeker KJ, Gaylord B, Hill TM, Hosfelt JD, Miller SH, et al. (2014) The role of temperature in determining species' vulnerability to ocean acidification: a case study using *Mytilus galloprovincialis*. PLoS ONE 9: e100353.

206. Kroeker KJ, Micheli F, Gambi MC (2013) Ocean acidification causes ecosystem shifts via altered competitive interactions. Nature Climate Change 3: 156-159.

207. Kroeker KJ, Micheli F, Gambi MC, Martz TR (2011) Divergent ecosystem responses within a benthic marine community to ocean acidification. Proceedings of the National Academy of Sciences 108: 14515-14520.

208. Kurihara H (2008) Effects of CO_2-driven ocean acidification on the early developmental stages on invertebrates. Marine Ecology Progress Series 373: 275-284.

209. Kurihara H, Asai T, Kato S, Ishimatsu A (2008) Effects of elevated p CO_2 on early development in the mussel *Mytilus galloprovincialis*. Aquatic Biology 4: 225-233.

210. Kurihara H, Ishimatsu A (2008) Effects of high CO_2 seawater on the copepod (*Acartia tsuensis*) through all life stage and subsequent generations. Marine Pollution Bulletin 56: 1086-1090.

211. Kurihara H, Kato S, Ishimatsu A (2007) Effects of increased seawater p CO_2 on early development of the oyster *Crassostrea gigas*. Aquatic Biology 1: 91-98.

212. Kurihara H, Matsui M, Furukawa H, Hayashi M, Ishimatsu A (2008) Long-term effects of predicted future seawater CO_2 conditions on the survival and growth of the marine shrimp *Palaemon pacificus*. Journal of Experimental Marine Biology and Ecology 367: 41-46.
213. Kurihara H, Shimode S, Shirayama Y (2004) Effects of raised CO_2 concentration on the egg production rate and early development of two marine copepods (*Acartia steueri* and *Acartia erythraea*). Marine Pollution Bulletin 49: 721-727.
214. Kurihara H, Shimode S, Shirayama Y (2004) Sub-lethal effects of elevated concentrations of CO_2 on planktonic copepods and sea urchins. Journal of Oceanography 60: 743-750.
215. Kurihara H, Takano Y, Kurokawa D, Akasaka K (2012) Ocean acidification reduces biomineralization-related gene expression in the sea urchin, *Hemicentrotus pulcherrimus*. Marine Biology 159: 2819-2826.
216. Kurihara H, Yin R, Nishihara G, Soyano K, Ishimatsu A (2013) Effect of ocean acidification on growth, gonad development and physiology of the sea urchin *Hemicentrotus pulcherrimus*. Aquatic Biology 18: 281-292.
217. Kyte MA. Vacant benthic habitats: where have all the sea pens gone?; 2001; Bellevue, Washington.
218. Lacoue-Labarthe T, Réveillac E, Oberhänsli F, Teyssié JL, Jeffree R, et al. (2011) Effects of ocean acidification on trace element accumulation in the early-life stages of squid *Loligo vulgaris*. Aquatic Toxicology 105: 166-176.
219. Laetz C. Marine benthic invertebrate communities near King County's wastewater outfalls; 1998; Seattle, WA. Puget Sound Action Team. pp. 6.
220. Lamb A, Hanby BP (2005) Marine life of the Pacific Northwest. Madeira Park, BC: Harbour.
221. Landes A, Zimmer M (2012) Acidification and warming affect both a calcifying predator and prey, but not their interaction. Marine Ecology Progress Series 450: 1-10.
222. Lane AC, Mukherjee J, Chan VBS, Thiyagarajan V (2013) Decreased pH does not alter metamorphosis but compromises juvenile calcification of the tube worm *Hydroides elegans*. Marine Biology 160: 1983-1993.
223. Lannig G, Eilers S, Pörtner HO, Sokolova IM, Bock C (2010) Impact of ocean acidification on energy metabolism of oyster, *Crassostrea gigas*, changes in metabolic pathways and

thermal response. Marine Drugs 8: 2318-2339.

224. LaVigne M, Hill TM, Sanford E, Gaylord B, Russell AD, et al. (2013) The elemental composition of purple sea urchin (*Strongylocentrotus purpuratus*) calcite and potential effects of pCO_2 during early life stages. Biogeosciences 10: 3465-3477.

225. Law CS, Breitbarth E, Hoffmann LJ, McGraw CM, Langlois RJ, et al. (2012) No stimulation of nitrogen fixation by non-filamentous diazotrophs under elevated CO_2 in the South Pacific. Global Change Biology 18: 3004-3014.

226. Lefebvre SC, Benner I, Stillman JH, Parker AE, Drake MK, et al. (2012) Nitrogen source and pCO_2 synergistically affect carbon allocation, growth and morphology of the coccolithophore *Emiliania huxleyi*: potential implications of ocean acidification for the carbon cycle. Global Change Biology 18: 493-503.

227. Li G, Campbell DA (2013) Rising CO_2 interacts with growth light and growth rate to alter photosystem II photoinactivation of the coastal diatom *Thalassiosira pseudonana*. PLoS ONE 8: e55562.

228. Li J, Jiang Z, Zhang J, Mao Y, Bian D, et al. (2014) The potential of ocean acidification on suppressing larval development in the Pacific oyster *Crassostrea gigas* and blood cockle *Arca inflata* Reeve. Chinese Journal of Oceanology and Limnology 32: 1307-1313.

229. Li J, Jiang Z, Zhang J, Qiu J-W, Du M, et al. (2013) Detrimental effects of reduced seawater pH on the early development of the Pacific abalone. Marine Pollution Bulletin.

230. Li W, Gao K (2012) A marine secondary producer respires and feeds more in a high CO_2 ocean. Marine Pollution Bulletin 64: 699-703.

231. Li W, Gao KS, Beardall J (2012) Interactive effects of ocean acidification and nitrogen-limitation on the diatom *Phaeodactylum tricornutum*. PLoS ONE 7: e51590.

232. Li Y, Gao K, Villafañe VE, Helbling EW (2012) Ocean acidification mediates photosynthetic response to UV radiation and temperature increase in the diatom *Phaeodactylum tricornutum*. Biogeosciences 9: 3931-3942.

233. Lidbury I, Johnson V, Hall-Spencer JM, Munn CB, Cunliffe M (2012) Community-level response of coastal microbial biofilms to ocean acidification in a natural carbon dioxide vent ecosystem. Marine Pollution Bulletin 64: 1063-1066.

234. Lie U (1965) A quantitative study of benthic infauna in Puget Sound, Washington, U.S.A., in 1963-1964. Fiskeridirektoratets Skrifter Serie Havundersøkelser 14: 229-556.

235. Lindh MV, Riemann L, Baltar F, Romero-Oliva C, Salomon PS, et al. (2013) Consequences of increased temperature and acidification on bacterioplankton community composition during a mesocosm spring bloom in the Baltic Sea. Environmental Microbiology Reports 5: 252-262.
236. Lischka S, Budenbender J, Boxhammer T, Riebesell U (2011) Impact of ocean acidification and elevated temperatures on early juveniles of the polar shelled pteropod *Limacina helicina*: mortality, shell degradation, and shell growth. Biogeosciences 8: 919-932.
237. Lischka S, Riebesell U (2012) Synergistic effects of ocean acidification and warming on overwintering pteropods in the Arctic. Global Change Biology 18: 3417-3528.
238. Liu W, He M (2012) Effects of ocean acidification on the metabolic rates of three species of bivalve from southern coast of China. Chinese Journal of Oceanology and Limnology 30: 206-211.
239. Liu W, Huang X, Lin J, He M (2012) Seawater acidification and elevated temperature affect gene expression patterns of the pearl oyster *Pinctada fucata*. PLoS ONE 7: e33679.
240. Liu Y, Xu J, Gao K (2012) CO_2-driven seawater acidification increases photochemical stress in a green alga. Phycologia 51: 562-566.
241. Lohbeck KT, Riebesell U, Collins S, Reusch TBH (2013) Functional genetic divergence in high CO_2 adapted *Emiliania huxleyi* populations. Evolution 67: 1892-1900.
242. Lohbeck KT, Riebesell U, Reusch TBH (2012) Adaptive evolution of a key phytoplankton species to ocean acidification. Nature Geosciences 5: 346-351.
243. Lohbeck KT, Riebesell U, Reusch TBH (2014) Gene expression changes in the coccolithophore *Emiliania huxleyi* after 500 generations of selection to ocean acidification. Proceedings of the Royal Society B-Biological Sciences 281: 20140003.
244. Lombardi C, Cocito S, Gambi MC, Cisterna B, Flach F, et al. (2011) Effects of ocean acidification on growth, organic tissue and protein profile of the Mediterranean bryozoan *Myriapora truncata*. Aquatic Biology 13: 251-262.
245. Lombardi C, Rodolfo-Metalpa R, Cocito S, Gambi MC, Taylor PD (2011) Structural and geochemical alterations in the Mg calcite bryozoan *Myriapora truncata* under elevated seawater pCO_2 simulating ocean acidification. Marine Ecology 32: 211-221.
246. Long CW, Swiney KM, Foy RJ (2013) Effects of ocean acidification on the embryos and larvae of red king crab, *Paralithodes camtschaticus*. Marine Pollution Bulletin 69: 38-47.

247. Long WC, Swiney KM, Harris C, Page HN, Foy RJ (2013) Effects of ocean acidification on juvenile red king crab (*Paralithodes camtschaticus*) and Tanner crab (*Chionoecetes bairdi*) growth, condition, calcification, and survival. PLoS ONE 8: e60959.

248. Maas AE, Wishner KF, Seibel BA (2012) The metabolic response of pteropods to acidification reflects natural CO_2-exposure in oxygen minimum zones. Biogeosciences 9: 747-757.

249. Maier C, Bils F, Weinbauer MG, Watremez P, Peck MA, et al. (2013) Respiration of Mediterranean cold-water corals is not affected by ocean acidification as projected for the end of the century. Biogeosciences 10: 5671-5680.

250. Maier C, Schubert A, Sanchez MMB, Weinbauer MG, Watremez P, et al. (2013) End of the century pCO_2 levels do not impact calcification in Mediterranean cold-water corals. PLoS ONE 8: e62655.

251. Maier C, Watremez P, Taviani M, Weinbauer MG, Gattuso JP (2012) Calcification rates and the effect of ocean acidification on Mediterranean cold-water corals. Proceedings of the Royal Society B: Biological Sciences 279: 1716-1723.

252. Maneja RH, Frommel AY, Browman HI, Clemmesen C, Geffen AJ, et al. (2013) The swimming kinematics of larval Atlantic cod, *Gadus morhua* L., are resilient to elevated seawater pCO_2. Marine Biology 160: 1963-1972.

253. Manno C, Morata N, Primicerio R (2012) *Limacina retroversa*'s response to combined effects of ocean acidification and sea water freshening. Estuarine, Coastal and Shelf Science 113: 163-171.

254. Manríquez PH, Jara ME, Mardones ML, Navarro JM, Torres R, et al. (2013) Ocean acidification disrupts prey responses to predator cues but not net prey shell growth in *Concholepas concholepas* (loco). PLoS ONE 8: e68643.

255. Marchant HK, Calosi P, Spicer JI (2010) Short-term exposure to hypercapnia does not compromise feeding, acid-base balance or respiration of *Patella vulgata* but surprisingly is accompanied by radula damage. Journal of the Marine Biological Association of the United Kingdom 90: 1379-1384.

256. Martin S, Cohu S, Vignot C, Zimmerman G, Gattuso J-P (2013) One-year experiment on the physiological response of the Mediterranean crustose coralline alga, *Lithophyllum cabiochae*, to elevated pCO_2 and temperature. Ecology and Evolution 3: 676-693.

257. Martin S, Gattuso J-P (2009) Response of Mediterranean coralline algae to ocean acidification and elevated temperature. Global Change Biology 15: 2089-2100.

258. Martin S, Richier S, Pedrotti M-L, Dupont S, Castejon C, et al. (2011) Early development and molecular plasticity in the Mediterranean sea urchin *Paracentrotus lividus* exposed to CO_2-driven acidification. Journal of Experimental Biology 214: 1357-1368.

259. Martin S, Rodolfo-Metalpa R, Ransome E, Rowley S, Buia M-C, et al. (2008) Effects of naturally acidified seawater on seagrass calcareous epibionts. Biology Letters 4: 689-692.

260. Matoo OB, Ivanina AV, Ullstad C, Beniash E, Sokolova IM (2013) Interactive effects of elevated temperature and CO_2 levels on metabolism and oxidative stress in two common marine bivalves (*Crassostrea virginica* and *Mercenaria mercenaria*). Comparative Biochemistry and Physiology Part A: Molecular & Integrative Physiology 164: 545-553.

261. Matozzo V, Chinellato A, Munari M, Bressan M, Marin MG (2013) Can the combination of decreased pH and increased temperature values induce oxidative stress in the clam *Chamelea gallina* and the mussel *Mytilus galloprovincialis*? Marine Pollution Bulletin 72: 34-40.

262. Matozzo V, Chinellato A, Munari M, Finos L, Bressan M, et al. (2012) First evidence of immunomodulation in bivalves under seawater acidification and increased temperature. PLoS ONE 7: e33820.

263. Matson PG, Yu PC, Sewell MA, Hofmann GE (2012) Development under elevated pCO_2 conditions does not affect lipid utilization and protein content in early life-history stages of the purple sea urchin, *Strongylocentrotus purpuratus*. The Biological Bulletin 223: 312-327.

264. Matthiessen B, Eggers SL, Krug SA (2012) High nitrate to phosphorus regime attenuates negative effects of rising pCO_2 on total population carbon accumulation Biogeosciences 9: 1195-1203.

265. Mauzey EA (1968) Feeding behavior of Asteroids and escape responses of their prey in the Puget Sound. Ecology 49: 603-619.

266. Mayor DJ, Everett NR, Cook KB (2012) End of century ocean warming and acidification effects on reproductive success in a temperate marine copepod. Journal of Plankton Research 34: 258-262.

267. McCarthy A, Rogers SP, Duffy SJ, Campbell DA (2012) Elevated carbon dioxide

differentially alters the photophysiology of *Thalassiosira pseudonana* (Bacillariophyceae) and *Emiliania huxleyi* (Haptophyta). Journal of Phycology 48: 635-646.

268. McCoy SJ (2013) Morphology of the crustose coralline alga *Pseudolithophyllum muricatum* (Corallinales, Rhodophyta) responds to 30 years of ocean acidification in the Northeast Pacific. Journal of Phycology 49: 830-837.
269. McCoy SJ, Ragazzola F (2014) Skeletal trade-offs in coralline algae in response to ocean acidification. Nature Climate Change 4: 719-723.
270. McDonald MR, McClintock JB, Amsler CD, Rittschof D, Angus RA, et al. (2009) Effects of ocean acidification over the life history of the barnacle *Amphibalanus amphitrite*. Marine Ecology Progress Series 385: 179-187.
271. McElroy DJ, Nguyen HD, Byrne M (2012) Respiratory response of the intertidal seastar *Parvulastra exigua* to contemporary and near-future pulses of warming and hypercapnia. Journal of Experimental Marine Biology and Ecology 416–417: 1-7.
272. McIntyre-Wressnig A, Bernhard JM, Wit JC, McCorkle DC (2014) Ocean acidification not likely to affect the survival and fitness of two temperate benthic formaninferal species: results from culture experiments Journal of Foraminiferal Research 44: 341-351.
273. McLaskey A (In prep) Krill larvae (*Euphausia pacifica*) have decreased survival under ocean acidification conditions.
274. Meakin NG, Wyman M (2011) Rapid shifts in picoeukaryote community structure in response to ocean acidification. ISME Journal 5: 1397-1405.
275. Melatunan S, Calosi P, Rundle S, Widdicombe S, Moody A (2013) Effects of ocean acidification and elevated temperature on shell plasticity and its energetic basis in an intertidal gastropod. Marine Ecology Progress Series 472: 155-168.
276. Melatunan S, Calosi P, Simon DR, Moody AJ, Widdicombe S (2011) Exposure to elevated temperature and pCO reduces respiration rate and energy status in the periwinkle *Littorina littorea*. Physiological and Biochemical Zoology 84: 583-594.
277. Melzner F, Göbel S, Langenbuch M, Gutowska MA, Pörtner H-O, et al. (2009) Swimming performance in Atlantic Cod (*Gadus morhua*) following long-term (4-12 months) acclimation to elevated seawater PCO_2. Aquatic Toxicology 92: 30-37.
278. Melzner F, Stange P, Trübenbach K, Thomsen J, Casties I, et al. (2011) Food supply and

seawater *p*CO_2 impact calcification and internal shell dissolution in the Blue Mussel *Mytilus edulis*. PLoS ONE 6: e24223.

279. Meron D, Rodolfo-Metalpa R, Cunning R, Baker AC, Fine M, et al. (2012) Changes in coral microbial communities in response to a natural pH gradient. ISME Journal 6: 1775-1785.
280. Miles H, Widdicombe S, Spicer JI, Hall-Spencer J (2007) Effects of anthropogenic seawater acidification on acid–base balance in the sea urchin *Psammechinus miliaris*. Marine Pollution Bulletin 54: 89-96.
281. Miller AW, Reynolds AC, Sobrino C, Riedel GF (2009) Shellfish face uncertain future in high CO_2 world: influence of acidification on oyster larvae calcification and growth in estuaries. PLoS One 4: e5661.
282. Miller J (2015) Effect of low pH on early life stages of the decapod crustacean, Dungeness crab (*Cancer magister*). Seattle: University of Washington.
283. Miller SH, Zarate S, Smith EH, Gaylord B, Hosfelt JD, et al. (2014) Effect of elevated pCO(2) on metabolic responses of porcelain crab (*Petrolisthes cinctipes*) larvae exposed to subsequent salinity stress. PLoS ONE: e109167.
284. Mills C (2001) http://faculty.washington.edu/cemills/
285. Morita M, Suwa R, Iguchi A, Nakamura M, Shimada K, et al. (2010) Ocean acidification reduces sperm flagellar motility in broadcast spawning reef invertebrates. Zygote 18: 103-107.
286. Moulin L, Catarino AI, Claessens T, Dubois P (2011) Effects of seawater acidification on early development of the intertidal sea urchin *Paracentrotus lividus* (Lamarck 1816). Marine Pollution Bulletin 62: 48-54.
287. Movilla J, Calvo E, Pelejero C, Coma R, Serrano E, et al. (2012) Calcification reduction and recovery in native and non-native Mediterranean corals in response to ocean acidification. Journal of Experimental Marine Biology and Ecology 438: 144-153.
288. Mueller MN, Beaufort L, Bernard O, Pedrotti ML, Talec A, et al. (2012) Influence of CO_2 and nitrogen limitation on the coccolith volume of *Emiliania huxleyi* (Haptophyta) Biogeosciences 9: 4155-4167.
289. Mukherjee J, Wong KKW, Chandramouli KH, Qian P-Y, Leung PTY, et al. (2013) Proteomic response of marine invertebrate larvae to ocean acidification and hypoxia

during metamorphosis and calcification. Journal of Experimental Biology 216: 4580-4589.

290. Munday PL, Crawley NE, Nilsson GE (2009) Interacting effects of elevated temperature and ocean acidification on the aerobic performance of coral reef fishes. Marine Ecology Progress Series 388: 235-242.

291. Navarro JM, Torres R, Acuña K, Duarte C, Manriquez PH, et al. (2012) Impact of medium-term exposure to elevated pCO_2 levels on the physiological energetics of the mussel *Mytilus chilensis*. Chemosphere 90: 1242-1248.

292. Navarro MO, Bockmon EE, Frieder CA, Gonzalez JP, Levin LA (2014) Environmental pH, O_2 and capsular effects on the geochemical composition of statoliths of embryonic squid *Doryteuthis opalescens*. Water 6: 2233-2254.

293. Newbold LK, Oliver AE, Booth T, Tiwari B, DeSantis T, et al. (2012) The response of marine picoplankton to ocean acidification. Environmental Microbiology 14: 2293-2307.

294. Nguyen HD, Doo SS, Soars NA, Byrne M (2012) Non-calcifying larvae in a changing ocean: warming, not acidification/hypercapnia, is the dominant stressor on development of the sea star *Meridiastra calcar*. Global Change Biology 18: 2466-2476.

295. Nichols FH (1975) Dynamics and energetics of three deposit-feeding benthic invertebrate populations in Puget Sound, Washington. Ecological Monographs 45: 57-82.

296. Nienhuis S, Palmer AR, Harley CDG (2010) Elevated CO_2 affects shell dissolution rate but not calcification rate in a marine snail. Proceedings of the Royal Society B: Biological Sciences 277: 2553-2558.

297. Noisette F, Comtet T, Legrand E, Bordeyne F, Davoult D, et al. (2014) Does encapsulation protect embryos from the effects of ocean acidification? The example of *Crepidula fornicata*. PLoS ONE 9: e93021.

298. Noisette F, Duong G, Six C, Davoult D, Martin S (2013) Effects of elevated pCO_2 on the metabolism of a temperate rhodolith *Lithothamnion corallioides* grown under different temperatures. Journal of Phycology 49: 746-757.

299. O'Clair RM, O'Clair CE (1998) Southeast Alaska's rocky shores: animals. Auke Bay, AK: Plant Press.

300. O'Donnell MJ, George MN, Carrington E (2013) Mussel byssus attachment weakened by ocean acidification. Nature Climate Change 3: 587-590.

301. O'Donnell MJ, Hammond LM, Hofmann GE (2009) Predicted impact of ocean acidification on a marine invertebrate: elevated CO_2 alters response to thermal stress in sea urchin larvae. Marine Biology 156: 439-446.
302. O'Donnell MJ, Todgham AE, Sewell MA, Hammond LM, Ruggiero K, et al. (2010) Ocean acidification alters skeletogenesis and gene expression in larval sea urchins. Marine Ecology Progress Series 398: 157-171.
303. Olischlaeger M, Bartsch I, Gutow L, Wiencke C (2012) Effects of ocean acidification on different life-cycle stages of the kelp *Laminaria hyperborea* (Phaeophyceae). Botanica marina 55: 511-525.
304. Olischlaeger M, Bartsch I, Gutow L, Wiencke C (2013) Effects of ocean acidification on growth and physiology of *Ulva lactuca* (Chlorophyta) in a rockpool-scenario. Phycological Research 61: 180-190.
305. Olischlaeger M, Wiencke C (2013) Ocean acidification alleviates low-temperature effects on growth and photosynthesis of the red alga *Neosiphonia harveyi* (Rhodophyta). Journal of Experimental Biology 64: 5587-5597.
306. Padilla-Gamiño JL, Kelly MW, Evans TG, Hofmann GE (2013) Temperature and CO_2 additively regulate physiology, morphology and genomic responses of larval sea urchins, *Strongylocentrotus purpuratus*. Proceedings of the Royal Society B: Biological Sciences 280.
307. Paganini AW, Miller NA, Stillman JH (2014) Temperature and acidification variability reduce physiological performance in the intertidal zone porcelain crab *Petrolisthes cinctipes*. Journal of Experimental Biology 217: 3974-3980.
308. Pajusalu L, Martin G, Põllumäe A, Paalme T (2013) Results of laboratory and field experiments of the direct effect of increasing CO_2 on net primary production of macroalgal species in brackish-water ecosystems. Proceedings of the Estonian Academy of Science 62: 148-154.
309. Palacios SL, Zimmerman RC (2007) Response of eelgrass *Zostera marina* to CO_2 enrichment: possible impacts of climate change and potential for remediation of coastal habitat. Marine Ecology Progress Series 344: 1-13.
310. Pansch C, Nasrolahi A, Appelhans YS, Wahl M (2012) Impacts of ocean warming and acidification on the larval development of the barnacle *Amphibalanus improvisus*. Journal

of Experimental Marine Biology and Ecology 420-421: 48-55.
311. Pansch C, Nasrolahi A, Appelhans YS, Wahl M (2013) Tolerance of juvenile barnacles (*Amphibalanus improvisus*) to warming and elevated pCO(2). Marine Biology 160: 2023-2035.
312. Pansch C, Schaub I, Havenhand J, Wahl M (2014) Habitat traits and food availability determine the response of marine invertebrates to ocean acidification. Global Change Biology 20: 765-777.
313. Pansch C, Schlegel P, Havenhand J (2013) Larval development of the barnacle *Amphibalanus improvisus* responds variably but robustly to near-future ocean acidification. ICES Journal of Marine Science 70: 805-811.
314. Parker L, Pauline M R, Wayne A OC (2009) The effect of ocean acidification and temperature on the fertilization and embryonic development of the Sydney rock oyster *Saccostrea glomerata* (Gould 1850). Global Change Biology 15: 2123-2136.
315. Parker L, Ross P, O'Connor W (2010) Comparing the effect of elevated CO_2 and temperature on the fertilization and early development of two species of oysters. Marine Biology 157: 2435-2452.
316. Parker LM, Ross PM, O'Connor WA (2011) Populations of the Sydney rock oyster, *Saccostrea glomerata*, vary in response to ocean acidification. Marine Biology 158: 689-697.
317. Parker LM, Ross PM, O'Connor WA, Borysko L, Raftos DA, et al. (2012) Adult exposure influences offspring response to ocean acidification in oysters. Global Change Biology 18: 82-92.
318. Partridge V, Welch K, Aasen S, Dutch M (2005) Temporal monitoring of Puget Sound sediments: Results of the Puget Sound Ambient Monitoring Program, 1989-2000. Olympia, WA: WA Department of Ecology. 267 p.
319. Pascal P-Y, Fleeger JW, Galvez F, Carman KR (2010) The toxicological interaction between ocean acidity and metals in coastal meiobenthic copepods. Marine Pollution Bulletin 60: 2201-2208.
320. Pedrotti ML, Fiorini S, Kerros M-E, Middelburg JJ, Gattuso J-P (2012) Variable production of transparent exopolymeric particles by haploid and diploid life stages of coccolithophores grown under different CO_2 concentrations. Journal of Plankton

Research 34: 388-398.
321. Pespeni MH, Sanford E, Gaylord B, Hill TM, Hosfelt JD, et al. (2013) Evolutionary change during experimental ocean acidification. Proceedings of the National Academy of Sciences 110: 6937-6942.
322. Pettit LR, Hart MB, Medina-Sanchez AN, Smart CW, Rodolfo-Metalpa R, et al. (2013) Benthic foraminifera show some resilience to ocean acidification in the northern Gulf of California, Mexico. Marine Pollution Bulletin 73: 452-462.
323. Pimentel M, Pegado M, Repolho T, Rosa R (2014) Impact of ocean acidification in the metabolism and swimming behavior of the dolphinfish (*Coryphaena hippurus*) early larvae. Marine Biology 161: 725-729.
324. Pistevos JCA, Calosi P, Widdicombe S, Bishop JDD (2011) Will variation among genetic individuals influence species responses to global climate change? Oikos 120: 675-689.
325. Place SP, Smith BW (2012) Effects of seawater acidification on cell cycle control mechanisms in *Strongylocentrotus purpuratus* embryos. PLoS ONE 7: e34068.
326. Porzio L, Buia MC, Hall-Spencer JM (2011) Effects of ocean acidification on macroalgal communities. Journal of Experimental Marine Biology and Ecology 400: 278-287.
327. Porzio L, Garrard SL, Buia MC (2013) The effect of ocean acidification on early algal colonization stages at natural CO_2 vents. Marine Biology: 1-13.
328. Race Rocks (2011) http://www.racerocks.com
329. Raddatz J, Rueggeberg A, Floegel S, Hathorne EC, Liebetrau V, et al. (2014) The influence of seawater pH on U/Ca ratios in the scleractinian cold-water coral *Lophelia pertusa*. Biogeosciences 11: 1863-1871.
330. Ragazzola F, Foster LC, Form A, Anderson PSL, Hansteen TH, et al. (2012) Ocean acidification weakens the structural integrity of coralline algae. Global Change Biology 18: 2804-2812.
331. Range P, Chícharo MA, Ben-Hamadou R, Piló D, Matias D, et al. (2011) Calcification, growth and mortality of juvenile clams *Ruditapes decussatus* under increased pCO_2 and reduced pH: variable responses to ocean acidification at local scales? Journal of Experimental Marine Biology and Ecology 396: 177-184.
332. Range P, Piló D, Ben-Hamadou R, Chícharo MA, Matias D, et al. (2012) Seawater acidification by CO_2 in a coastal lagoon environment: Effects on life history traits of

juvenile mussels *Mytilus galloprovincialis*. Journal of Experimental Marine Biology and Ecology 424-425: 89-98.

333. Reuter KE, Lotterhos KE, Crim RN, Thompson CA, Harley CDG (2011) Elevated pCO_2 increases sperm limitation and risk of polyspermy in the red sea urchin *Strongylocentrotus franciscanus*. Global Change Biology 17: 163-171.

334. Richier S, Fiorini S, Kerros M-E, von Dassow P, Gattuso J-P (2011) Response of the calcifying coccolithophore *Emiliania huxleyi* to low pH/high *p*CO_2: from physiology to molecular level. Marine Biology 158: 551-560.

335. Ries J, Cohen A, McCorkle D (2010) A nonlinear calcification response to CO_2-induced ocean acidification by the coral *Oculina arbuscula*. Coral Reefs 29: 661-674.

336. Ries JB, Cohen AL, McCorkle DC (2009) Marine calcifiers exhibit mixed responses to CO_2-induced ocean acidification. Geology 37: 1131-1134.

337. Roberts C (2013) Phototaxis of Dungeness crab zoea in high CO_2 seawater: implications for coastal ecosystems in an acidified ocean The Evergreen State College. 64 p.

338. Roberts DA, Birchenough SNR, Lewis C, Sanders MB, Bolam T, et al. (2013) Ocean acidification increases the toxicity of contaminated sediments. Global Change Biology 19: 340-351.

339. Robertson AI (1979) The relationship between annual production: biomass ratios and lifespans for marine macrobenthos. Oecologia 38: 193-202.

340. Rodolfo-Metalpa R, Houlbreque F, Tambutte E, Boisson F, Baggini C, et al. (2011) Coral and mollusc resistance to ocean acidification adversely affected by warming. Nature Climate Change 1: 308-312.

341. Rodolfo-Metalpa R, Martin S, Ferrier-Pages C, Gattuso JP (2010) Response of the temperate coral *Cladocora caespitosa* to mid- and long-term exposure to pCO_2 and temperature levels projected for the year 2100 AD. Biogeosciences 7: 289-300.

342. Rokitta SD, John U, Rost B (2012) Ocean acidification affects redox-balance and ion-homeostasis in the life-cycle stages of *Emiliania huxleyi*. PLoS ONE 7: e52212.

343. Rokitta SD, Rost B (2012) Effects of CO_2 and their modulation by light in the life-cycle stages of the coccolithophore *Emiliania huxleyi* Limnology and Oceanography 57: 607-618.

344. Roleda MY, Morris JN, McGraw CM, Hurd CL (2012) Ocean acidification and seaweed

reproduction: increased CO_2 ameliorates the negative effect of lowered pH on meiospore germination in the giant kelp *Macrocystis pyrifera* (Laminariales, Phaeophyceae). Global Change Biology 18: 854-864.
345. Rosa R, Baptista M, Lopes VM, Pegado MR, Paula JR, et al. (2014) Early-life exposure to climate change impairs tropical shark survival. Proceedings of the Royal Society B-Biological Sciences 281: 7.
346. Rosa R, Seibel BA (2008) Synergistic effects of climate-related variables suggest future physiological impairment in a top oceanic predator. Proceedings of the National Academy of Sciences 105: 20776-20780.
347. Rossoll D, Bermúdez R, Hauss H, Schulz KG, Riebesell U, et al. (2012) Ocean acidification-induced food quality deterioration constrains trophic transfer. PLoS ONE 7: e34737.
348. Rubio-Portillo E, Vázquez-Luis M, Izquierdo Muñoz A, Ramos Esplá AA (2014) Distribution patterns of alien coral *Oculina patagonica* De Angelis D'Ossat, 1908 in western Mediterranean Sea. Journal of Sea Research 85: 372-378.
349. Saba GK, Schofield O, Torres JJ, Ombres EH, Steinberg DK (2012) Increased feeding and nutrient excretion of adult Antarctic krill, *Euphausia superba*, exposed to enhanced carbon dioxide (CO_2). PLoS ONE 7: e52224.
350. Sanford E, Gaylord B, Hettinger A, Lenz EA, Meyer K, et al. (2014) Ocean acidification increases the vulnerability of native oysters to predation by invasive snails. Proceedings of the Royal Society B-Biological Sciences 281.
351. Scagel RF, Gabrielson PW, Garbary DJ, Golden L, Hawkes MW, et al. (1989) A synopsis of the benthic marine algae of British Columbia, Southeast Alaska, Washington and Oregon. Vancouver, Canada: University of British Columbia. 532 p.
352. Schade FM, Clemmesen C, Wegner KM (2014) Within- and transgenerational effects of ocean acidification on life history of marine three-spined stickleback (*Gasterosteus aculeatus*). Marine Biology 161: 1667-1676.
353. Schalkhausser B, Bock C, Stemmer K, Brey T, Pörtner H-O, et al. (2012) Impact of ocean acidification on escape performance of the king scallop, *Pecten maximus*, from Norway. Marine Biology: 1-12.
354. Schaum E, Rost B, Millar AJ, Collins S (2013) Variation in plastic responses of a globally

distributed picoplankton species to ocean acidification. Nature Climate Change 3: 298-302.

355. Schlegel P, Havenhand JN, Gillings MR, Williamson JE (2012) Individual variability in reproductive success determines winners and losers under ocean acidification: a case study with sea urchins. PLoS ONE 7: e53118.

356. Schram JB, McClintock JB, Angus RA, Lawrence JM (2011) Regenerative capacity and biochemical composition of the sea star *Luidia clathrata* (Say) (Echinodermata: Asteroidea) under conditions of near-future ocean acidification. Journal of Experimental Marine Biology and Ecology 407: 266-274.

357. Seibel BA, Maas AE, Dierssen HM (2012) Energetic plasticity underlies a variable response to ocean acidification in the pteropod, *Limacina helicina antarctica*. PLoS ONE 7: e30464.

358. Sett S, Bach LT, Schulz KG, Koch-Klavsen S, Lebrato M, et al. (2014) Temperature modulates coccolithophorid sensitivity of growth, photosynthesis and calcification to increasing seawater pCO_2. PLoS ONE 9: e88308.

359. Shellenberger JS, Ross JRP (1998) Antibacterial activity of two species of bryozoans from northern Puget Sound. Northwest Science 72: 23-33.

360. Shi D, Kranz SA, Kim J-M, Morel FMM (2012) Ocean acidification slows nitrogen fixation and growth in the dominant diazotroph *Trichodesmium* under low-iron conditions. Proceedings of the National Academy of Sciences 109: E3094–E3100.

361. Shi D, Xu Y, Morel FMM (2009) Effects of the pH/pCO_2 control method on medium chemistry and phytoplankton growth. Biogeosciences 6: 1199-1207.

362. Shin HH, Jung SW, Jang M-C, Kim Y-O (2013) Effect of pH on the morphology and viability of *Scrippsiella trochoidea* cysts in the hypoxic zone of a eutrophied area. Harmful Algae 28: 37-45.

363. Skidmore D, Chew KK (1985) Mussel aquaculture in Puget Sound. Seattle, WA: Washington Sea Grant. 57 p.

364. Small D, Calosi P, White D, Spicer JI, Widdicombe S (2010) Impact of medium-term exposure to CO_2 enriched seawater on the physiological functions of the velvet swimming crab *Necora puber*. Aquatic Biology 10: 11-21.

365. Smith GF (1976) A quantitative sampling program of benthic communities in nearshore

subtidal areas within the Rosario Strait region of northern Puget Sound. Bellingham: Western Washington University, Huxley College.

366. Speck C (2007) Shellfish surveys of Puget Sound beaches. WA Department of Fish and Wildlife.

367. Spicer JI, Raffo A, Widdicombe S (2007) Influence of CO_2-related seawater acidification on extracellular acid-base balance in the velvet swimming crab *Necora puber*. Marine Biology 151: 1117-1125.

368. Spicer JI, Widdicombe S (2012) Acute extracellular acid–base disturbance in the burrowing sea urchin *Brissopsis lyrifera* during exposure to a simulated CO_2 release. Science of The Total Environment 427–428: 203-207.

369. Spicer JI, Widdicombe S, Needham HR, Berge JA (2011) Impact of CO_2-acidified seawater on the extracellular acid–base balance of the northern sea urchin *Strongylocentrotus dröebachiensis*. Journal of Experimental Marine Biology and Ecology 407: 19-25.

370. Spielmeyer A, Pohnert G (2012) Influence of temperature and elevated carbon dioxide on the production of dimethylsulfoniopropionate and glycine betaine by marine phytoplankton. Marine Environmental Research 73: 62-69.

371. Stumpp M, Dupont S, Thorndyke MC, Melzner F (2011) CO_2 induced seawater acidification impacts sea urchin larval development II: gene expression patterns in pluteus larvae. Comparative Biochemistry and Physiology Part A: Molecular & Integrative Physiology 160: 320-330.

372. Stumpp M, Hu M, Casties I, Saborowski R, Bleich M, et al. (2013) Digestion in sea urchin larvae impaired under ocean acidification. Nature Climate Change 3: 1044-1049.

373. Stumpp M, Hu MY, Melzner F, Gutowska MA, Dorey N, et al. (2012) Acidified seawater impacts sea urchin larvae pH regulatory systems relevant for calcification. Proceedings of the National Academy of Sciences 109: 18192-18197.

374. Stumpp M, Wren J, Melzner F, Thorndyke MC, Dupont ST (2011) CO_2 induced seawater acidification impacts sea urchin larval development I: elevated metabolic rates decrease scope for growth and induce developmental delay. Comparative Biochemistry and Physiology Part A: Molecular & Integrative Physiology 160: 331-340.

375. Suggett DJ, Hall-Spencer JM, Rodolfo-Metalpa R, Boatman TG, Payton R, et al. (2012) Sea anemones may thrive in a high CO_2 world. Global Change Biology 18: 3015-3025.

376. Sugie K, Yoshimura T (2013) Effects of pCO_2 and iron on the elemental composition and cell geometry of the marine diatom *Pseudo-nitzschia pseudodelicatissima* (Bacillariophyceae). Journal of Phycology 49: 475-488.

377. Sunday JM, Crim RN, Harley CDG, Hart MW (2012) Quantifying rates of evolutionary adaptation in response to ocean acidification. PLoS ONE 6: e22881.

378. Sundin J, Rosenqvist G, Berglund A (2013) Altered oceanic pH impairs mating propensity in a pipefish. Ethology 119: 86-93.

379. Swanson AK, Fox CH (2007) Altered kelp (Laminariales) phlorotannins and growth under elevated carbon dioxide and ultraviolet-B treatments can influence associated intertidal food webs. Global Change Biology 13: 1696-1709.

380. Tait LW (2014) Impacts of natural and manipulated variations in temperature, pH and light on photosynthetic parameters of coralline-kelp assemblages. Journal of Experimental Marine Biology and Ecology 454: 1-8.

381. Talmage SC, Gobler CJ (2010) Effects of past, present, and future ocean carbon dioxide concentrations on the growth and survival of larval shellfish. Proceedings of the National Academy of Sciences 107: 17246-17251.

382. Talmage SC, Gobler CJ (2012) Effects of CO_2 and the harmful alga *Aureococcus anophagefferens* on growth and survival of oyster and scallop larvae. Marine Ecology Progress Series 464: 121-134.

383. Talmage SC, Gobler CJ (2012) Effects of elevated temperature and carbon dioxide on the growth and survival of larvae and juveniles of three species of Northwest Atlantic bivalves. PLoS ONE 6: e26941.

384. Talmage SC, Golber CJ (2009) The effects of elevated carbon dioxide concentrations on the metamorphosis, size, and survival of larval hard clams (*Mercenaria mercenaria*), bay scallops (*Argopecten irradians*), and Eastern oysters (*Crassostrea virginica*). Limnology and Oceanography 54: 2072-2080.

385. Tatters AO, Flewelling LJ, Fu F, Granholm AA, Hutchins DA (2013) High CO_2 promotes the production of paralytic shellfish poisoning toxins by *Alexandrium catenella* from Southern California waters. Harmful Algae 30: 37-43.

386. Tatters AO, Fu F-X, Hutchins DA (2012) High CO_2 and silicate limitation synergistically increase the toxicity of *Pseudo-nitzschia fraudulenta*. PLoS ONE 7: e32116.

387. Tatters AO, Schnetzer A, Fu F, Lie AYA, Caron DA, et al. (2013) Short- versus long-term responses to changing CO_2 in a coastal dinoflagellate bloom: implications for interspecific competitive interactions and community structure. Evolution 67: 1879-1891.
388. Taylor JR, Lovera C, Whaling PJ, Buck KR, Pane EF, et al. (2014) Physiological effects of environmental acidification in the deep-sea urchin *Strongylocentrotus fragilis*. Biogeosciences 11: 1413-1423.
389. Thiyagarajan V, Ko GWK (2012) Larval growth response of the Portuguese oyster (*Crassostrea angulata*) to multiple climate change stressors. Aquaculture 370-371: 90-95.
390. Thom RM (1996) CO_2-enrichment effects on eelgrass (*Zostera marina* L) and bull kelp (*Nereocystis leutkeana* (Mert.) P. & R.). Water, Air, and Soil Pollution 88: 383-391.
391. Thomsen J, Casties I, Pansch C, Körtzinger A, Melzner F (2013) Food availability outweighs ocean acidification effects in juvenile *Mytilus edulis*: laboratory and field experiments. Global Change Biology 19: 1017-1027.
392. Thomsen J, Gutowska MA, Saphörster J, Heinemann A, Trübenbach K, et al. (2010) Calcifying invertebrates succeed in a naturally CO_2-rich coastal habitat but are threatened by high levels of future acidification Biogeosciences 7: 3879-3891.
393. Thomsen J, Melzner F (2010) Moderate seawater acidification does not elicit long-term metabolic depression in the blue mussel *Mytilus edulis*. Marine Biology 157: 2667-2676.
394. Thorpe JP, Ryland JS, Beardmore JA (1978) Genetic variation and biochemical systematics in the marine bryozoan *Alcyonidium mytili*. Marine Biology 49: 343-350.
395. Timmins-Schiffman E, O'Donnell MJ, Friedman CS, Roberts SB (2013) Elevated pCO_2 causes developmental delay in early larval Pacific oysters, *Crassostrea gigas*. Marine Biology 160: 1973-1982.
396. Todgham AE, Hofmann GE (2009) Transcriptomic response of sea urchin larvae *Strongylocentrotus purpuratus* to CO_2-driven seawater acidification. Journal of Experimental Biology 212: 2579-2594.
397. Troedsson C, Bouquet J-M, Lobon CM, Novac A, Nejstgaard JC, et al. (2013) Effects of ocean acidification, temperature and nutrient regimes on the appendicularian *Oikopleura dioica*: a mesocosm study. Marine Biology 160: 2175-2187.
398. Tseng YC, Hu MY, Stumpp M, Lin LY, Melzner F, et al. (2013) CO_2-driven seawater

acidification differentially affects development and molecular plasticity along life history of fish (*Oryzias latipes*). Comparative Biochemistry and Physiology a-Molecular & Integrative Physiology 165: 119-130.

399. Walla Walla University (2011) http://www.wallawalla.edu/academics/departments/biology/rosario/inverts/
400. Van Colen C, Debusschere E, Braeckman U, Van Gansbeke D, Vincx M (2012) The early life history of the clam *Macoma balthica* in a high CO_2 world. PLoS ONE 7: e44655.
401. Van de Waal DB, John U, Ziveri P, Reichart G-J, Hoins M, et al. (2013) Ocean acidification reduces growth and calcification in a marine dinoflagellate. PLoS ONE 8: e65987.
402. Vargas CA, de la Hoz M, Aguilera V, Martín VS, Manríquez PH, et al. (2013) CO_2-driven ocean acidification reduces larval feeding efficiency and change food selectivity in the mollusk *Concholepas concholepas*. Journal of Plankton Research 35: 1059-1068.
403. Vehmaa A, Brutemark A, Engström-Öst J (2012) Maternal effects may act as an adaptation mechanism for copepods facing pH and temperature changes. PLoS ONE 7: e48538.
404. Vihtakari M, Hendriks IE, Holding J, Renaud PE, Duarte CM, et al. (2013) Effects of ocean acidification and warming on sperm activity and early life stages of the Mediterranean Mussel (*Mytilus galloprovincialis*). Water 5: 1890-1915.
405. Waldbusser GG, Bergschneider H, Green MA (2010) Size-dependent pH effect on calcification in post-larval hard clam *Mercenaria spp.* Marine Ecology Progress Series 417: 171-182.
406. Waldbusser GG, Voigt EP, Bergschneider H, Green MA, Newell RE (2011) Biocalcification in the eastern oyster (*Crassostrea virginica*) in relation to long-term trends in Chesapeake Bay pH. Estuaries and Coasts 34: 221-231.
407. Walther K, Anger K, Pörtner HO (2010) Effects of ocean acidification and warming on the larval development of the spider crab *Hyas araneus* from different latitudes (54º vs. 79º N). Marine Ecology Progress Series 417: 159-170.
408. Walther K, Sartoris FJ, Bock C, Pörtner HO (2009) Impact of anthropogenic ocean acidification on thermal tolerance of the spider crab *Hyas araneus*. Biogeosciences 6: 2207-2215.
409. Weitkamp LA (1994) Environmental monitoring of the Manchester Naval fuel pier replacement, Puget Sound, Washington. 92 p.

410. Welladsen H, Southgate PC, Heimann K (2010) The effects of exposure to near-future levels of ocean acidification on shell characteristics of *Pinctada fucata* (Bivalvia: Pteriidae). Molluscan Research 30: 125-130.
411. Weydmann A, SØreide JE, Kwasniewski S, Widdicombe S (2012) Influence of CO_2-induced acidification on the reproduction of a key Arctic copepod *Calanus glacialis*. Journal of Experimental Marine Biology and Ecology 428: 39-42.
412. White MM, McCorkle DC, Mullineaux LS, Cohen AL (2013) Early exposure of bay scallops (*Argopecten irradians*) to high CO_2 causes a decrease in larval shell growth. PLoS ONE 8: e61065.
413. Widdicombe S, Beesley A, Berge JA, Dashfield SL, McNeill CL, et al. (2013) Impact of elevated levels of CO_2 on animal mediated ecosystem function: the modification of sediment nutrient fluxes by burrowing urchins. Marine Pollution Bulletin 73: 416-427.
414. Widdicombe S, Needham HR (2007) Impact of CO_2 induced seawater acidification on the burrowing activity of *Nereis virens* (Sars 1835) and sediment nutrient flux. Marine Ecology Progress Series 341: 111-122.
415. Wolfe K, Dworjanyn SA, Byrne M (2013) Effects of ocean warming and acidification on survival, growth and skeletal development in the early benthic juvenile sea urchin (*Heliocidaris erythrogramma*). Global Change Biology 19: 2698-2707.
416. Wong KKW, Lane AC, Leung PTY, Thiyagarajan V (2011) Response of larval barnacle proteome to CO_2-driven seawater acidification. Comparative Biochemistry and Physiology Part D: Genomics and Proteomics 6: 310-321.
417. Wood HL, Spicer JI, Lowe DM, Widdicombe S (2010) Interaction of ocean acidification and temperature; the high cost of survival in the brittlestar *Ophiura ophiura*. Marine Biology 157: 2001-2013.
418. Wood HL, Spicer JI, Widdicombe S (2008) Ocean acidification may increase calcification rates, but at a cost. Proceedings of the Royal Society B 275: 1767-1773.
419. Wood HL, Widdicombe S, Spicer JI (2009) The influence of hypercapnia and the infaunal brittlestar *Amphiura filiformis* on sediment nutrient flux - will ocean acidification affect nutrient exchange? Biogeosciences 6: 2015-2024.
420. Wright JM, Parker LM, O'Connor WA, Williams M, Kube P, et al. (2014) Populations of Pacific oysters *Crassostrea gigas* respond variably to elevated CO_2 and predation by

Morula marginalba. Biological Bulletin 226: 269-281.

421. WSU Beach Watchers (2007) EZ-ID guides. Washington State University.

422. Wu X, Gao G, Giordano M, Gao K (2012) Growth and photosynthesis of a diatom grown under elevated CO_2 in the presence of solar UV radiation. Fundamental and Applied Limnology 180: 279-290.

423. Wu Y, Gao K, Riebesell U (2010) CO_2-induced seawater acidification affects physiological performance of the marine diatom *Phaeodactylum tricornutum*. Biogeosciences 7: 2915-2923.

424. Yang GY, Gao KS (2012) Physiological responses of the marine diatom *Thalassiosira pseudonana* to increased pCO_2 and seawater acidity Marine Environmental Research 79: 142-151.

425. Yu PC, Matson PG, Martz TR, Hofmann GE (2012) The ocean acidification seascape and its relationship to the performance of calcifying marine invertebrates: Laboratory experiments on the development of urchin larvae framed by environmentally-relevant pCO_2/pH. Journal of Experimental Marine Biology and Ecology 400: 288-295.

426. Zhang D, Li S, Wang G, Guo D (2012) Impacts of CO_2-driven seawater acidification on survival, egg production rate and hatching success of four marine copepods. Acta Oceanologica Sinica 30: 86-94.

427. Zhang M, Fang J, Zhang J, Li B, Ren S, et al. (2011) Effect of marine acidification on calcification and respiration of *Chlamys farreri*. Journal of Shellfish Research 30: 267-271.

428. Zippay ML, Hofmann GE (2010) Effect of pH on gene expression and thermal tolerance of early life history stages of red abalone (*Haliotis rufescens*). Journal of Shellfish Research 29: 429-439.
