# Supplementary figures and images for: Estimates of the Direct Effect of Seawater pH on the Survival Rate of Species Groups in the California Current Ecosystem
Source: PLoS One. 2016 Aug 11;11(8):e0160669. doi: 10.1371/journal.pone.0160669 (PMC4981315; doi:10.1371/journal.pone.0160669)

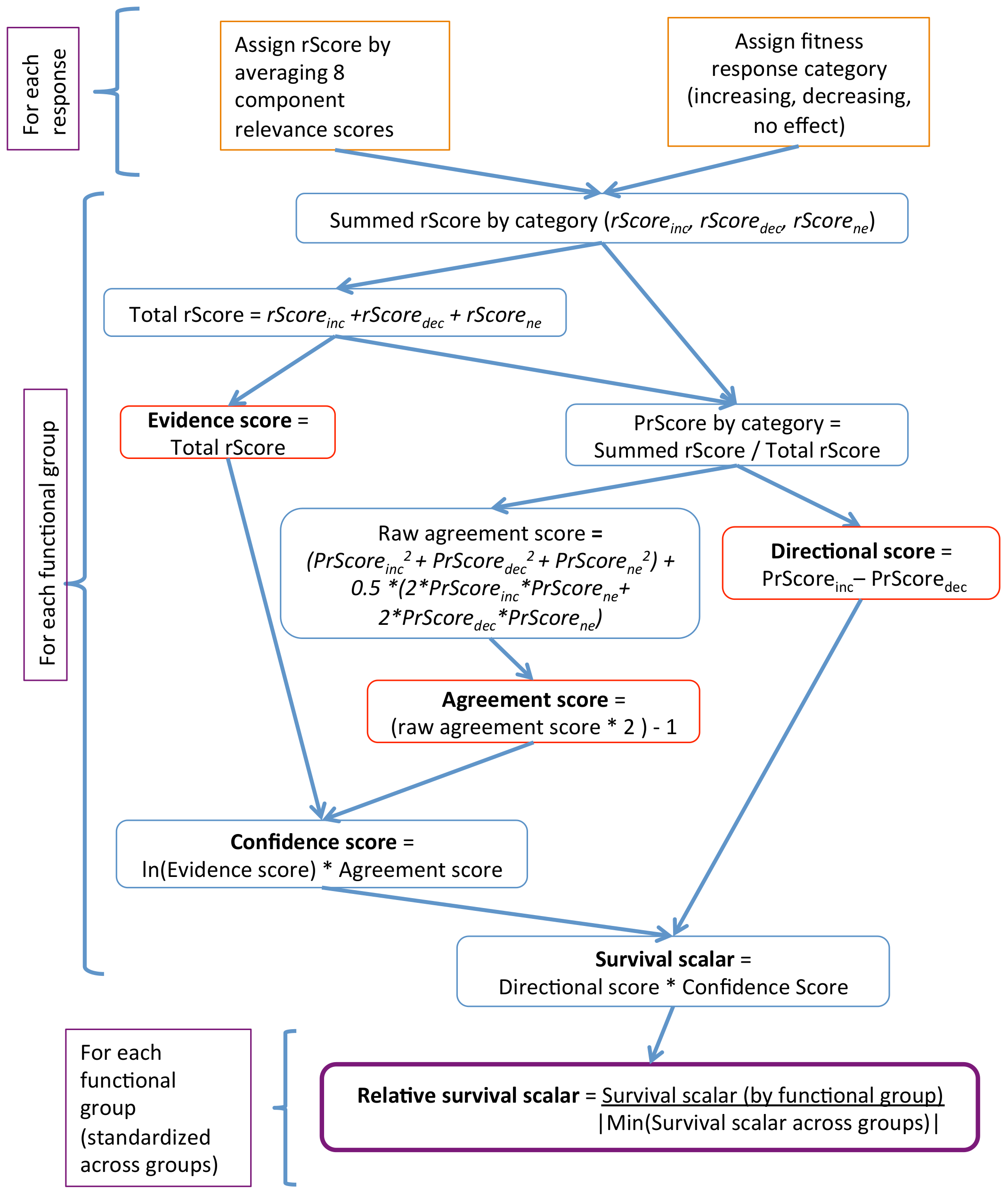

Supplement: S1 Fig — (TIF) [file pone.0160669.s003.tif]

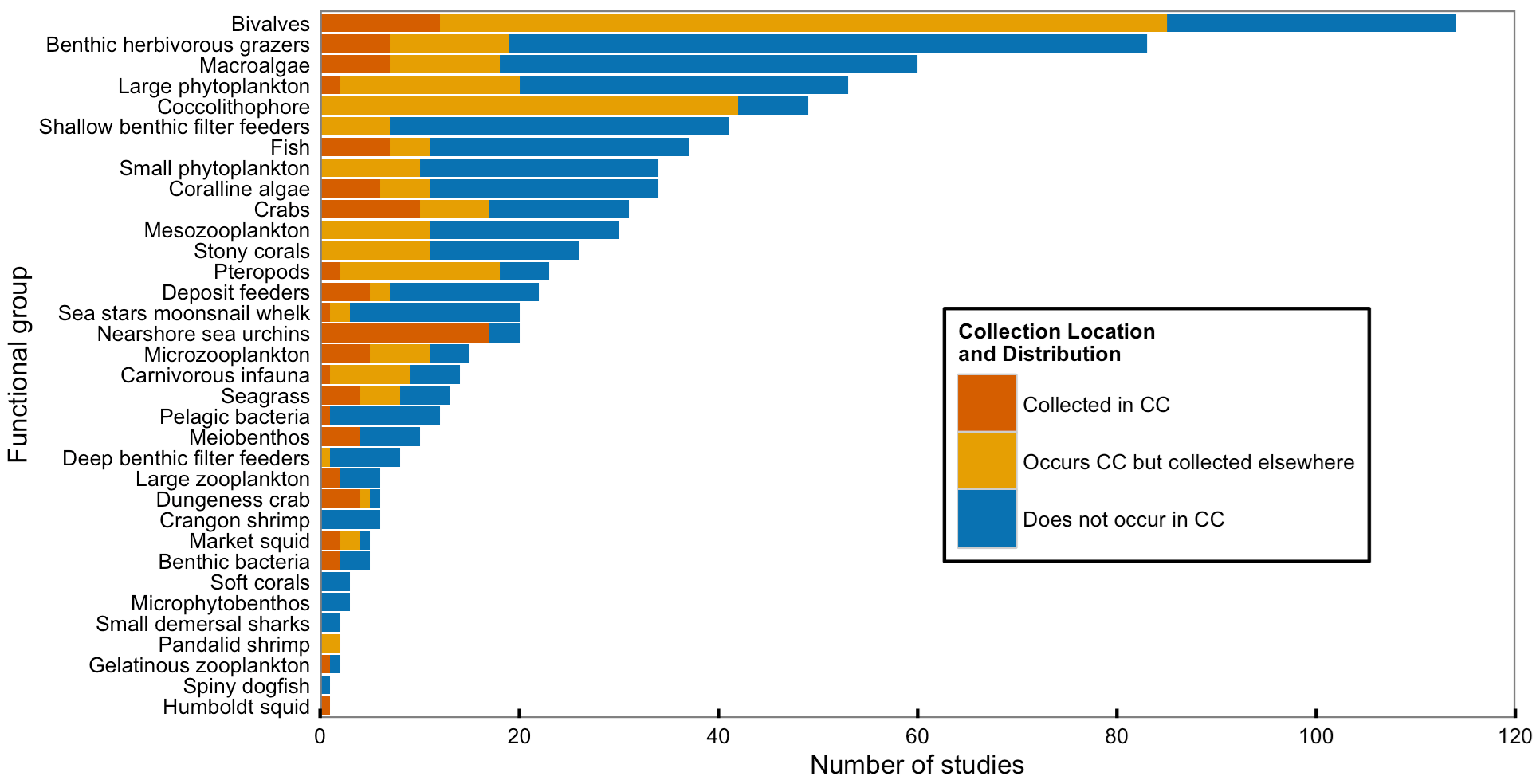

Supplement: S2 Fig — Number of studies for each functional group with information on whether the species studied is distributed in the California Current (CC) and study subjects are collected from the California Current. (TIF) [file pone.0160669.s004.tif]

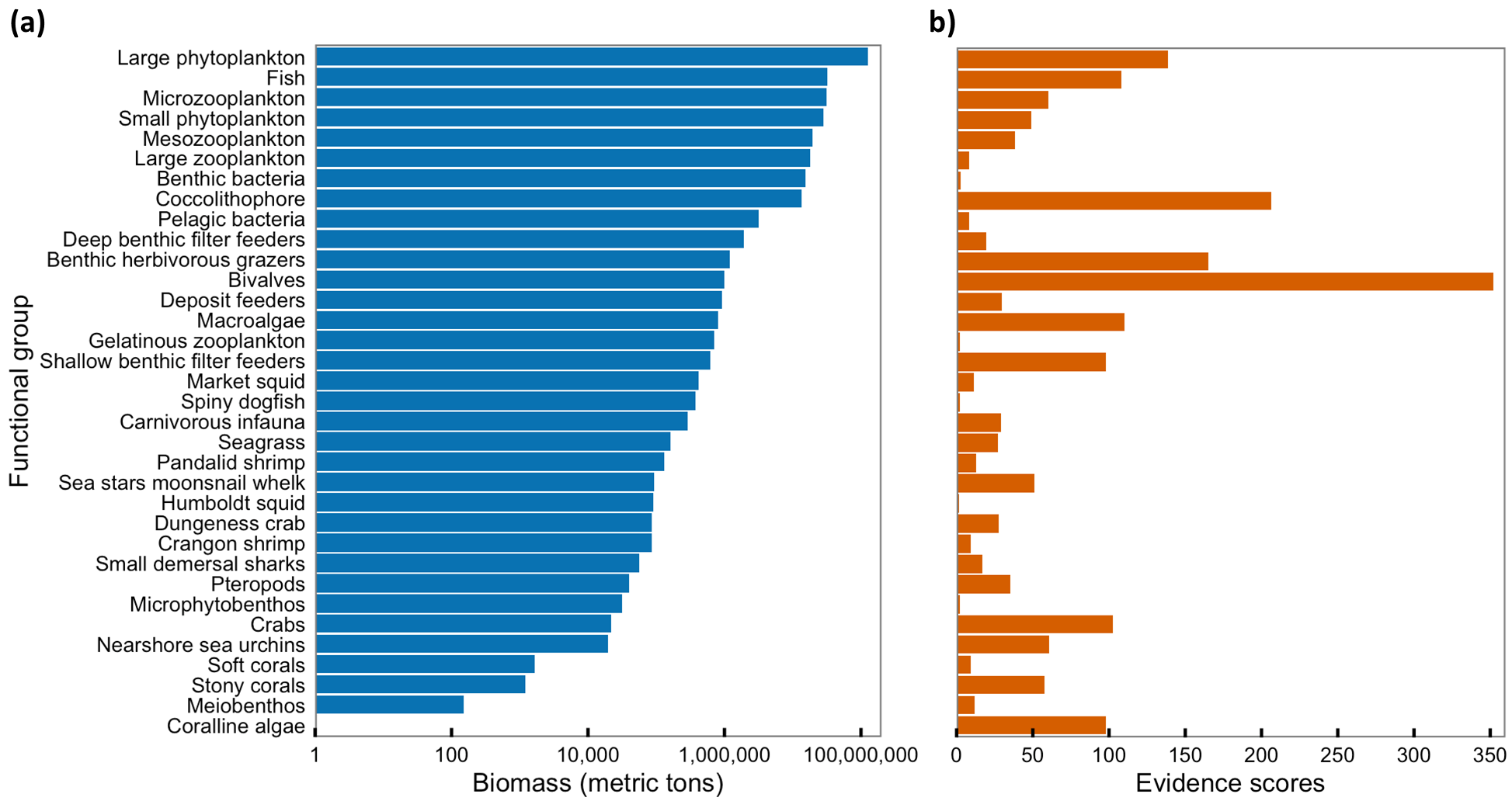

Supplement: S4 Fig — The (a) biomass and (b) evidence score for each functional group in the California Current ecosystem model. Biomass is on a log10 scale. (TIF) [file pone.0160669.s006.tif]
