# Supplementary material for: Estimates of the Direct Effect of Seawater pH on the Survival Rate of Species Groups in the California Current Ecosystem
Source: PLoS One. 2016 Aug 11;11(8):e0160669. doi: 10.1371/journal.pone.0160669 (PMC4981315; doi:10.1371/journal.pone.0160669)
Supplement: S3 Fig — pH survival sensitivity curves for all functional groups in the California Current ecosystem model and some functional groups not in the model (coralline algae, fish) for which published literature exists on species response to carbonate chemistry conditions. (PDF) [file pone.0160669.s005.pdf]

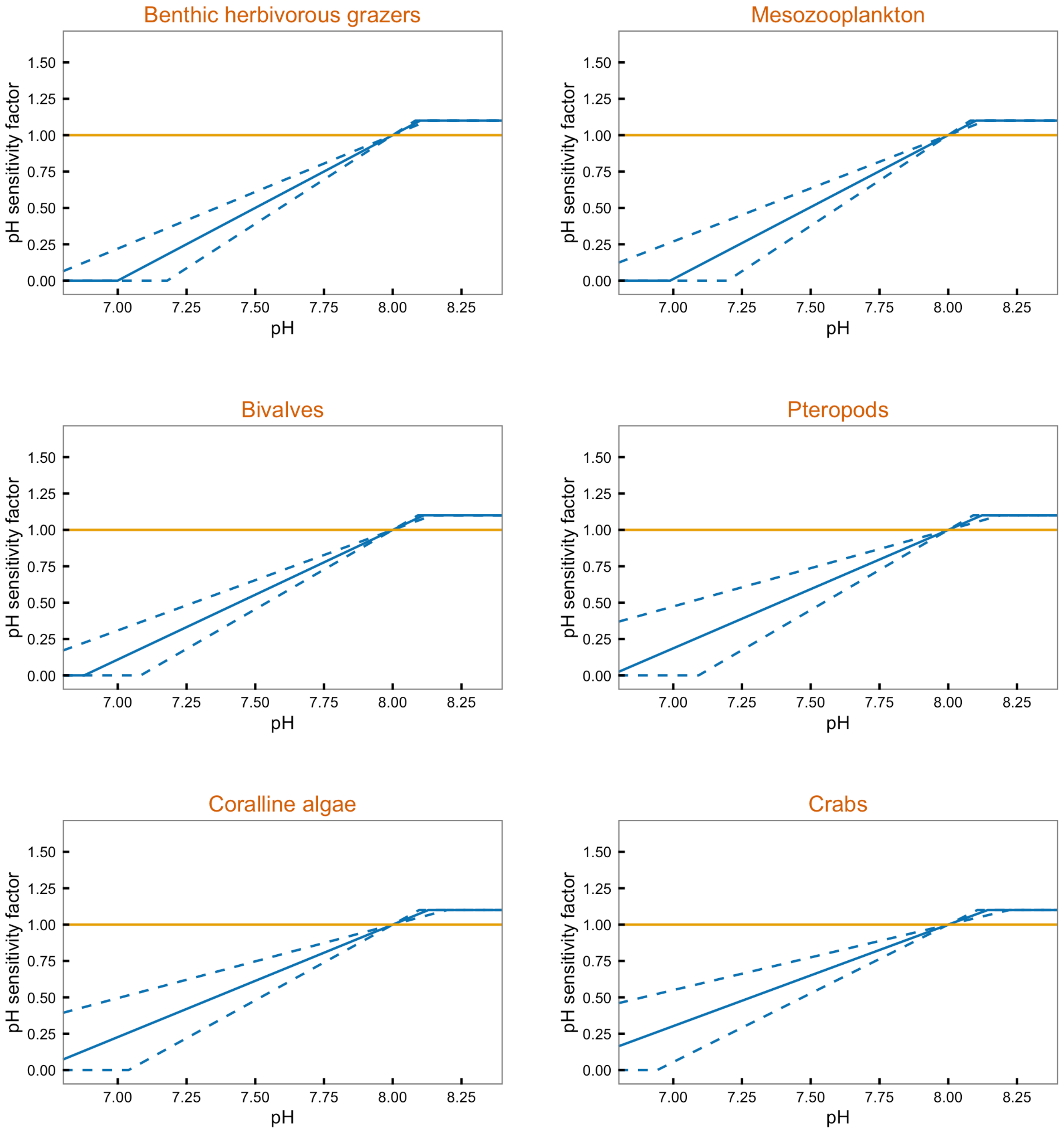
Benthic herbivorous grazers
Mesozooplankton
Bivalves
Pteropods
Coralline algae
Crabs
pH sensitivity factor
pH
0.00
0.25
0.50
0.75
1.00
1.25
1.50
7.00
7.25
7.50
7.75
8.00
8.25

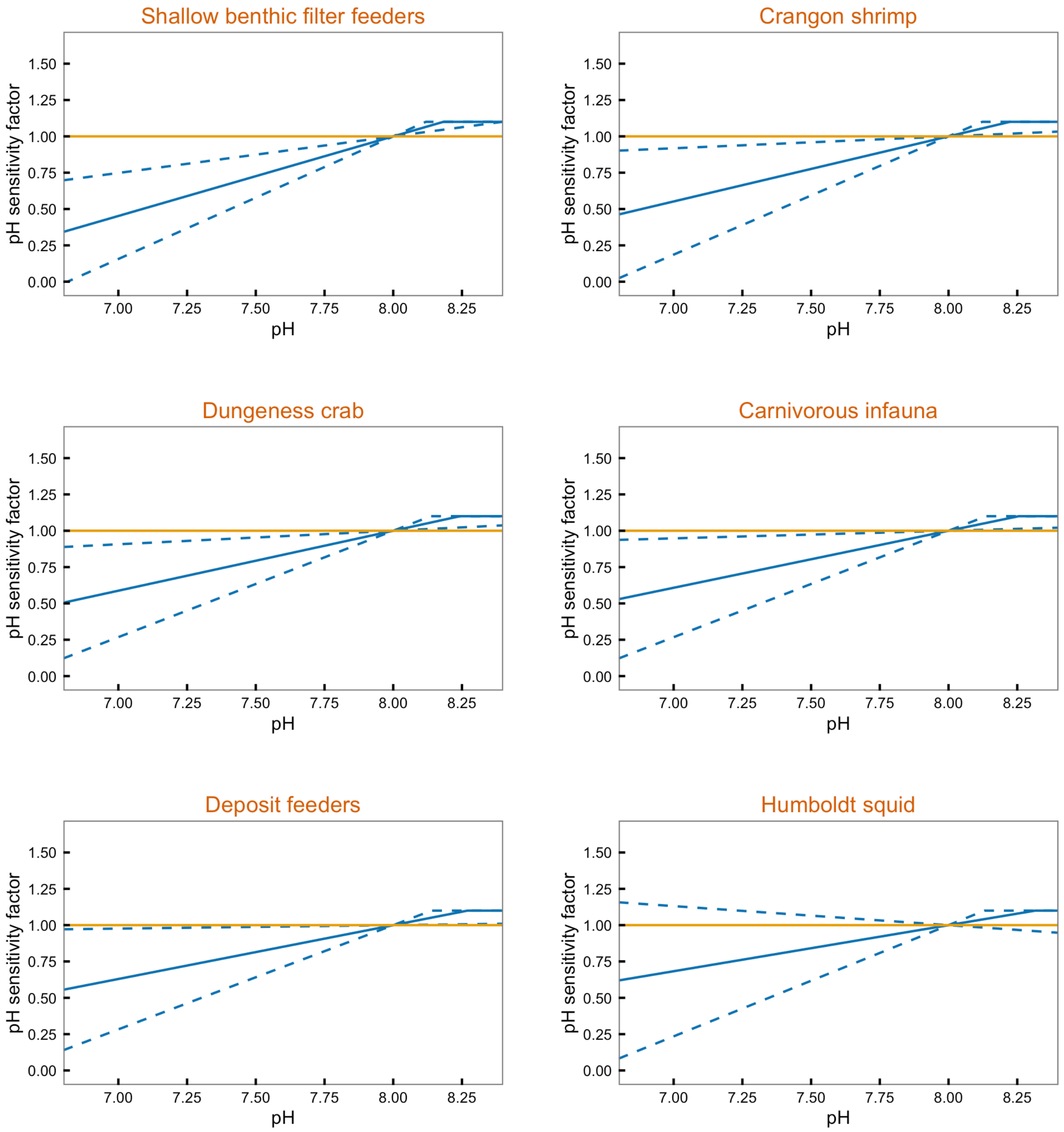
Shallow benthic filter feeders
Crangon shrimp
Dungeness crab
Carnivorous infauna
Deposit feeders
Humboldt squid
pH sensitivity factor
pH
0.00
0.25
0.50
0.75
1.00
1.25
1.50
7.00
7.25
7.50
7.75
8.00
8.25

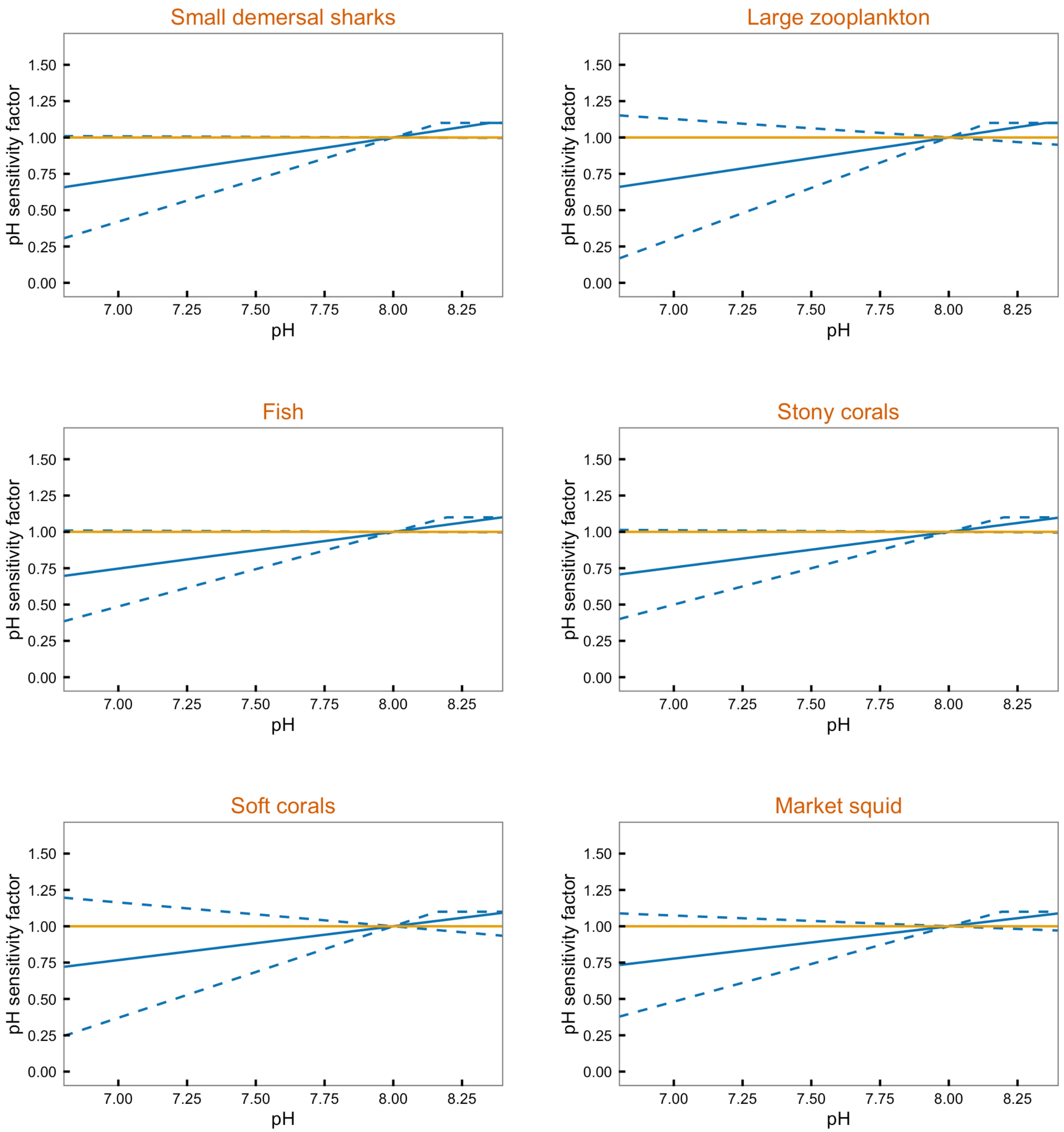
Small demersal sharks
Large zooplankton
Fish
Stony corals
Soft corals
Market squid
pH sensitivity factor
pH
0.00
0.25
0.50
0.75
1.00
1.25
1.50
7.00
7.25
7.50
7.75
8.00
8.25

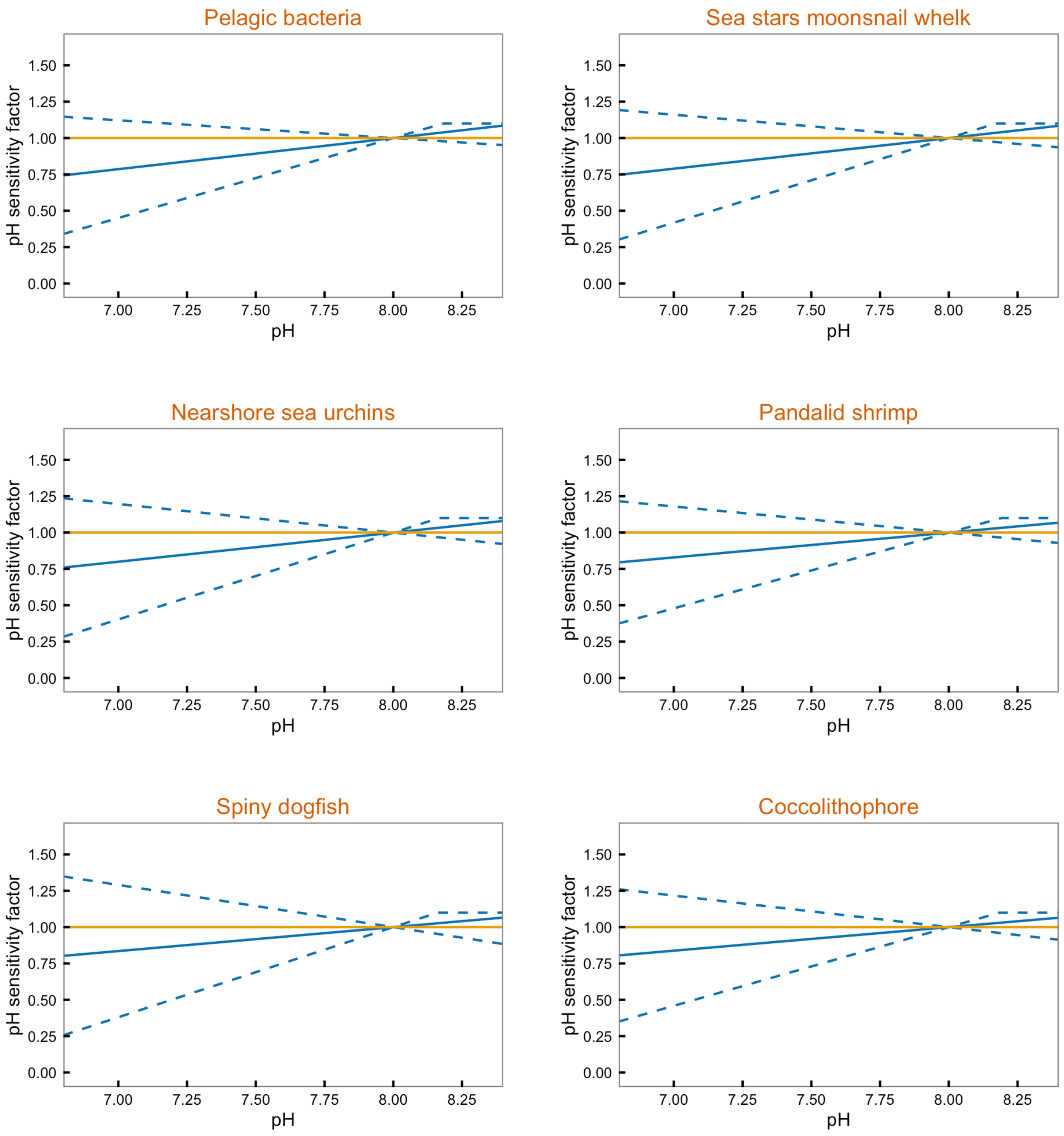
Pelagic bacteria
Sea stars moonsnail whelk
Nearshore sea urchins
Pandalid shrimp
Spiny dogfish
Coccolithophore
pH sensitivity factor
pH
0.00
0.25
0.50
0.75
1.00
1.25
1.50
7.00
7.25
7.50
7.75
8.00
8.25

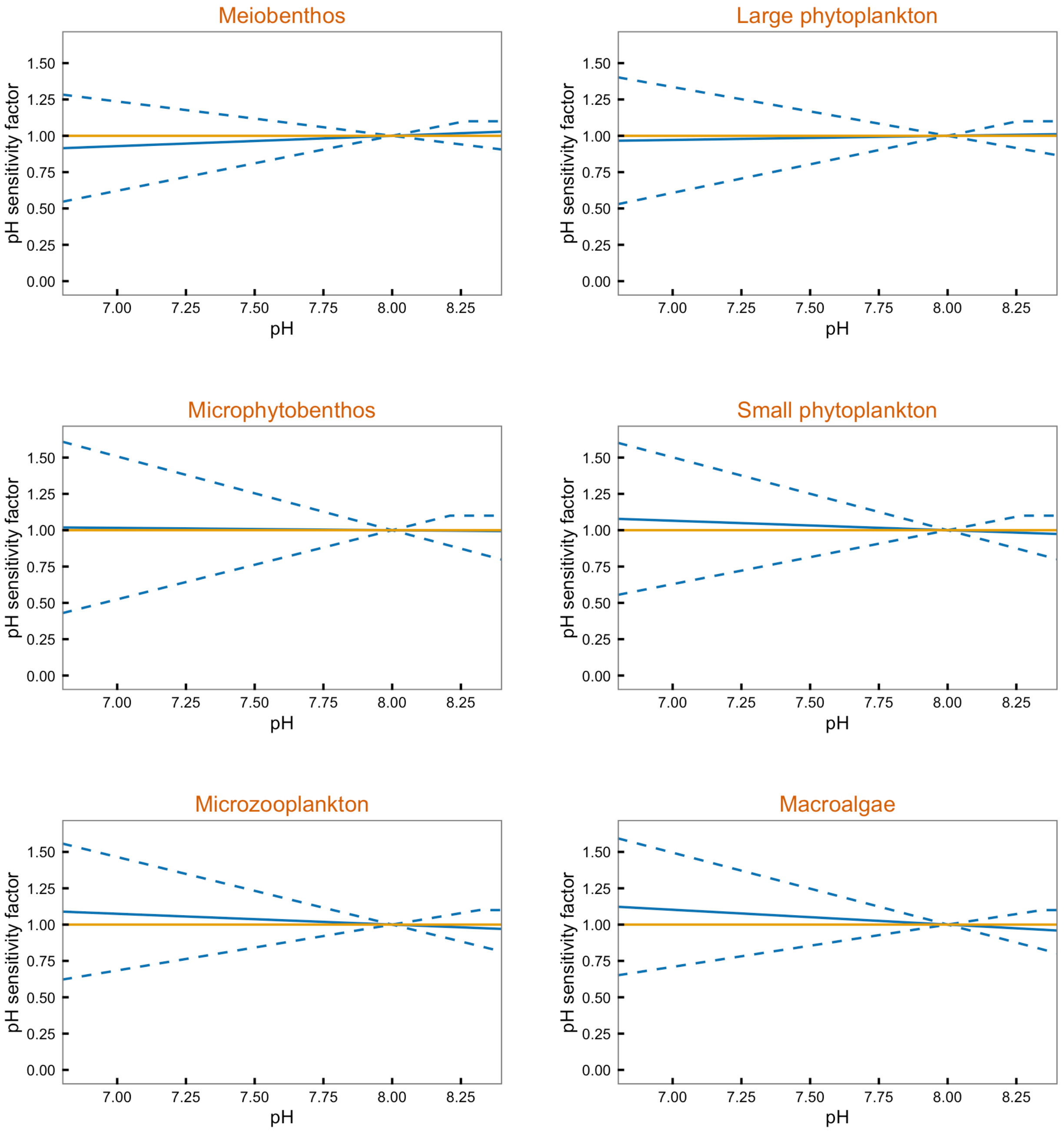
Meiobenthos
Large phytoplankton
Microphytobenthos
Small phytoplankton
Microzooplankton
Macroalgae
pH sensitivity factor
pH
1.50
1.25
1.00
0.75
0.50
0.25
0.00
7.00
7.25
7.50
7.75
8.00
8.25

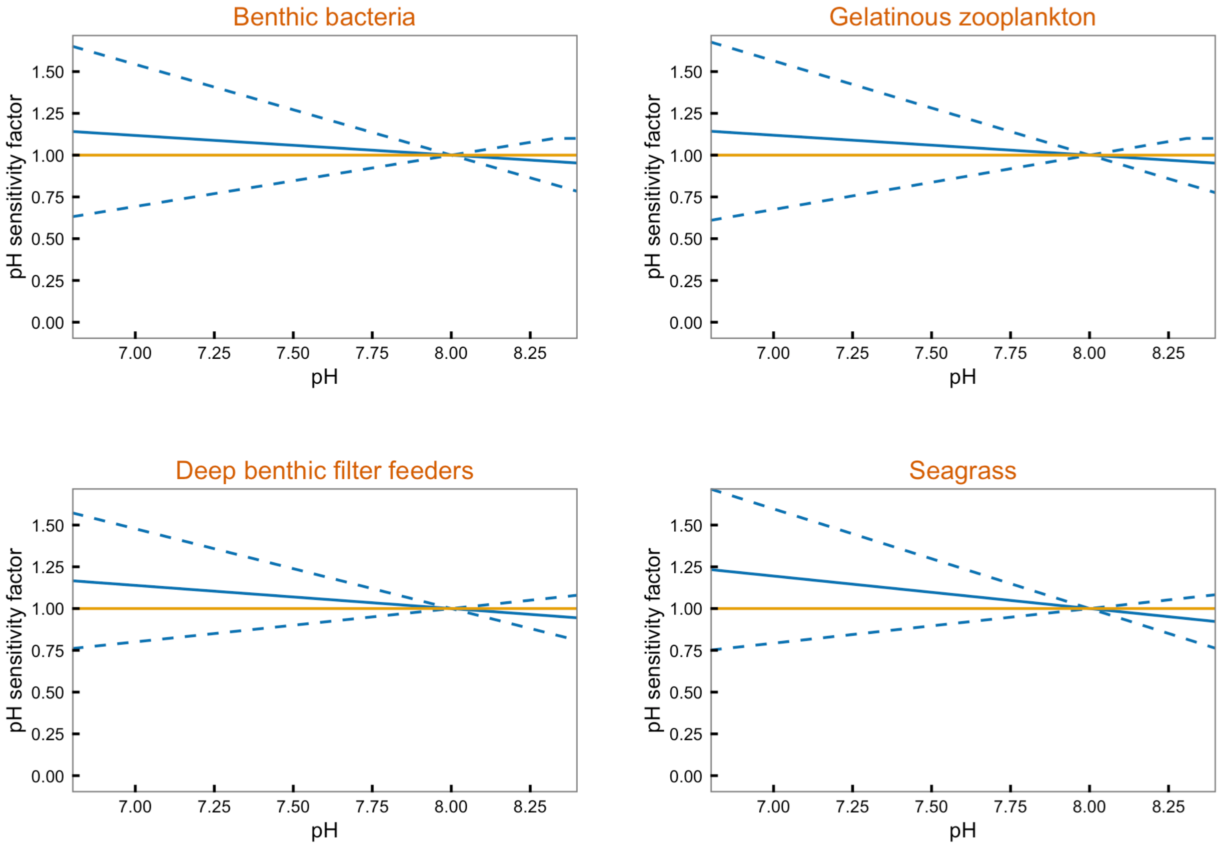

Benthic bacteria
Gelatinous zooplankton
Deep benthic filter feeders
Seagrass
pH sensitivity factor
pH
0.00
0.25
0.50
0.75
1.00
1.25
1.50
7.00
7.25
7.50
7.75
8.00
8.25
