# Supplementary material for: Estimates of the Direct Effect of Seawater pH on the Survival Rate of Species Groups in the California Current Ecosystem
Source: PLoS One. 2016 Aug 11;11(8):e0160669. doi: 10.1371/journal.pone.0160669 (PMC4981315; doi:10.1371/journal.pone.0160669)
Supplement: S2 File — (DOC) [file pone.0160669.s008.doc]

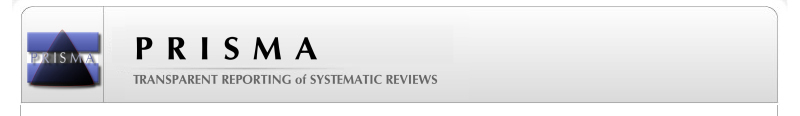
**PRISMA 2009 Flow Diagram**

**Screening**

**Included**

**Eligibility**

**Identification**

We did not keep a record of the number of papers that we found via searching the ISI Web of Science database or the EPOCA blog, nor did we keep a record of the number of duplicate papers removed when we cleaned through the records or the database entries (in some cases a paper was mistakenly entered into the database twice). To decide whether a manuscript was included in the database:

1. The distribution of its study species was assessed, and information on only temperate dwelling species were included. Studies conducted in non-temperate locations on species that exist in temperate regions were included in the database, except for tropical, reef-forming coral species, as only cold-water corals occur in the California Current ecosystem.
2. Studies that used only HCl/NaOH to develop treatment conditions were excluded.

If a manuscript passed through those three screens, it was included in the database for the meta-analysis.

Studies included in qualitative synthesis
(n = 393)

Studies included in quantitative synthesis (meta-analysis)
(n = 393 )
